# Characterising Forces Applied During the Simulated Management of Impacted Fetal Head: Pre‐Clinical Methods Study

**DOI:** 10.1111/1471-0528.70107

**Published:** 2025-12-08

**Authors:** Dawn Parris, Yang Xue, Thurga Navaseelan, Carmen Salvadores Fernandez, Maryam Javidan, Rohit Gupta, Nikolaos Salaris, Jeremy Chen, Manish K. Tiwari, Dimitrios Siassakos

**Affiliations:** ^1^ EGA Institute for Women's Health London UK; ^2^ University College Hospital: Elizabeth Garrett Anderson Wing London UK; ^3^ Nanoengineered Systems Laboratory, UCL Mechanical Engineering University College London London UK; ^4^ UCL Hawkes Institute University College London London UK

**Keywords:** caesarean section, emergency caesarean, impacted fetal head, simulation training

## Abstract

**Objective:**

Evaluate whether a sensorised surgical glove can serve as an effective training tool in management of impacted fetal head; and improvements needed. Examine location and amount of force applied through operators' hands during simulated disimpaction.

**Design:**

Feasibility study.

**Setting:**

National course of rotational vaginal birth and complex caesarean birth.

**Population:**

Obstetricians of varying seniority.

**Methods:**

Fetal head disimpaction using a model while wearing a sensorised glove. Maximum and time‐averaged force data were captured by 12 sensors, including the location of force. Thematic analysis of free‐text answers to questionnaires.

**Main Outcome Measures:**

Sensorised glove's training effectiveness, force location and amount; and themes derived from free‐text answers.

**Results:**

13 obstetricians of varying seniority participated. One was considered an expert, and their performance was used as a reference point. Fingertip sensors: highest maximum force values, consistently across participants. Palmar sensors: relatively high peak forces, greater variability. Dorsal sensors: lower forces overall, high variability. Force during fetal disimpaction was applied in brief, targeted bursts (maximum) rather than continuously (time‐averaged). Desirable improvements identified included: making the glove wireless, avoiding the need to triple glove and a standardised model.

**Conclusions:**

A sensorised glove has been developed, and participants found potential for use in training and management of impacted fetal head. There was consistent use of fingertips to deliver force, perhaps due to their precision and control. Variability in dorsal and palmar regions uncovered differences in hand positioning and technique.

## 1 Introduction

Impacted fetal head is an obstetric emergency during caesarean birth and can lead to considerable maternal and fetal trauma. Maternal risks include extended uterine incisions, postpartum haemorrhage, bladder and ureteric injury, prolonged hospital admission and hysterectomy [1, 2, 3]. Fetal injury can occur following the application of excessive force by an operator, including skull fractures, subgaleal and intracranial haemorrhage, delayed birth of an already compromised fetus resulting in hypoxic ischaemic encephalopathy, and, rarely, perinatal death [3]. The prevention of brain injury associated with impaction is a national priority [4, 5, 6]. Additional techniques may be required such as lowering the operating table, using a step, changing hands, tocolysis, vaginal disimpaction (push‐up), reverse breech and Patwardhan's technique [1, 2, 3]. The estimated incidence ranges from 1.5% to 20% of emergency caesarean births [7, 8]. It occurs in 16% of caesarean births at full dilatation, and there is a greater risk of impacted fetal head following a failed assisted vaginal birth [3]. Five per cent of caesarean births are performed at full dilatation with rates increasing, alongside a rising incidence of impacted fetal head [9].

Simulation training is well established in obstetrics and improves outcomes [10, 11]. Impacted fetal head has been introduced into national training courses, including ROBuST from the Royal College of Obstetricians and Gynaecologists (RCOG) and ART and CRAFT at University College Hospital, to equip obstetricians with the skills to manage this increasingly common emergency [12, 13, 14]. Learners practise managing impacted fetal head on models [15]. However, these models offer no real‐time performance feedback to users.

What is currently unknown is the appropriate amount of force that can be safely applied to relieve an impacted fetal head without causing fetal trauma, both with abdominal‐cephalic disimpaction and vaginal push‐up. Insufficient force risks prolonging the emergency. Conversely, excessive or inappropriately applied force through individual fingers or fingertips may risk neonatal trauma [1].

Through a collaboration between engineers and obstetricians, sensorised gloves have been developed that can detect the amount of force applied through different parts of an operator's hands. We conducted a study of sensorised gloves in the management of impacted fetal head using a widely available simulator.

### 1.1 Objectives

1. To evaluate whether sensorised surgical gloves can serve as an effective training tool in managing impacted fetal head; and improvements needed.
2. To examine the location and amount of force applied during simulated disimpaction through operators' hands.

## 2 Methods

**FIGURE 1:**
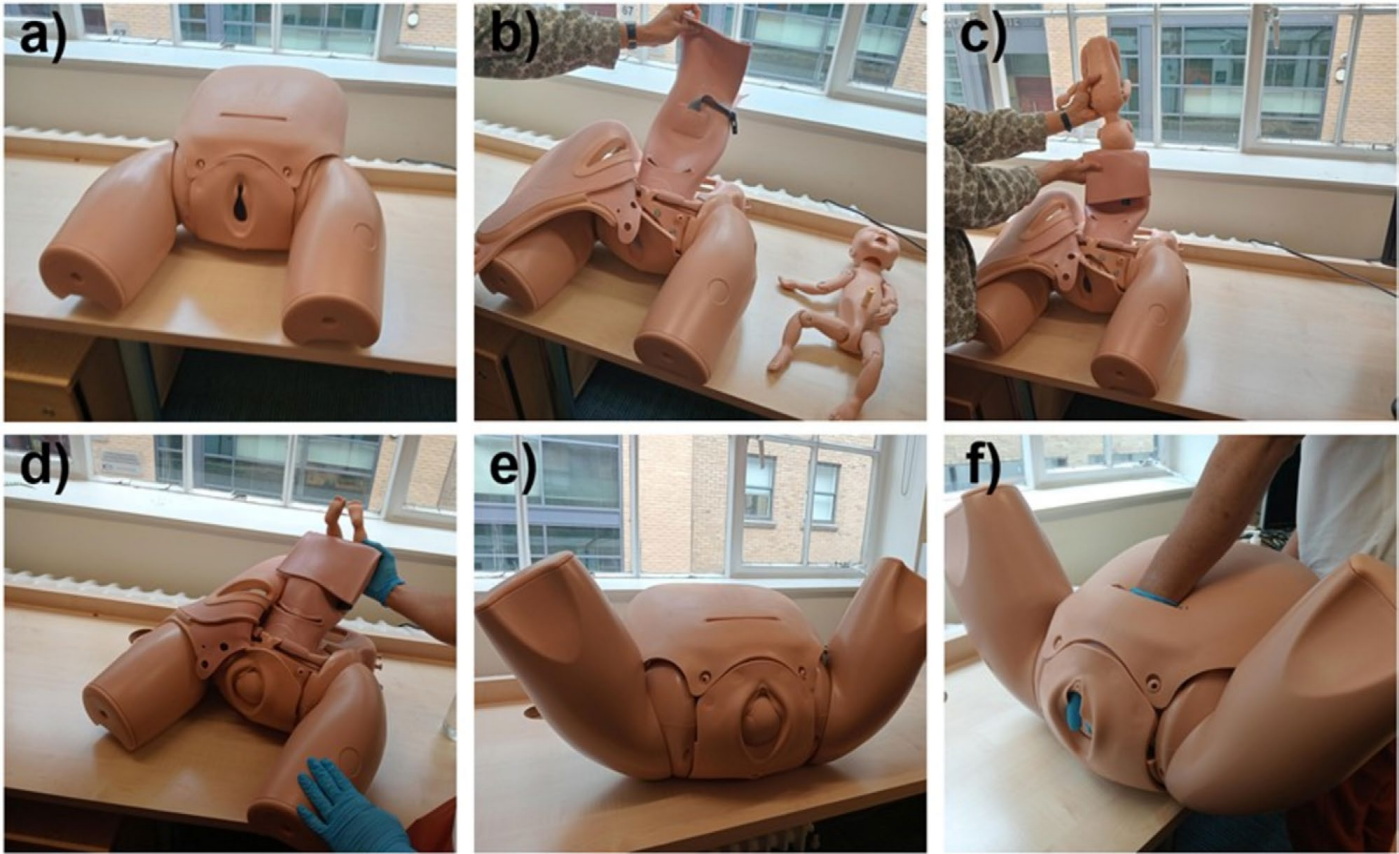
Setup and simulation of the impacted fetal head disimpaction procedure using the Limbs and Things model. (a) Anterior view of the pelvic simulator with the caesarean section module. (b) Internal view of the simulator prior to placement of the fetal mannequin. (c) Insertion of the fetal mannequin to simulate an impacted fetal head. (d) The fetal mannequin secured in the final impacted position. (e) The model positioned to simulate the intraoperative view. (f) An operator performing the simulated disimpaction manoeuvre.

At a national course of rotational vaginal birth and complex caesarean birth (ART & CRAFT) in May 2025, attendees and faculty were invited to participate in a feasibility study of a sensorised glove with a simulator of impacted fetal head (Limbs and Things [15]). Figure 1 illustrates the simulator setup and the sequence of steps used to create the disimpaction scenario and to perform the simulated disimpaction procedure. Obstetricians ranged from ST1–2 level (first 2 years of specialist training) to consultant. Their career stage, years of experience and reported task difficulty are presented in Appendix S1 for reference. The course attendees and faculty were informed that participation in research was optional, with a participant information sheet provided prior to recruitment. Participants were pseudonymised with the pseudonymisation recorded on a password‐protected spreadsheet which was securely stored on an internal repository. The pseudonymisation key was stored separately on a password‐protected hard drive. Only direct members of the research team had access to both.

**FIGURE 2:**
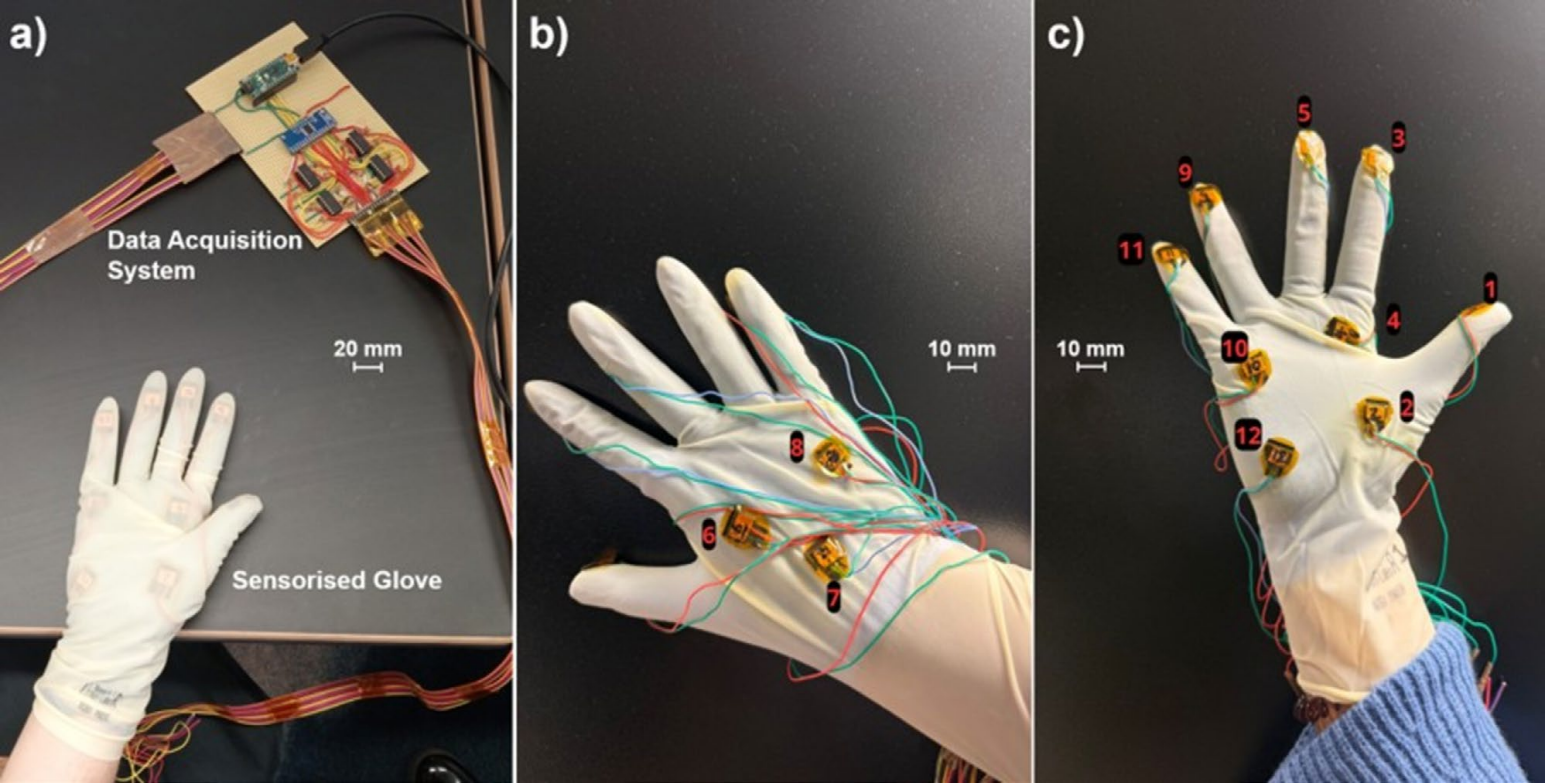
Sensorised gloves used in the study. (a) The complete sensorised glove covered by another layer of surgical glove and data acquisition system. (b) Back view of the glove (without another layer of glove on top) showing three embedded sensors, covered by an additional surgical glove layer. (c) Palm view of the glove (without another layer of glove on top) showing nine embedded sensors, also covered by an additional surgical glove layer.

On both left and right hands, participants wore three gloves: an under‐glove, a sensorised glove connected with wires to the data acquisition system, which transmitted the signals to a laptop through a serial connection, and a third over‐glove for sterility purposes, pertinent to clinical practice (Figure 2). Participants performed disimpaction using the model with abdominal‐cephalic disimpaction using their dominant hand, changing to their non‐dominant hand and vaginal push‐up, which could be performed by a second participant, also wearing a sensorised glove with under‐ and over‐gloves, as required.

**FIGURE 3:**
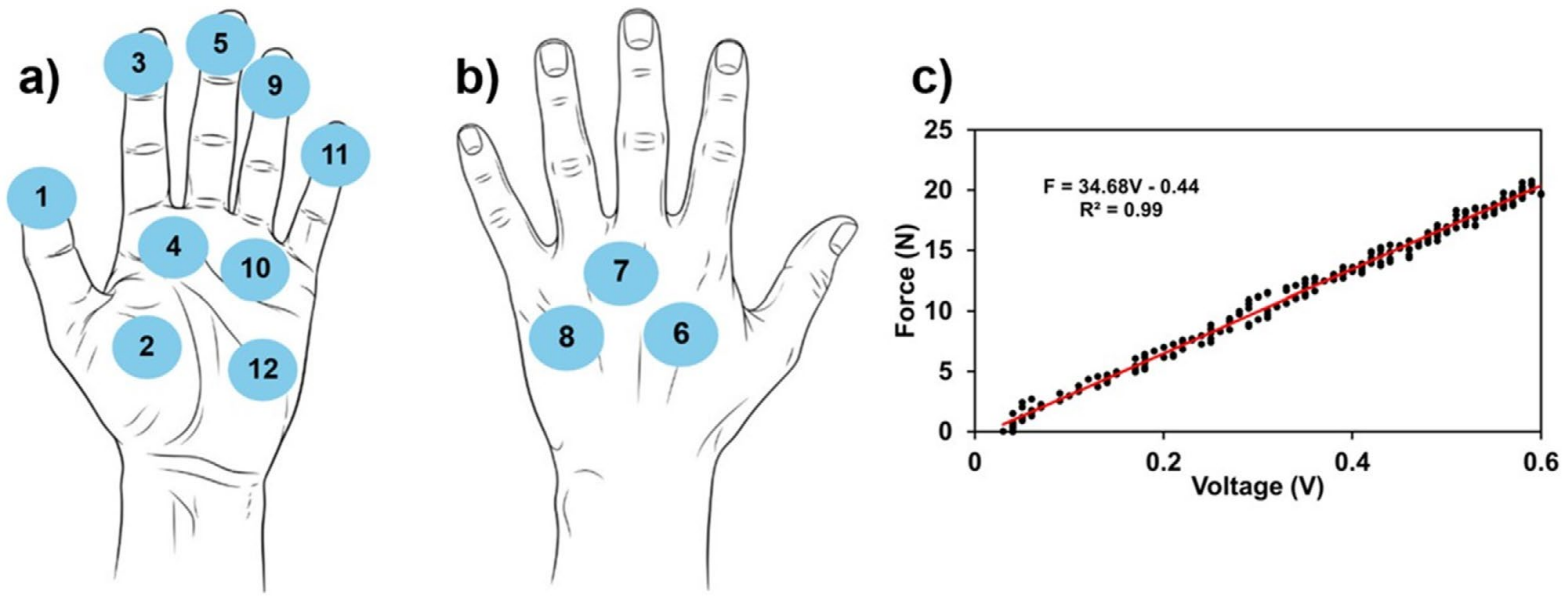
Sensor placement and example calibration curve. (a) Placement of sensors on the palm side of the glove. (b) Placement of sensors on the back of the hand. (c) Calibration curve for a selected sensor, showing the linear relationship between applied force and recorded voltage. The red line represents the linear fit, with the corresponding equation and *R* ^2^ value.

The sensorised gloves had 12 sensors placed on numerous points including the fingertips, palm and back of the hand (Figure 3a,b). These are piezoresistive force sensors, which exhibit changes in electrical resistance in response to compressive strain. The size of each sensor is 10 mm × 8 mm × 2 mm (L × W × H). For the elastic and damping characterisation, a force gauge carried by motor control with a specific rate was used to apply increasing forces to the sensors; meanwhile both force and displacement data were recorded, showing that the force is 1.95 N when the thickness deformation of the sensor is 1.4 mm (Figure S1), which demonstrated the flexibility and softness of the sensor (Appendix S2) [16]. The design and fabrication of these sensors have been detailed in previous studies [16]. Custom‐developed software was implemented to record, process and calibrate the force measurements. The system captured voltage variations corresponding to resistance changes, which were then mapped to force values through a sensor‐specific calibration process. Each sensor was individually calibrated, and yielded slight variations in fit coefficients, to relate voltage changes to reported force measurements (Figure 3c). In this way, the amount and location of force applied through a user's hand when performing impacted fetal head simulation.

The fetal model was placed in an occiput‐anterior position. Video recordings were taken of participants using the model to know when the attempt to deliver the impacted head both began and ended, to subsequently identify when force was being applied using the sensorised gloves.

The calibrated force data was collected from 12 sensors on both hands of every participant. The data was filtered and then plotted to produce individual force traces for each sensor on each surgical glove across the duration of each disimpaction attempt. ‘Attempt duration’ was defined as the time from initial contact between the participant's hand and the fetal head until the point of disimpaction completion (successful delivery of the fetal head) or, where disimpaction was not achieved, until the participant verbally indicated that further progress was not possible. In this study, all participants except participant 5 successfully completed the disimpaction. Participant 5's attempt was classified as a failure. For each participant, the maximum, minimum, range and time‐averaged force were calculated for each sensor, and the medians of these values. The first (Q1) and third (Q3) quartiles were also determined to assess variability. Box‐whisker plots were generated to visualise the distribution of both maximum and time‐averaged forces across participants.

To establish a reference benchmark for force application, an expert obstetrician (participant 12) was asked to perform the procedure with what they considered to be the maximum force that should be applied. The maximum force recorded during this expert trial was used as a comparative benchmark against other participants' force traces, highlighting instances where this reference value was exceeded. The expert was identified as a senior consultant with clinical and research experience in simulation training, complex assisted vaginal birth and impacted fetal head. The other participants were unaware that this reference value had been set during the study.

Force traces were reviewed alongside synchronised video recordings for each participant to identify any irregularities, notable patterns, or technique‐specific variations.

One vaginal push‐up was performed by a second participant wearing a sensorised glove, while the first participant simultaneously performed disimpaction using their dominant hand. As this configuration differed from the single‐operator approach used by others, data from this instance were excluded from group statistical analyses and were analysed separately.

All signal processing, calibration, filtering and force trace plotting were performed in MATLAB R2025a. All statistical summaries and boxplots were generated using IBM SPSS Statistics 30 Build 172, with outcome measures reported as median ± interquartile range (IQR). Statistical analysis of differences in forces was performed using nonparametric tests (Mann–Whitney *U* test), with *p* < 0.05 deemed statistically significant.

Participants were asked to complete questionnaires following use of the system with free‐text questions (Appendix S3) to evaluate desirable improvements. The participants' level of seniority in obstetrics, years of experience and hand dominance were also recorded. The free‐text questions underwent thematic analysis by one of the researchers using Braun and Clarke's method [17].

Ethical approval was granted by the University College London Institute for Women's Health Research Ethics Committee, ID 1065.

## 3 Results

### 3.1 Participant Characteristics

Thirteen participants were recruited: one ST1–2, five ST3–5, five ST6–7, a total of 11 resident doctors and two consultants (attendings). Two participants had 0–5 years' experience in obstetrics, seven participants had 6–10 years' experience, and four had over 10 years' experience. Of the 12 participants who responded in the questionnaire, all were right‐hand dominant.

### 3.2 Force Data

The force traces recorded from each sensor on the sensorised surgical glove (for both hands) during the disimpaction procedure are presented in Figures S2–S27. All participants in the study used their right hand as the dominant hand. Figure 4 shows a representative example of these force traces of the dominant hand for a selected participant (participant 10). The red dashed line represents the maximum force recorded by each sensor on the dominant hand of an expert obstetrician, who performed the procedure using what they judged to be the upper limit of acceptable force in clinical practice. This should be interpreted as an expert benchmark rather than a validated safety threshold. In Figure 4, sensors 3, 5, 9 and 11 are displayed, which show the highest median maximum forces across participants. Full force metrics for all sensors and participants can be found in Tables S2–S14.

**FIGURE 4:**
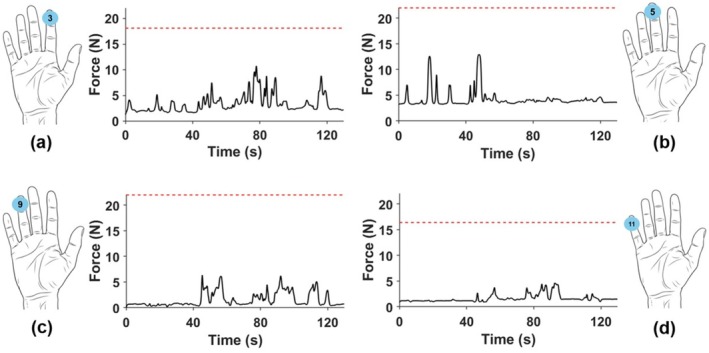
Example force traces from selected sensors (a) Sensor 3, (b) Sensor 5, (c) Sensor 9 and (d) Sensor 11 for Participant 10. The red dashed line represents the expert‐defined safety threshold.

The red dashed expert benchmark line in the force traces was only included for the dominant hand, as all participants used this hand to apply the primary force during the procedure. The use of the non‐dominant hand varied between individuals—to retract the caesarean incision, to provide additional torque or balance, or to press or pull on the dominant hand to assist. Due to this variability, force thresholds from the non‐dominant hand were excluded from the main analysis.

Summary statistics of the measured forces during the disimpaction procedure are presented in Table 1, which includes the median maximum force and median time‐averaged force (with interquartile ranges) for each sensor on both the dominant and non‐dominant hands. Individual participant‐level data, including force traces, maximum, minimum, range and time‐averaged forces for each sensor, are available in Appendix S4.

**TABLE 1:** Summary of measured force data by the sensorised glove during the disimpaction procedure. Values are presented as median (interquartile range, IQR) for all participants combined. Force is measured in newtons (N). “Median maximum force” represents the highest instantaneous force recorded for each sensor per participant, while “median time‐averaged force” represents the median of the mean force applied over the duration of each disimpaction attempt.

	Median maximum force (IQR) (N)	Median time‐averaged force (IQR) (N)
Dominant hand		
Sensor 1	10.38 (16.21–4.20)	3.09 (4.51–0.71)
Sensor 2	11.67 (21.62–0.30)	1.81 (4.98–0.26)
Sensor 3	15.26 (17.66–11.65)	4.33 (7.51–2.64)
Sensor 4	12.04 (21.65–7.86)	1.96 (2.44–1.32)
Sensor 5	21.70 (21.90–14.82)	5.01 (6.96–4.24)
Sensor 6	7.14 (16.18–0.61)	0.44 (4.03–0.04)
Sensor 7	4.41 (10.96 2.99)	1.20 (2.65 0.26)
Sensor 8	4.34 (19.60 0.27)	2.17 (3.63 0.01)
Sensor 9	13.63 (19.61 8.84)	3.02 (6.39 1.83)
Sensor 10	10.00 (17.31 3.70)	1.27 (2.15 0.14)
Sensor 11	17.56 (21.60 13.12)	3.98 (5.86 2.18)
Sensor 12	7.35 (12.69 0.88)	0.63 (3.56 0.04)
Non‐dominant hand		
Sensor 1	6.21 (14.08–2.13)	2.08 (3.75–0.43)
Sensor 2	4.23 (11.51–1.12)	1.26 (1.99–0.18)
Sensor 3	14.63 (20.58–9.03)	3.23 (3.97–2.53)
Sensor 4	6.49 (19.90–0.38)	0.44 (5.61–0.01)
Sensor 5	10.62 (20.44–4.60)	1.53 (6.10–0.40)
Sensor 6	4.32 (14.54–0.00)	1.03 (5.16–0.00)
Sensor 7	2.72 (4.92–2.12)	2.02 (3.02–1.03)
Sensor 8	4.79 (12.23–0.70)	2.77 (5.96–0.19)
Sensor 9	7.42 (18.56–3.48)	1.22 (3.99–0.41)
Sensor 10	12.47 (21.94–6.54)	5.56 (7.92–0.50)
Sensor 11	6.45 (15.36–2.58)	2.16 (4.29–1.47)
Sensor 12	4.82 (12.45–2.33)	1.47 (3.39–0.22)

**FIGURE 5:**
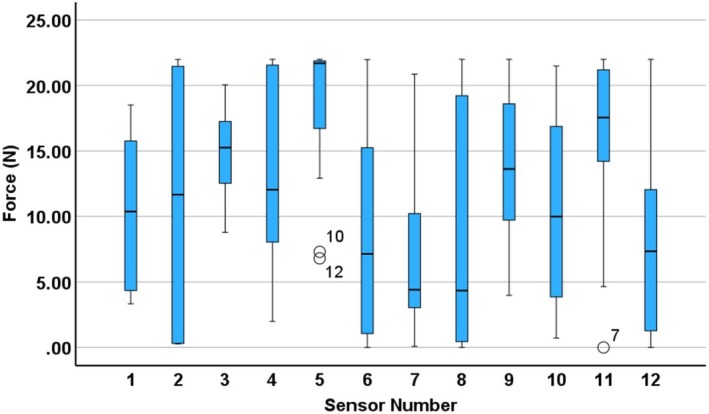
Boxplot of maximum force values per participant for each sensor on the dominant (right) hand, with circles representing mild outliers. The numbers next to the outliers indicate the participant number corresponding to that data point.

**FIGURE 6:**
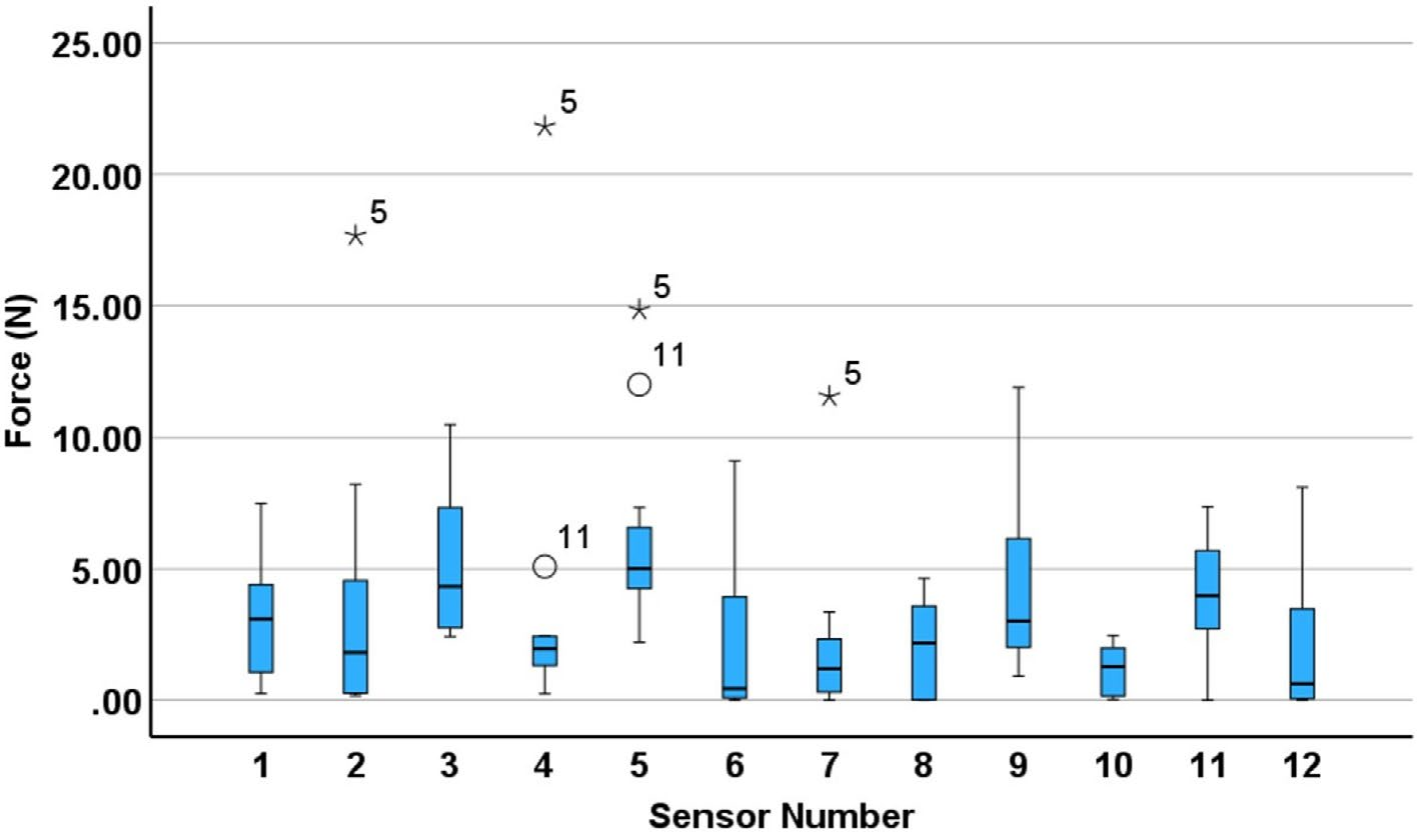
Boxplot of time‐averaged force values per participant for each sensor on the dominant (right) hand, with circles and asterisks representing mild and extreme outliers respectively. The numbers next to the outliers indicate the participant number corresponding to that data point.

The variability in force distribution across participants is illustrated in Figures 5 and 6. Figure 5 presents the maximum force values recorded for each sensor on the dominant hand, while Figure 6 displays time‐averaged force values for the same sensors.

Sensors 3, 5, 9 and 11 (located on the fingertips—Figure 3) recorded some of the highest maximum force values, suggesting that fingertips play a central role in applying strong, localised force during fetal disimpaction. These sensors also exhibited relatively low variability across participants (Figure 4), indicating a consistent use of fingertips during the maneuver, perhaps due to their precision and control.

In contrast, palmar sensors (such as 4, 10 and 12—Figure 3) also recorded elevated peak forces but with greater variability (12.04 N (21.65 N–7.86 N) at sensor 4, 10.00 N (17.31 N–3.7 N) at sensor 10, 7.35 N (12.69 N–0.88 N) at sensor 12), suggesting differences in how the palm is engaged, perhaps when stabilising or applying higher contact area pressure. Dorsal sensors (6–8—Figure 3) generally showed lower forces overall but also high variability (7.14 N (16.18 N–0.61 N) at sensor 6, 4.41 N (10.96 N–2.99 N) at sensor 7, 4.34 N (19.60 N–0.27 N) at sensor 8). The dorsum of the hand seems to be less involved, likely serving a supportive or passive role. These variabilities across dorsal and palmar regions likely reflect differences in hand positioning and technique across clinicians.

The notable difference between maximum (Figure 5) and time‐averaged (Figure 6) force values indicates that force during fetal disimpaction was applied in brief, targeted bursts rather than continuously. This pattern may reflect the controlled and intermittent nature of the manoeuvre, thereby minimising prolonged pressure on maternal or fetal tissues.

Participant 5 was a clear outlier in the time‐averaged force distribution (Figure 6). They were the only participant unable to complete the disimpaction, despite applying high levels of force. Thus, exceeding the expert‐defined benchmark may not only indicate potentially excessive force, but may also be associated with difficulty in successfully achieving disimpaction.

### 3.3 Force Data Assisted Disimpaction

The participant who received assistance (participant 3 with vaginal push‐up by participant 4) was analysed separately. Participant 3 applied higher forces on sensors 4 (15.94 N vs. 12.04 N), 5 (21.99 N vs. 21.70 N), 7 (5.38 N vs. 4.41 N), 11 (22 N vs. 17.56 N) and 12 (21.98 N vs. 7.35 N) compared to the group median maximum forces recorded by participants who completed the procedure independently. Similarly, the time‐averaged forces on sensors 4 (2.99 N vs. 1.96 N), 5 (12.5 N vs. 5.01 N), 6 (1.04 N vs. 0.44 N), 7 (1.81 N vs. 1.20 N), 9 (6.96 N vs. 3.02 N), 11 (14.29 N vs. 3.98 N) and 12 (3.82 N vs. 0.63 N) were also higher than the median time‐averaged forces observed in participants performing the procedure without assistance (Tables S1–S14, Figure S8).

In contrast, participant 4 who performed the vaginal push‐up generally applied lower maximum and time‐averaged forces than the median values recorded from those performing disimpaction independently. The only exceptions were sensors 3 (4.82 N vs. 4.33 N), 5 (5.18 N vs. 5.01 N) and 9 (3.38 N vs. 3.02 N), where the assisting participant applied slightly higher time‐averaged forces, and sensor 12, where the maximum force slightly exceeded the group median (Table S5 and Figure S9).

The increased forces observed in participant 3 may suggest several possible contributing factors. It is possible that the presence of assistance altered the perception of resistance or reduced tactile feedback, leading the operator to apply greater force. The added vaginal pressure may also have increased the overall resistance, prompting a compensatory response. Participant 4 might have applied lower forces due to the limited mechanical advantage of the vaginal route, a cautious approach, or a perception of playing a secondary role. The time‐averaged forces exerted by assisting participant 4 at all sensors were below 5.2 N. This may indicate that the dynamics of force application could change when disimpaction is performed collaboratively, which may be important to consider in future training or procedural coordination.

### 3.4 Thematic Analysis

Based on participant feedback, the glove system was generally seen as a promising and innovative tool, with users happy with the comfort, ease of use, potential for improving safety and ability to provide haptic and force feedback. Many appreciated its novelty and contribution to training, particularly for complex scenarios. However, there are some concerns such as difficulties with reproducibility and standardisation, reduced tactile sensitivity when wearing multiple layers of gloves, discomfort from the wires coming out of the gloves. Overall, while participants acknowledged the glove's potential, they emphasised the need for refinement, particularly around usability, and freedom of movement.

## 4 Discussion

### 4.1 Main Findings

A low‐cost sensorised glove can capture detailed data on the techniques and forces used during simulated fetal head disimpaction. These data provide insights into both common and variable aspects of technique: consistent use of all five fingertips to spread and apply force in brief bursts, alongside variation in the use of the palm and dorsum of the hand. While participants informally reported that the glove raised their awareness of hand use and applied force, we did not formally evaluate its impact on training outcomes. Future work is required to assess the glove's role in training more directly by comparing subjective estimates of force with actual measured forces or assessing changes in confidence and skill after repeated use with the glove active versus inactive.

We found that participants perceived the sensorised gloves had potential for use in training, for understanding the technique and forces involved, and in the future for providing real‐time feedback. It appears that some aspects of the disimpaction technique are standardised, particularly the use of all five fingertips to spread and apply force in brief bursts, whereas other aspects vary between clinicians, for example the use of the palm and dorsum of the hand.

### 4.2 Strengths and Limitations

Sensorised gloves have been developed and successfully used in models to detect fetal position and obstetric anal sphincter injuries to have a level of accuracy [18]. This is the first study of sensorised gloves at any point during the simulation of caesarean birth, including the management of an impacted fetal head, and is therefore highly novel. The sensorised gloves are low‐cost, approximately $1 USD, so they can have applications in low‐resource settings where obstructed labour is a major cause of maternal death [18, 19, 20, 21].

The sample size of participants is relatively small, and not all questionnaires were completed. The participants were attendees and faculty at a course where they practised the management of impacted fetal head. Ideally, the study could have been conducted with force data collected prior to and after practising on the model during the course to compare the teaching's impact, which will be done in future work.

Furthermore, using quantitative features extracted from the calibrated sensor output time series and basic machine learning models, we were unable to distinguish clinicians based on seniority, and demonstrated a modest predictive ability for classifying the subjective experience of task difficulty. These results in Appendix S5 highlight the critical importance of accounting for perceived task difficulty as a key confounding variable or stratification factor in future studies of motor skill assessment. This is planned for the future.

The accuracy of the results of the force data is limited by the realism of the model—albeit a model widely available and commonly used including at the RCOG ROBuST course [12]. How force is applied in vivo may differ from simulation, as the materials which comprise the model are different to both maternal and fetal tissues. Likewise, in a more stressful situation with real patients, force may be applied differently as well.

The fetal model was always placed in an occiput‐anterior position to standardise the impaction scenario. As fetal malposition is an independent risk for impacted fetal head, it would be interesting to see how forces may differ with various fetal head orientations, and varying levels of intended (as opposed to perceived) difficulty [22]. Likewise, conducting the study with differently sized fetal heads or with varying degrees of caput or moulding, may also provide useful results.

### 4.3 Interpretation

Other novel devices are being developed to assist disimpaction. Two recent studies have been conducted to understand fetal head elevation with novel devices to aid impacted fetal head—Fetal Pillow and the Tydeman Tube [23, 24]. The Tydeman Tube has been found to provide greater elevation of the fetal head than either the Fetal Pillow or digital elevation [23]. However, unlike sensorised surgical gloves, they cannot provide real‐time feedback on an operator's technique; whereas with further refinement and in vivo studies, the sensorised gloves may help determine appropriate force thresholds.

Given the increasing rates of caesarean birth globally, including caesarean birth at full dilatation, more needs to be done to improve the management of impacted fetal head [1, 25, 26, 27]. While an improvement in skills and confidence in assisted vaginal birth, potentially reduces how often caesarean births at full dilatation are performed, over 50% of cases of impacted fetal head occur prior to full cervical dilatation, so not fully addressing the problem [1].

The next steps for sensorised gloves involve further refinement and validation within simulation environments, as well as testing in real life. Specifically, studies are needed to establish consistency across different glove prototypes, to assess the reproducibility of force measurements, and to compare outputs with an expert pool of clinical judgement. The force benchmark was derived from one expert obstetrician, therefore representing an expert reference point rather than a validated safety threshold; further work with multiple experts and larger datasets will be required to establish more generalisable limits. Once validated in simulation, the glove could be incorporated into structured training curricula to evaluate its effectiveness in enhancing skill acquisition among obstetric trainees. After such staged development and appropriate regulatory and ethical approvals, studies in clinical settings, including during the management of impacted fetal heads, must be considered. In this way, the glove may progressively contribute to a better understanding of the forces applied during disimpaction and, in the longer term, support improvements in both training and clinical practice.

## 5 Conclusion

A sensorised glove has been developed, which is favourable with obstetricians for potential use in the management of impacted fetal head, at least in training to begin with. The glove indicated participant technique and force use during simulation of fetal head and allowed quantitative comparison against standardised practice and expert performance. Desirable improvements include making the glove wireless and standardising the model for disimpaction. There are aspirations to conduct an in vivo study before the sensorised glove can be used in training and clinical practice to guide clinicians, ultimately aiming to reduce maternal and neonatal harm.

## Author Contributions

D.P., Y.X., T.N., M.J. and C.S.F.: conception of study, planning, carrying out, analysing, writing up of the work and final approval of the paper. R.G. and J.C.: planning, analysing, revision of the work and final approval of the paper. M.K.T. and D.S.: conception of study, planning, writing up of the work and final approval of the paper.

## Funding

Support from the European Research Council (ERC) Proof‐of‐Concept Grant, SaferBirths; InspiringFuture ERC Consolidator Fellowship selected by the ERC, funded by UKRI Horizon Europe Guarantee (EP/X023974/1); and the Royal Society Wolfson Fellowship are gratefully acknowledged. M.J. studentship no: 2901951 (related to EP/W524335/1). T.N. studentship no: 2925311 (related to EP/Y034929/1). M.K.T. Royal Society Wolfson Fellowship: RSWF\R3\193 013. ERC Proof‐of‐concept SaferBirths: 101189504.

## Ethics Statement

Ethical approval was granted on 8 May 2025 from UCL Institute of Women's Health REC (Ethics ID: 1065).

## Consent

The authors have nothing to report.

## Conflicts of Interest

D.P.: Organiser of ART and CRAFT course, Clinical Research Fellow for the ROTATE trial, faculty for ROBuST. D.S.: Organiser of ART and CRAFT course, Chief Investigator for the ROTATE trial, delegate of ROBuST. The remaining authors declare no conflicts of interest.

## Supporting information


**Table S1:** Participant information.
**Table S2:** Force data acquired from participant 1.
**Table S3:** Force data acquired from participant 2.
**Table S4:** Force data acquired from participant 3.
**Table S5:**: Force data acquired from participant 3, right hand, assisted by participant 4, right hand.
**Table S6:** Force data acquired from participant 5.
**Table S7:** Force data acquired from participant 6.
**Table S8:** Force data acquired from participant 7.
**Table S9:** Force data acquired from participant 8.
**Table S10:** Force data acquired from participant 9.
**Table S11:** Force data acquired from participant 10.
**Table S12:** Force data acquired from participant 11.
**Table S13:** Force data acquired from participant 12.
**Table S14:** Force data acquired from participant 13.
**Figure S1:** Displacement vs. force plot of melamine‐foam‐based sensors. A motor control with a specific rate is used, allowing the force gauge with a cylinder tip to gradually apply forces to the foam. The foam size is 10 mm × 8 mm × 2 mm (L × W × H). And the plot demonstrated that the force is 1.95 N when the thickness deformation of the foam is 1.4 mm.
**Figure S2:** Force sensor readings for participant 1—Right hand. The red dashed line represents the expert‐defined reference benchmark.
**Figure S3:** Force sensor readings for participant 1—Left hand.
**Figure S4:** Force sensor readings for participant 2—Right hand. The red dashed line represents the expert‐defined reference benchmark.
**Figure S5:** Force sensor readings for participant 2—Left hand.
**Figure S6:** Force sensor readings for participant 3—Right hand. The red dashed line represents the expert‐defined reference benchmark.
**Figure S7:** Force sensor readings for participant 3—Left hand.
**Figure S8:** Force sensor readings for participant 3—Right hand, assisted by participant 4. The red dashed line represents the expert‐defined reference benchmark.
**Figure S9:** Force sensor readings for participant 4—Right Hand, assisting participant 3.
**Figure S10:** Force sensor readings for participant 5—Right hand. The red dashed line represents the expert‐defined reference benchmark.
**Figure S11:** Force sensor readings for participant 5—Left hand.
**Figure S12:** Force sensor readings for participant 6—Right hand. The red dashed line represents the expert‐defined reference benchmark.
**Figure S13:** Force sensor readings for participant 6—Left hand.
**Figure S14:** Force sensor readings for participant 7—Right hand. The red dashed line represents the expert‐defined reference benchmark.
**Figure S15:** Force sensor readings for participant 7—Left hand.
**Figure S16:** Force sensor readings for participant 8—Right hand. The red dashed line represents the expert‐defined reference benchmark.
**Figure S17:** Force sensor readings for participant 8—Left hand.
**Figure S18:** Force sensor readings for participant 9—Right hand. The red dashed line represents the expert‐defined reference benchmark.
**Figure S19:** Force sensor readings for participant 9—Left hand.
**Figure S20:** Force sensor readings for participant 10—Right hand. The red dashed line represents the expert‐defined reference benchmark.
**Figure S21:** Force sensor readings for participant 10—Left hand.
**Figure S22:** Force sensor readings for participant 11—Right hand. The red dashed line represents the expert‐defined reference benchmark.
**Figure S23:** Force sensor readings for participant 11—Left hand.
**Figure S24:** Force sensor readings for participant 12—Right hand.
**Figure S25:** Force sensor readings for participant 12—Left hand.
**Figure S26:** Force sensor readings for participant 13—Right hand. The red dashed line represents the expert‐defined reference benchmark.
**Figure S27:** Force sensor readings for participant 13—Left hand.
**Figure S28:** Principal component analysis (PCA) of all performance features stratified by (a) Clinician Seniority, where the participants where split into two groups Junior and Senior from the original 4 (ST1‐2, ST3‐5, ST6‐7, Consultant); and (b) Perceived Difficulty, where the participants were split into two groups of Easy and Hard from the original 4 (Less Force/Easy, Normal, Normal hard, Hard). The two principal components (PCs) were derived from all standardised performance features. Each point represents an individual participant, positioned according to their composite performance score. Participants are coloured by their seniority level and self‐reported perceived difficulty of the disimpaction task.
**Figure S29:** Comparative Machine Learning Performance for Predicting Perceived Difficulty. Receiver Operating Characteristic (ROC) curves for Logistic Regression and Random Forest models evaluated using a Leave‐One‐Out Cross‐Validation (LOOCV) and Bootstrapping strategies. The *y*‐axis represents the True Positive Rate (Sensitivity), and the x‐axis represents the False Positive Rate (1 − Specificity). The Area Under the Curve (AUC) quantifies the model's overall ability to distinguish between the ‘Hard’ and ‘Easy’ perceived difficulty classes. The classifiers yielded an AUC ROC of 0.82 and 0.91 for the Logistic Regression and Random Forest models, respectively. The dashed line represents the performance of a random‐chance classifier (AUC = 0.5).

## Data Availability

The data that support the findings of this study are available on request from the corresponding author. The data are not publicly available due to privacy or ethical restrictions.
