# Supplementary material for: Characterising Forces Applied During the Simulated Management of Impacted Fetal Head: Pre‐Clinical Methods Study
Source: BJOG. 2025 Dec 8;133(6):1176–85. doi: 10.1111/1471-0528.70107 (PMC13040427; doi:10.1111/1471-0528.70107)
Supplement: Supplementary file 1 — Table S1: Participant information. Table S2: Force data acquired from participant 1. Table S3: Force data acquired from participant 2. Table S4: Force data acquired from participant 3. Table S5:: Force data acquired from participant 3, right hand, assisted by participant 4, right hand. Table S6: Force data acquired from participant 5. Table S7: Force data acquired from participant 6. Table S8: Force data acquired from participant 7. Table S9: Force data acquired from participant 8. Table S10: Force data acquired from participant 9. Table S11: Force data acquired from participant 10. Table S12: Force data acquired from participant 11. Table S13: Force data acquired from participant 12. Table S14: Force data acquired from participant 13. Figure S1: Displacement vs. force plot of melamine‐foam‐based sensors. A motor control with a specific rate is used, allowing the force gauge with a cylinder tip to gradually apply forces to the foam. The foam size is 10 mm × 8 mm × 2 mm (L × W × H). And the plot demonstrated that the force is 1.95 N when the thickness deformation of the foam is 1.4 mm. Figure S2: Force sensor readings for participant 1—Right hand. The red dashed line represents the expert‐defined reference benchmark. Figure S3: Force sensor readings for participant 1—Left hand. Figure S4: Force sensor readings for participant 2—Right hand. The red dashed line represents the expert‐defined reference benchmark. Figure S5: Force sensor readings for participant 2—Left hand. Figure S6: Force sensor readings for participant 3—Right hand. The red dashed line represents the expert‐defined reference benchmark. Figure S7: Force sensor readings for participant 3—Left hand. Figure S8: Force sensor readings for participant 3—Right hand, assisted by participant 4. The red dashed line represents the expert‐defined reference benchmark. Figure S9: Force sensor readings for participant 4—Right Hand, assisting participant 3. Figure S10: Force sensor readings for participant 5—Right han [file BJO-133-1176-s001.docx]

**Appendix 1.** Participant information

| **Participant ID** | **Career Level** | **Number of years' experience** | **Hand Dominance** | **Difficulty** |
| --- | --- | --- | --- | --- |
| DI1 | ST6-7 | 10+ | Right | Less force than in real life |
| DI2 | ST3-5 | 6-10 | Right | Less force than in real life |
| DI3 | ST6-7 | 6-10 | Right |  |
| DI4 | ST1-2 | 0-5 | Right |  |
| DI5 | ST3-5 | 6-10 | Right | Difficult |
| DI6 | ST3-5 | 6-10 | Right | Normal |
| DI7 | ST3-5 | 0-5 | Right | Normal |
| DI8 | Consultant | 10+ |  | Normal hard |

| DI9 | ST6-7 | 6-10 | Right | Difficult |
| --- | --- | --- | --- | --- |
| DI10 | ST3-5 | 10+ | Right | Difficult |
| DI11 | ST6-7 | 6-10 | Right | Difficult |
| D12 | Consultant | 10+ | Right | Difficult - upper limit of difficulty |
| DI13 | ST6-7 | 6-10 | Right | Normal hard |

**Table S1.** Participant information.

**Appendix 2.** Displacement vs. Force plot of the melamine-foam-based piezoresistive sensors.


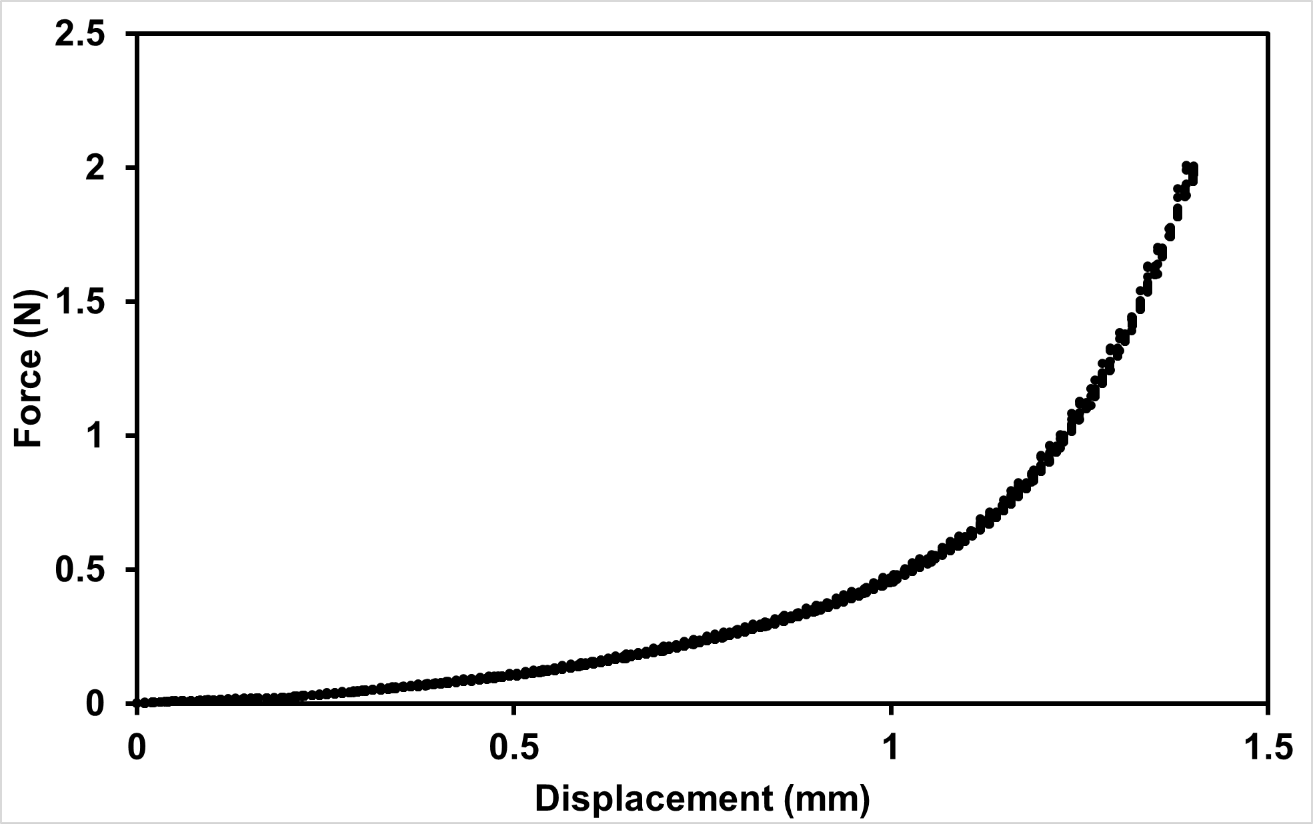


**Figure S1.** Displacement vs force plot of melamine-foam-based sensors. A motor control with a specific rate is used, allowing the force gauge with a cylinder tip to gradually apply forces to the foam. The foam size is 10 mm * 8 mm * 2 mm (L * W * H). And the plot demonstrated that the force is 1.95 N when the thickness deformation of the foam is

1.4 mm.

**Appendix 3.** Free-text Questionnaire

## Participant ID:

**Career level (Please circle)**

Student / ST1-2 or equivalent / ST3-5 or equivalent / ST6-7 or equivalent / consultant

## Number of years’ experience in Obstetrics and Gynaecology (please circle)

Student / 0 – 5 / 6 – 10 / 10 +

## Hand dominance (please circle):

Left hand dominant / Right hand dominant

**What did you like about the system?**

**What didn’t you like about the system?**

**What would you change about the system technically?**

**What would you change/add to the system to roll out clinically or training?**

**Appendix 4.** Force Traces and Force Metrics from Participants 1-13.


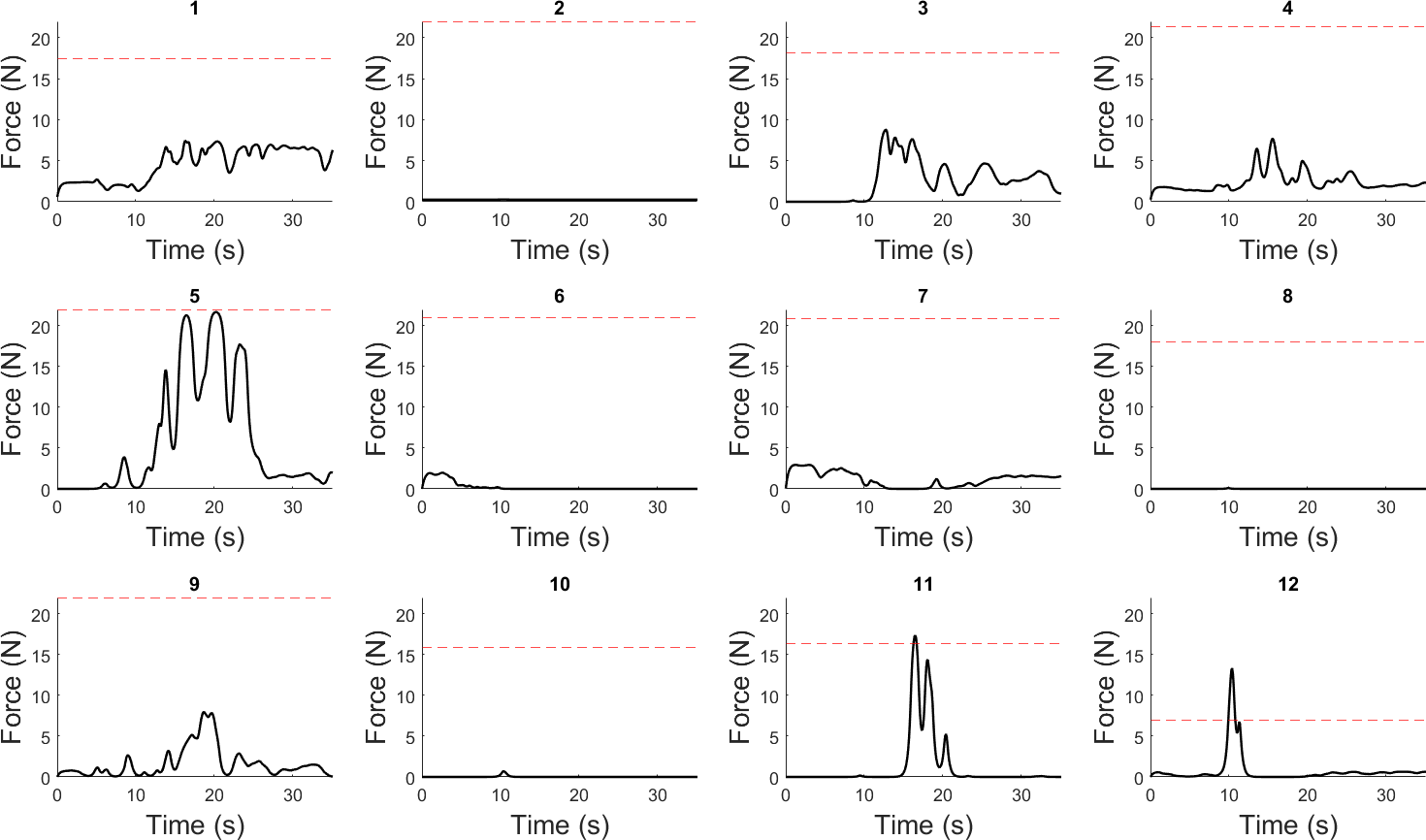


**Figure S2.** Force Sensor Readings for Participant 1 – Right Hand. The red dashed line represents the expert-defined reference benchmark.


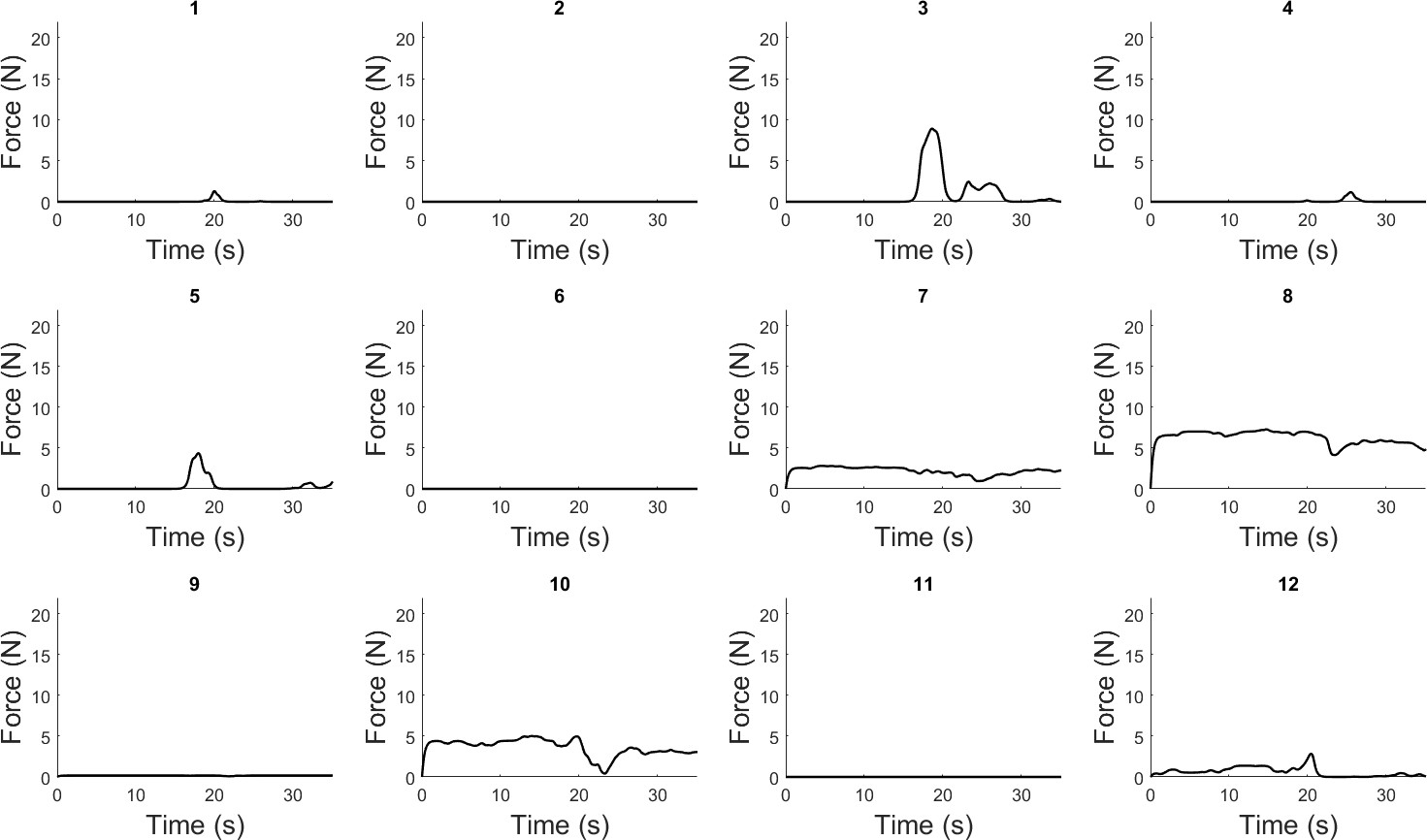


**Figure S3.** Force Sensor Readings for Participant 1 – Left Hand.


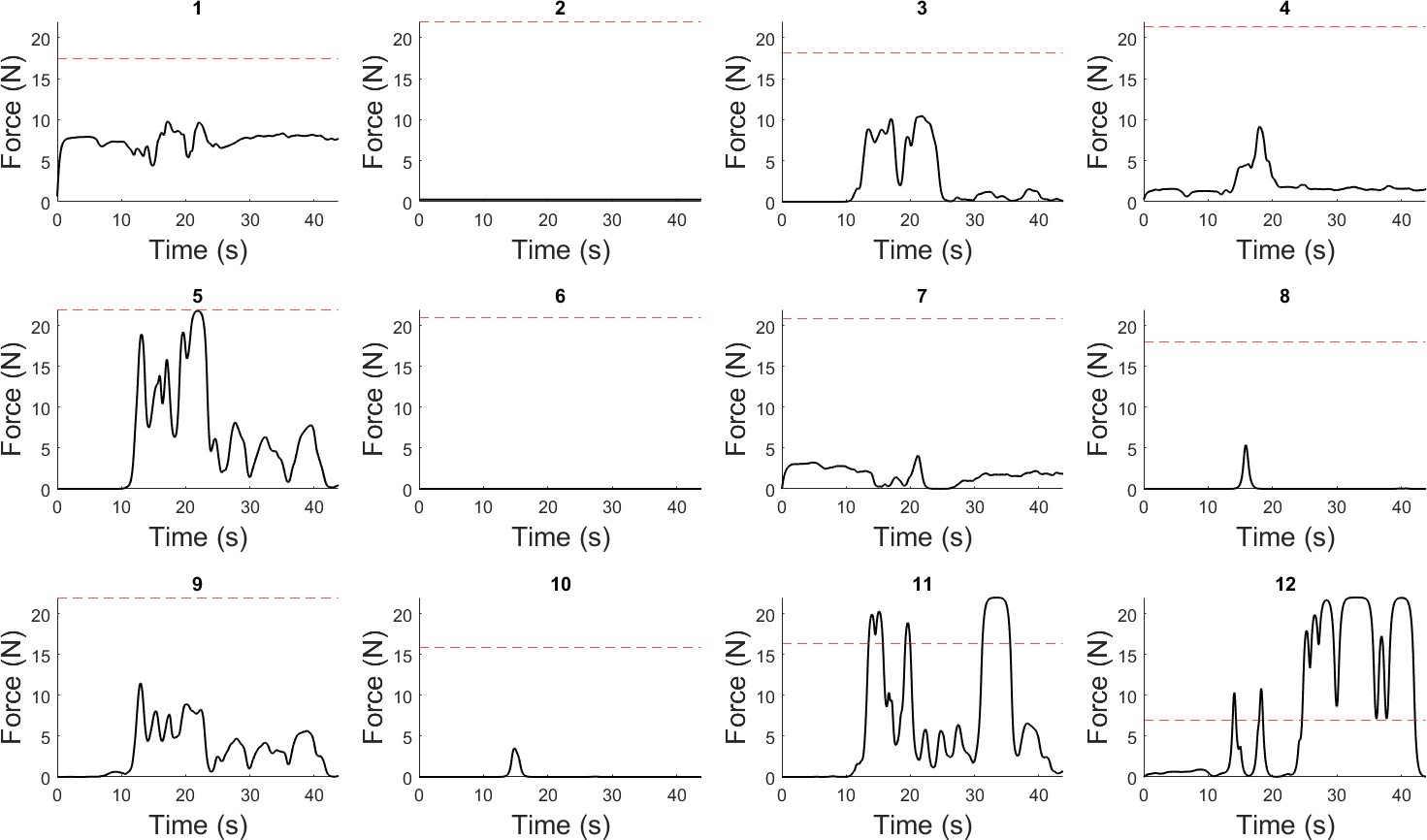


**Figure S4.** Force Sensor Readings for Participant 2 – Right Hand. The red dashed line represents the expert-defined reference benchmark.


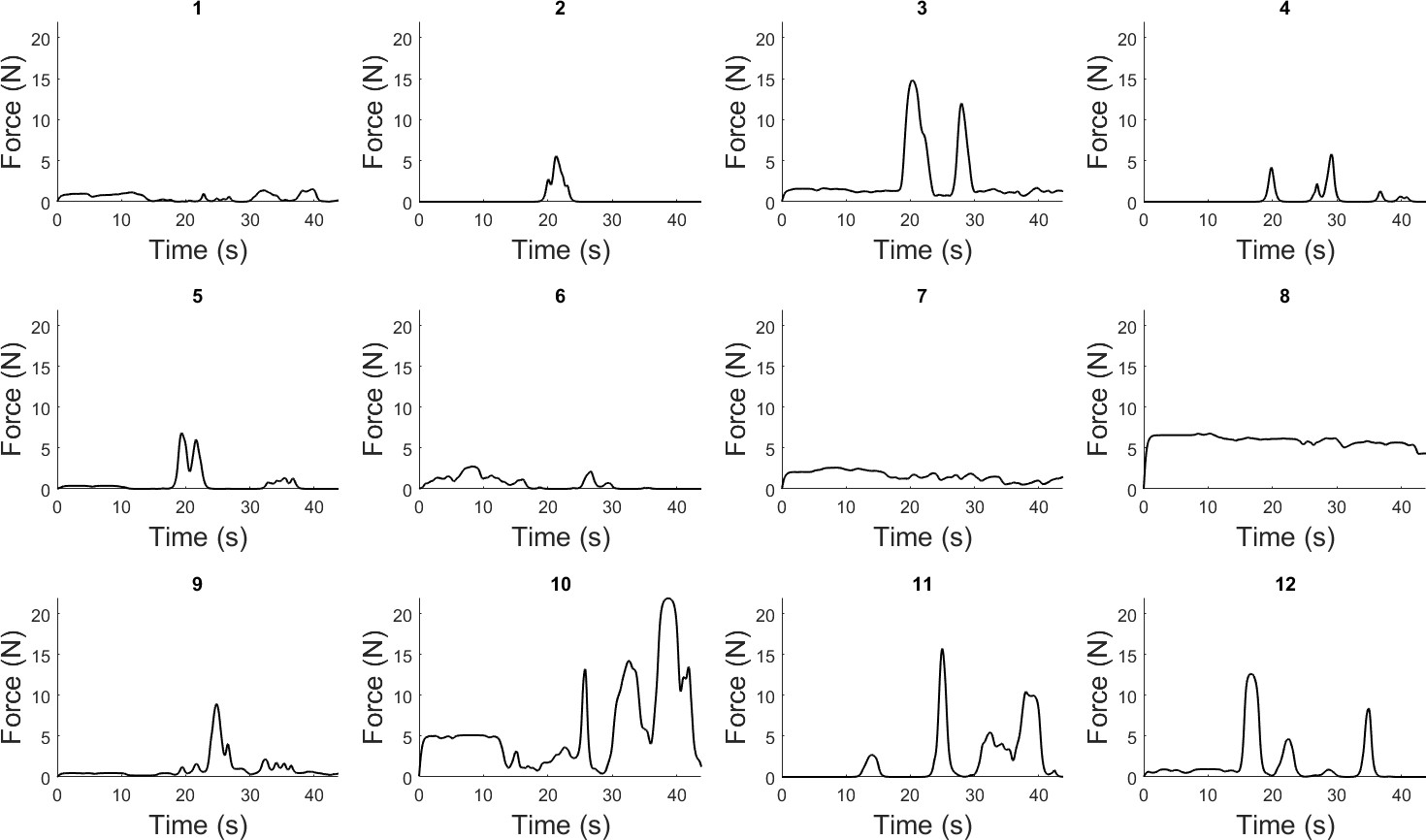


**Figure S5.** Force Sensor Readings for Participant 2 – Left Hand.


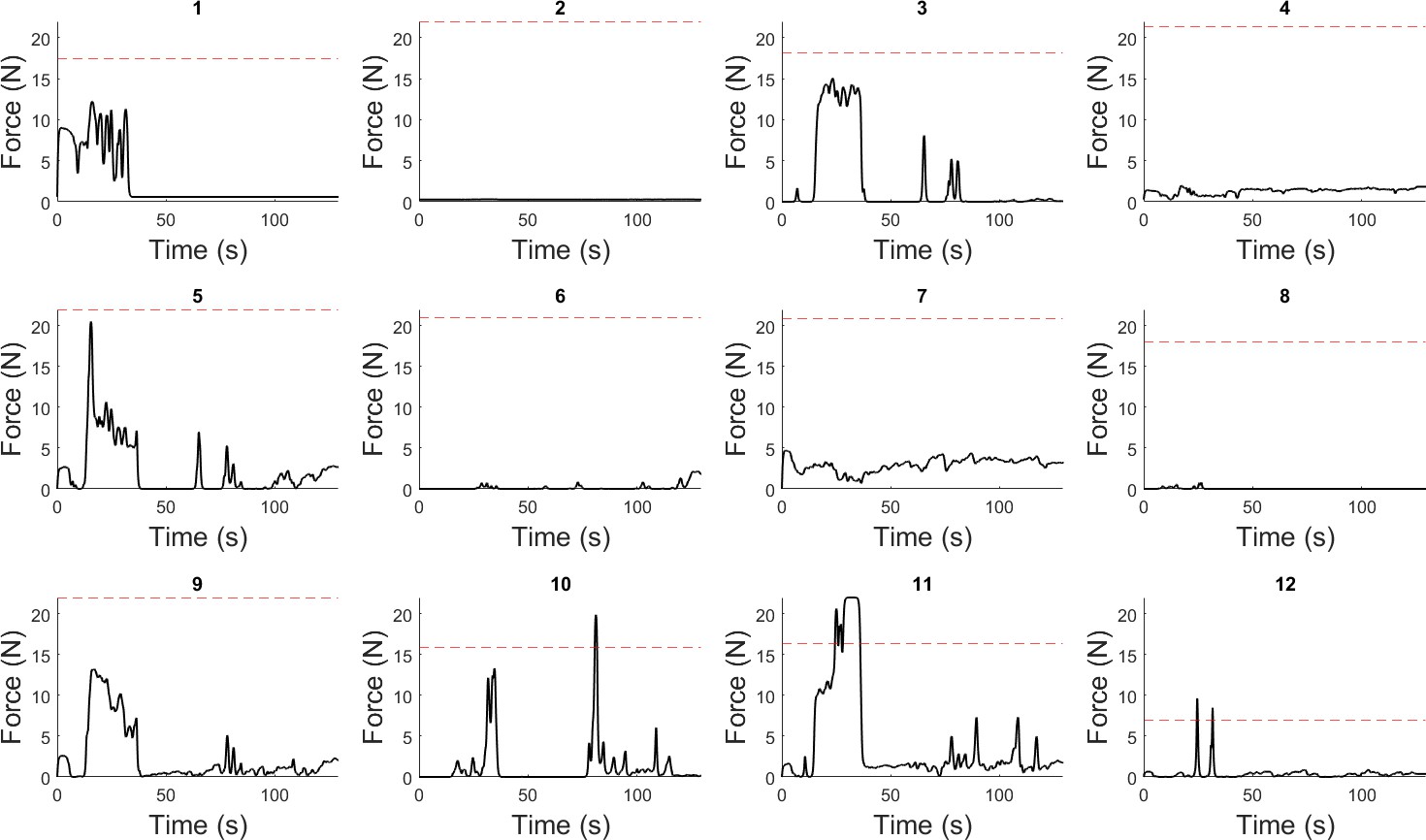


**Figure S6.** Force Sensor Readings for Participant 3 – Right Hand. The red dashed line represents the expert-defined reference benchmark.


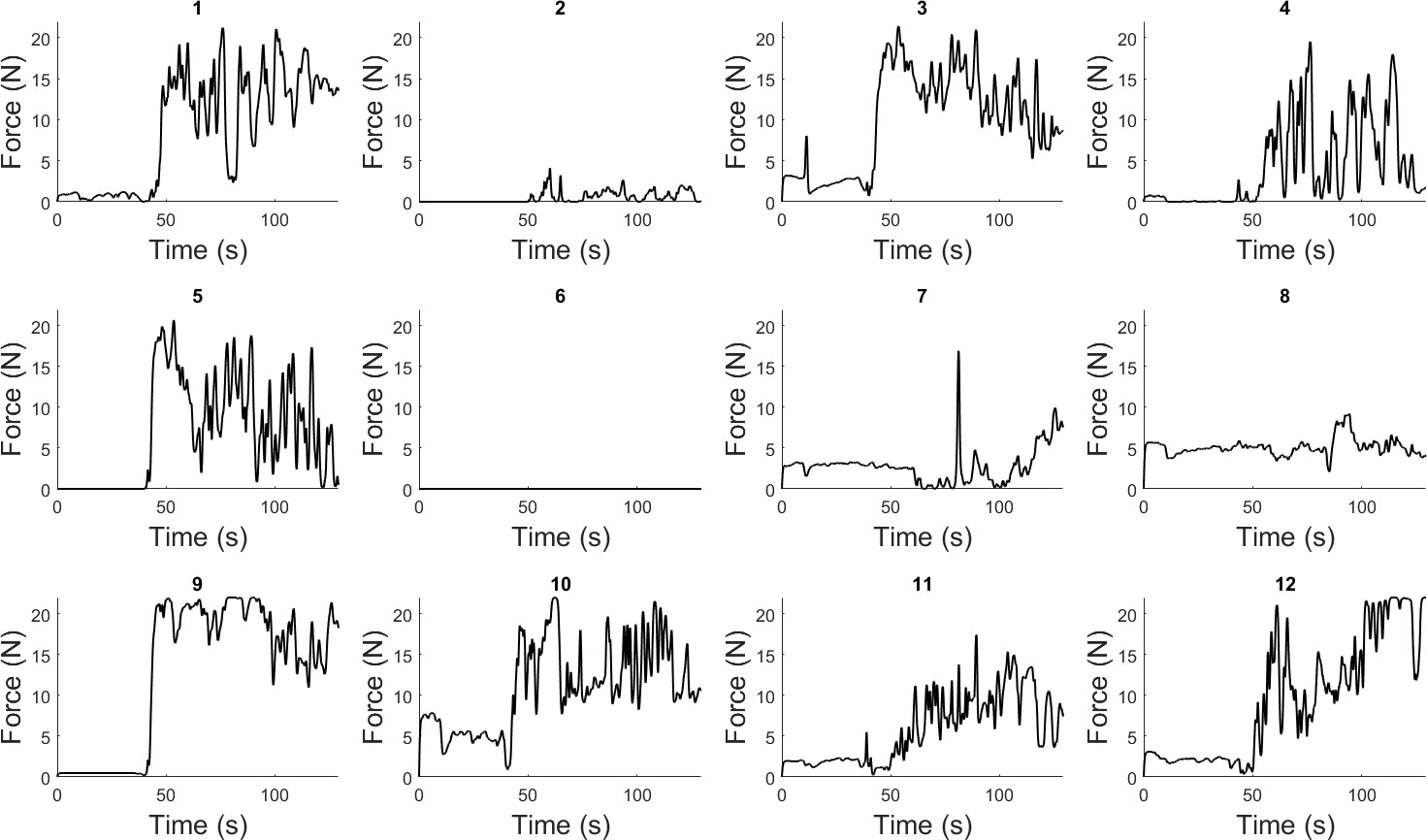


**Figure S7.** Force Sensor Readings for Participant 3 – Left Hand.


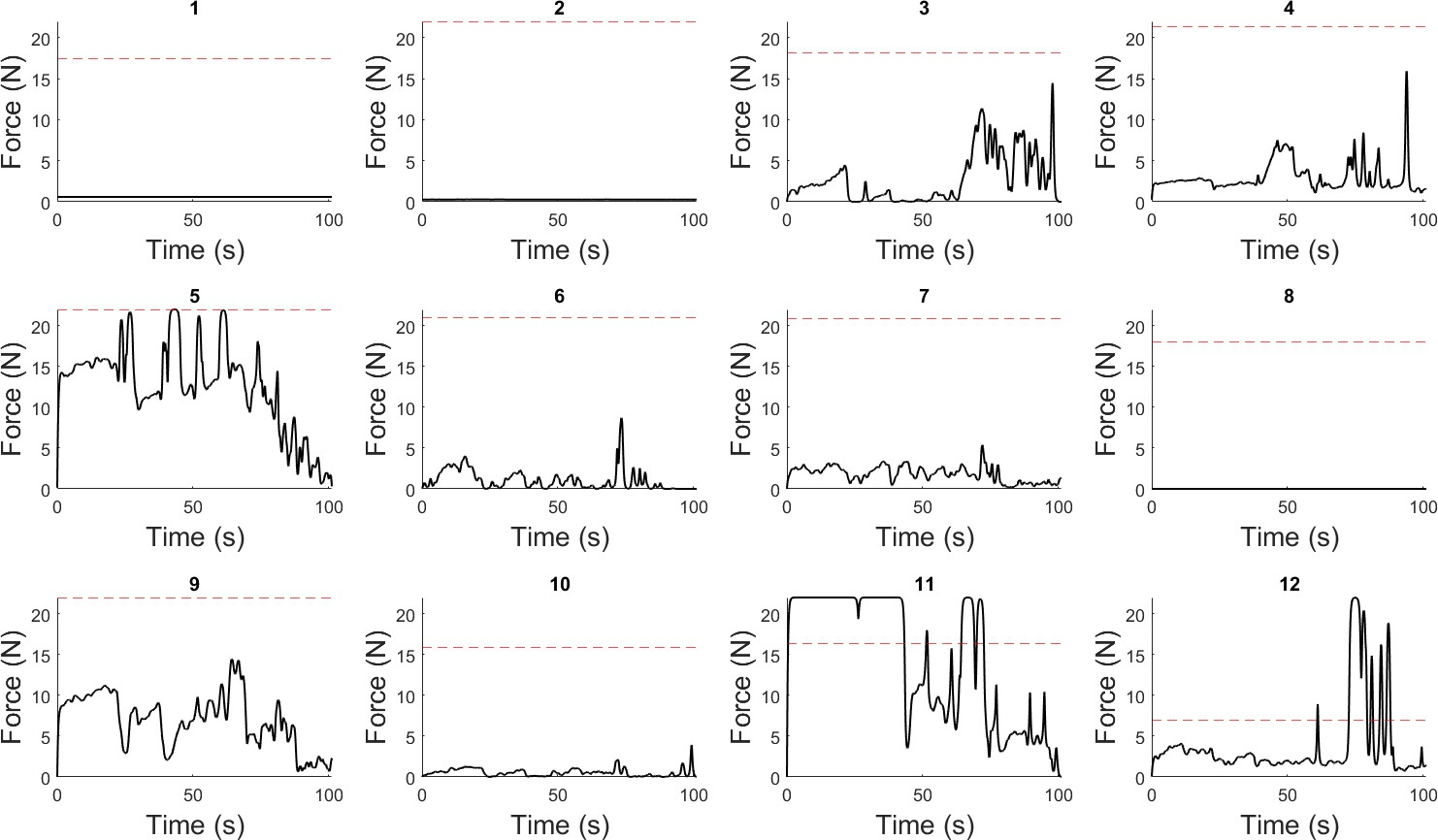


**Figure S8.** Force Sensor Readings for Participant 3 – Right Hand, assisted by participant 4. The red dashed line represents the expert-defined reference benchmark.


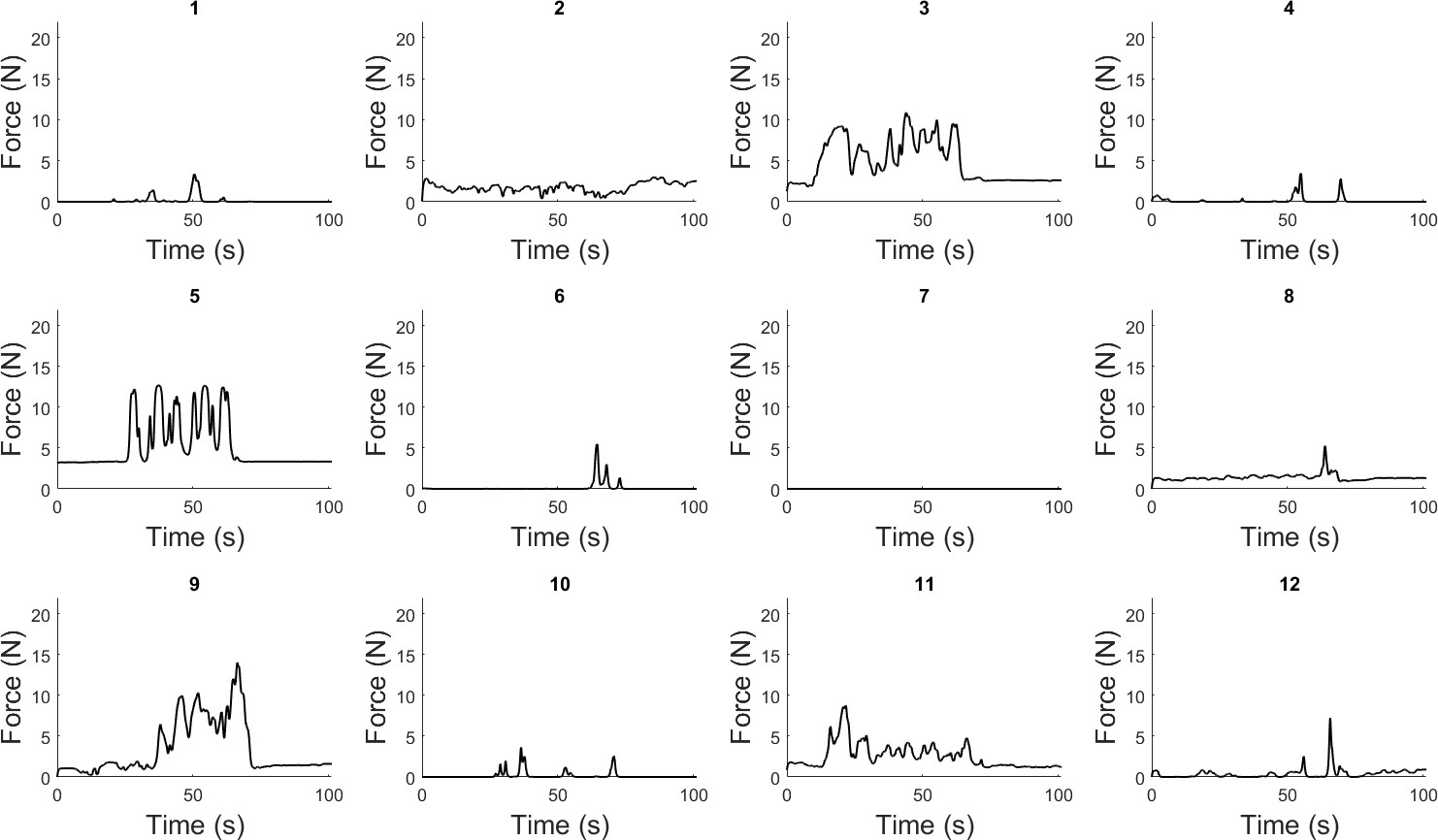


**Figure S9.** Force Sensor Readings for Participant 4– Right Hand, assisting Participant 3.


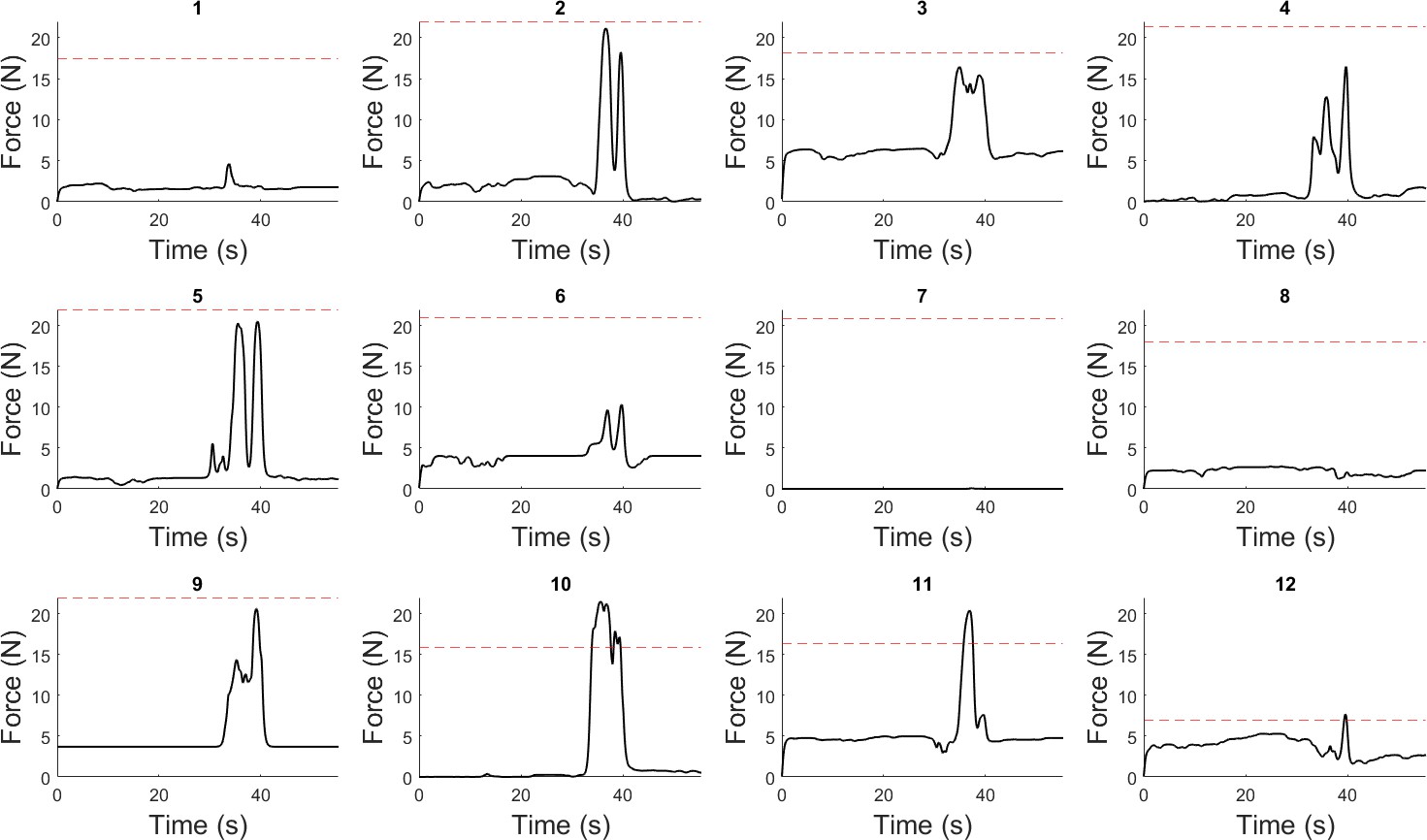


**Figure S10.** Force Sensor Readings for Participant 5 – Right Hand. The red dashed line represents the expert-defined reference benchmark.


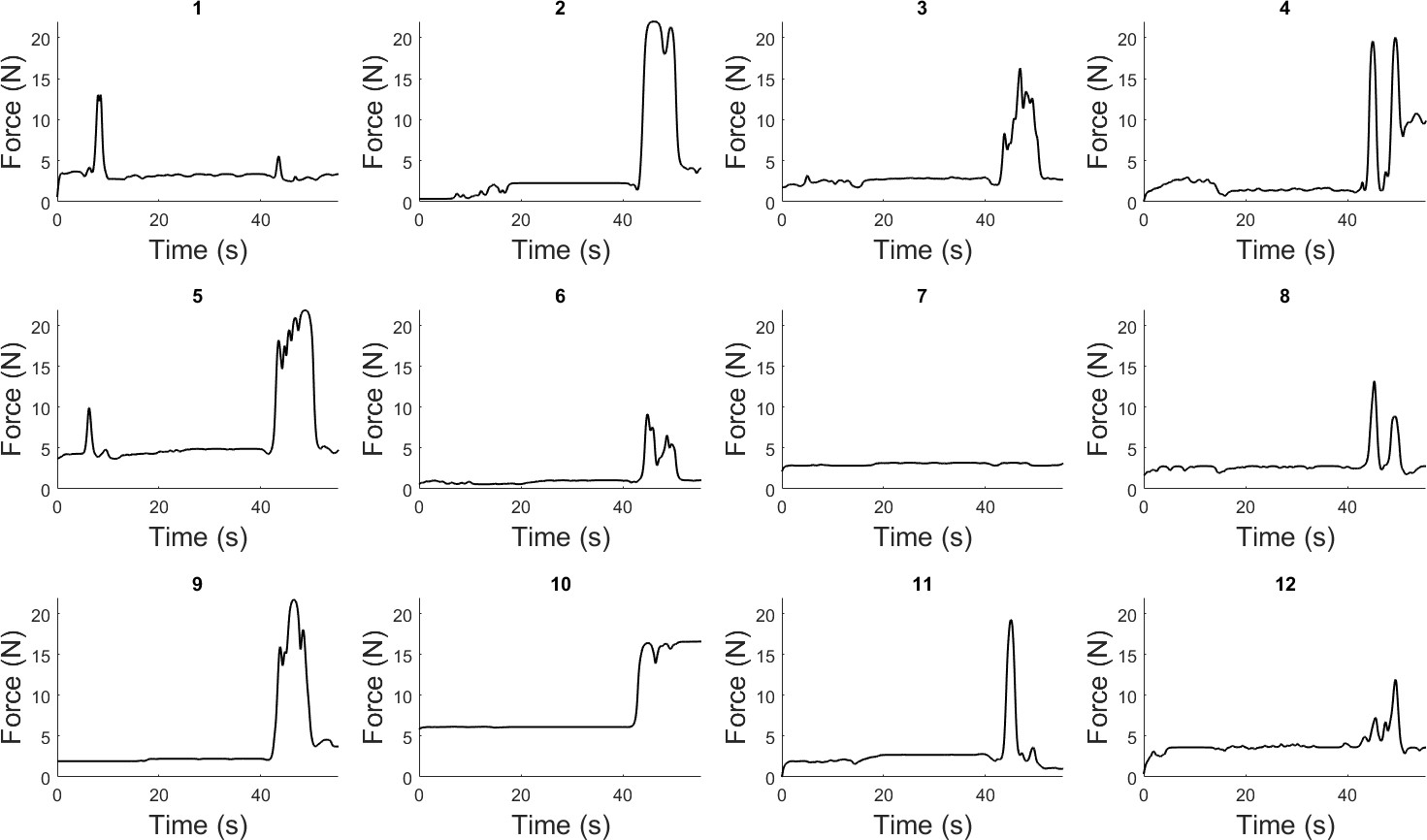


**Figure S11.** Force Sensor Readings for Participant 5 – Left Hand.


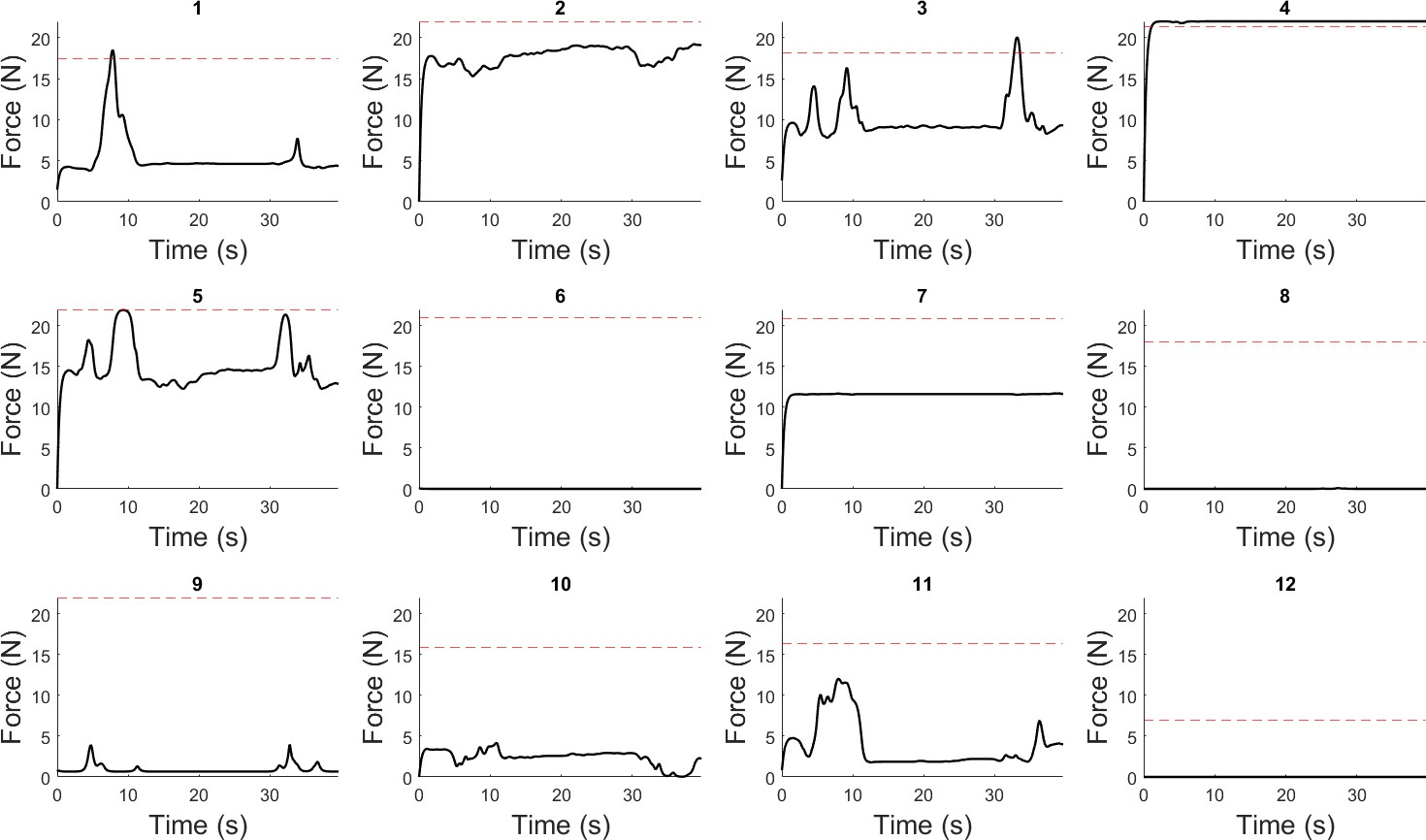


**Figure S12.** Force Sensor Readings for Participant 6 – Right Hand. The red dashed line represents the expert-defined reference benchmark.


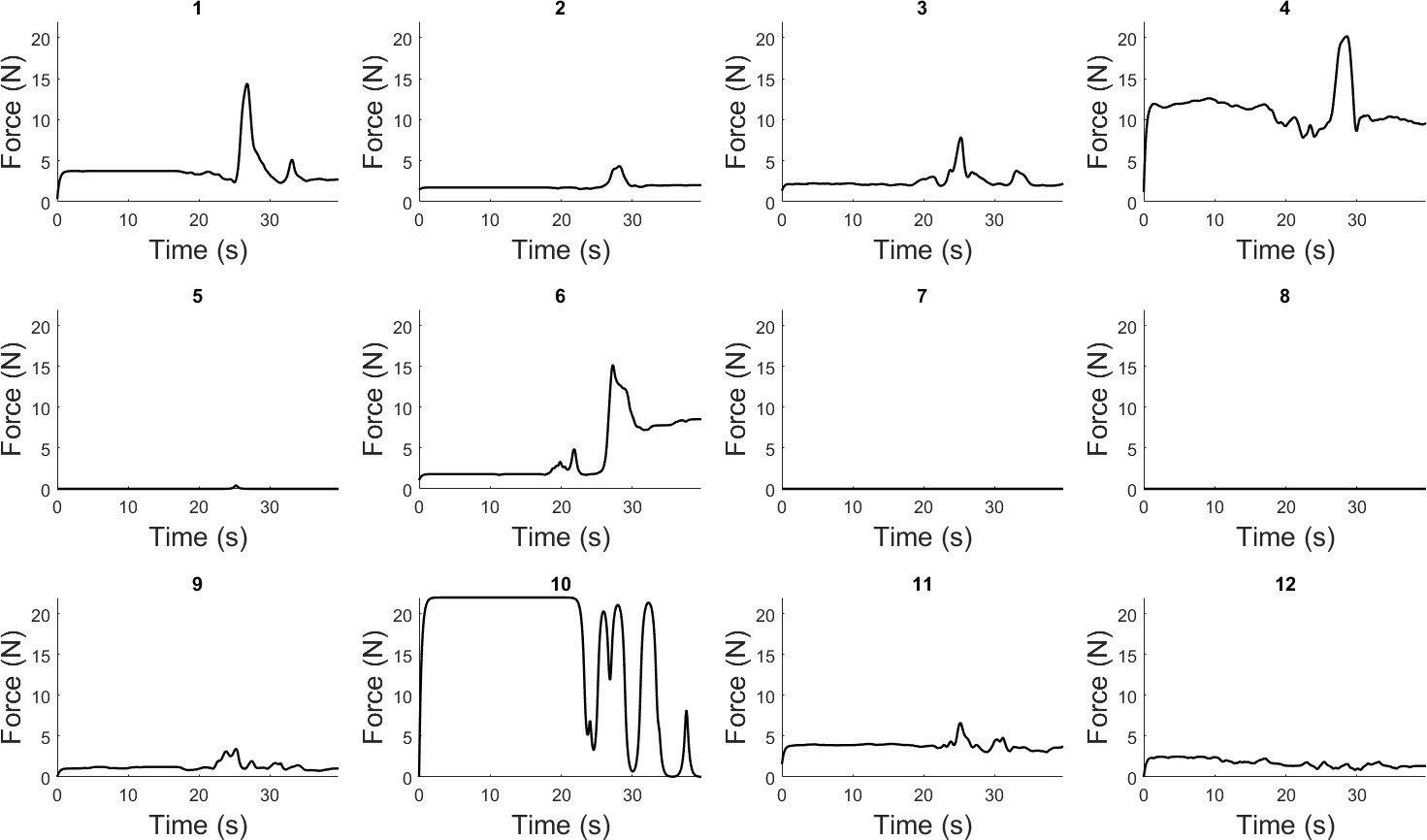


**Figure S13.** Force Sensor Readings for Participant 6 – Left Hand**.**


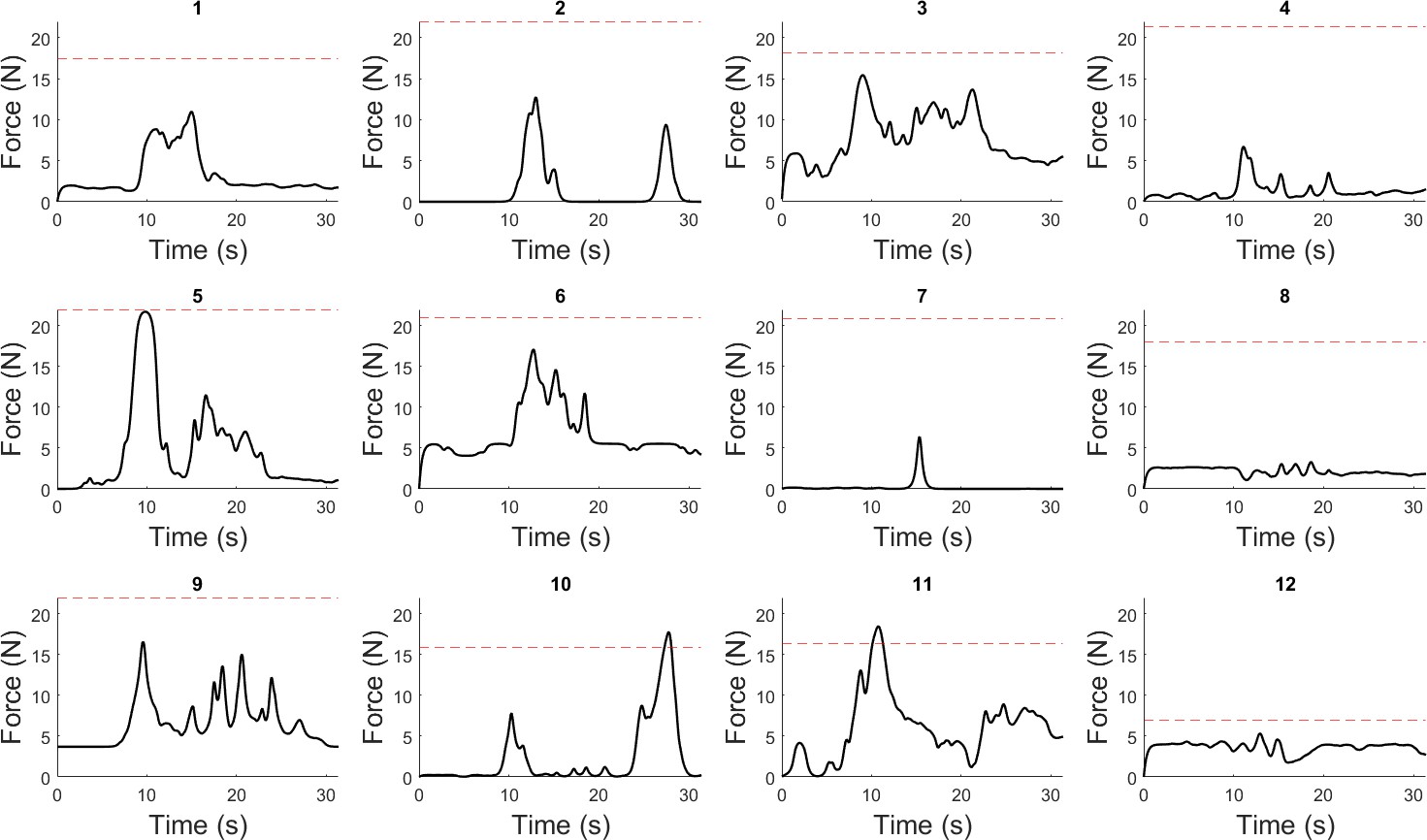


**Figure S14.** Force Sensor Readings for Participant 7 – Right Hand. The red dashed line represents the expert-defined reference benchmark.


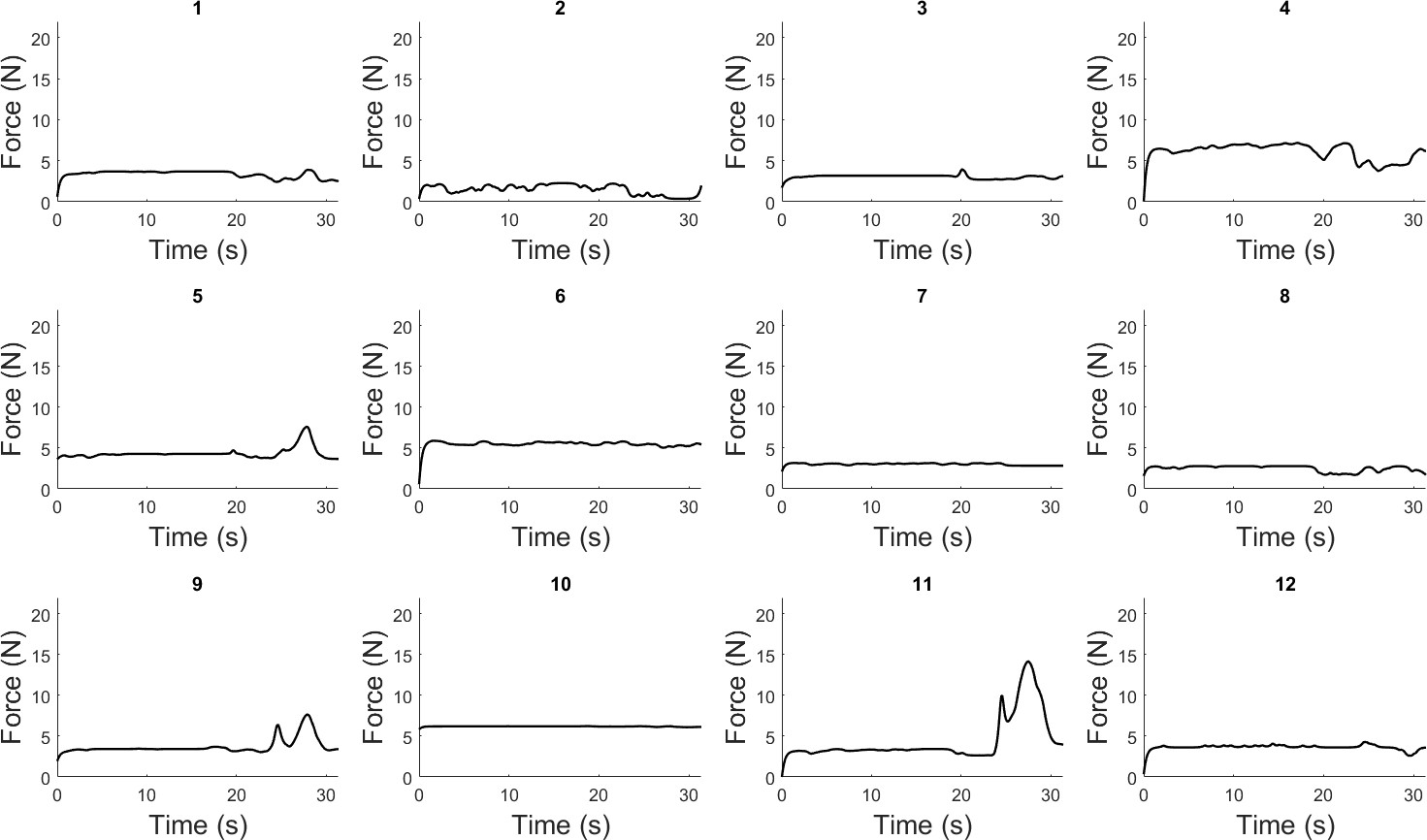


**Figure S15.** Force Sensor Readings for Participant 7 – Left Hand.


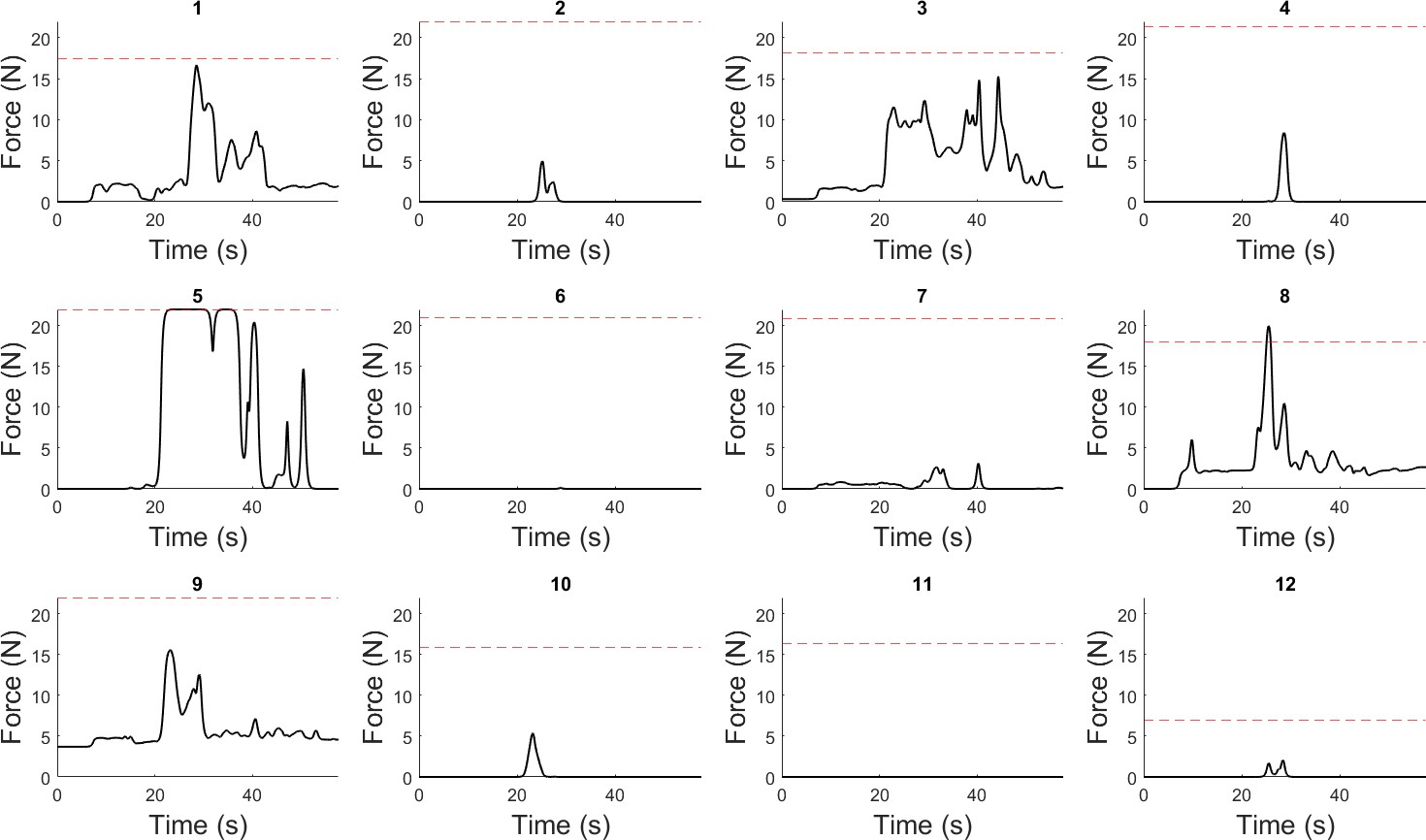


**Figure S16.** Force Sensor Readings for Participant 8 – Right Hand. The red dashed line represents the expert-defined reference benchmark.


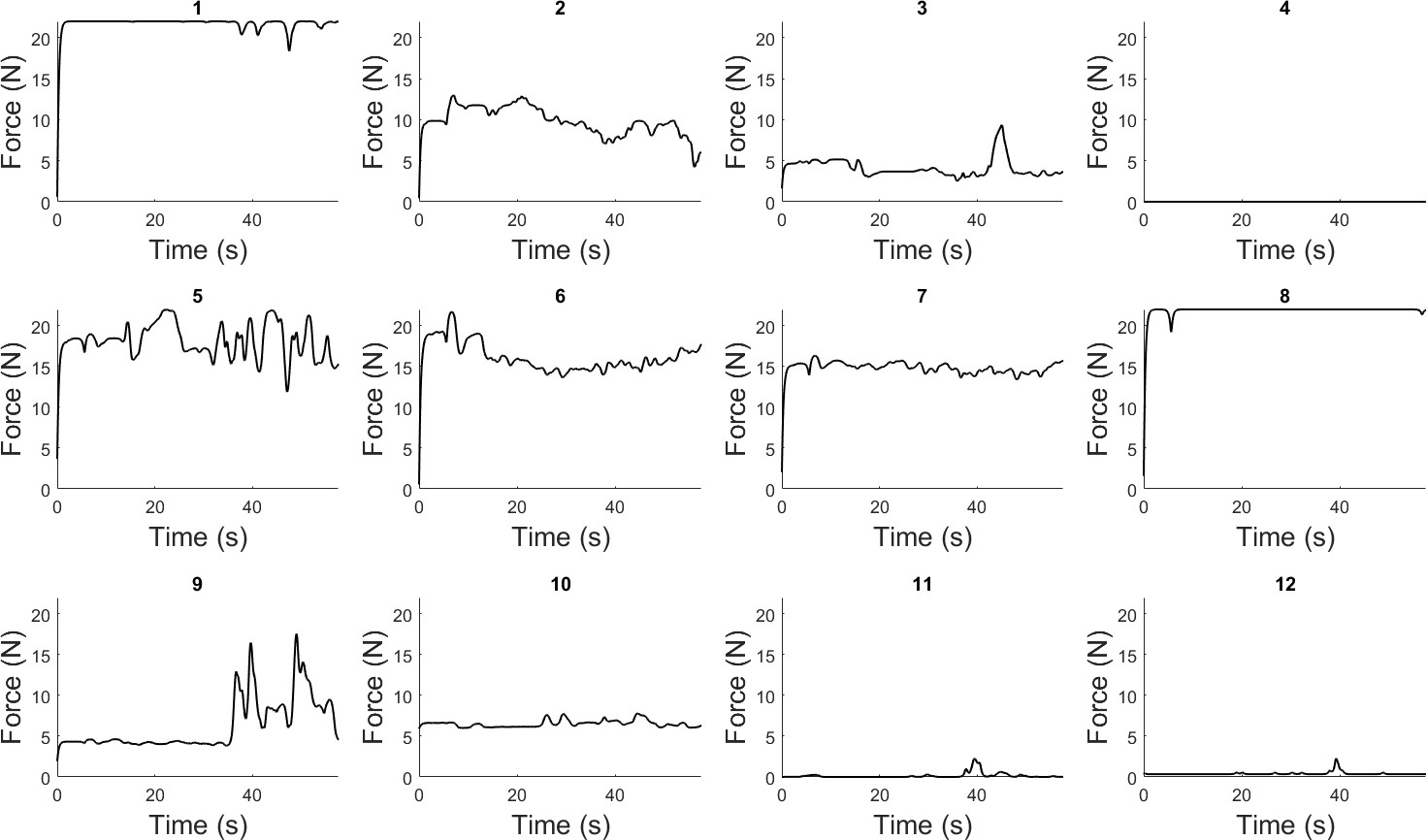


**Figure S17.** Force Sensor Readings for Participant 8 – Left Hand.


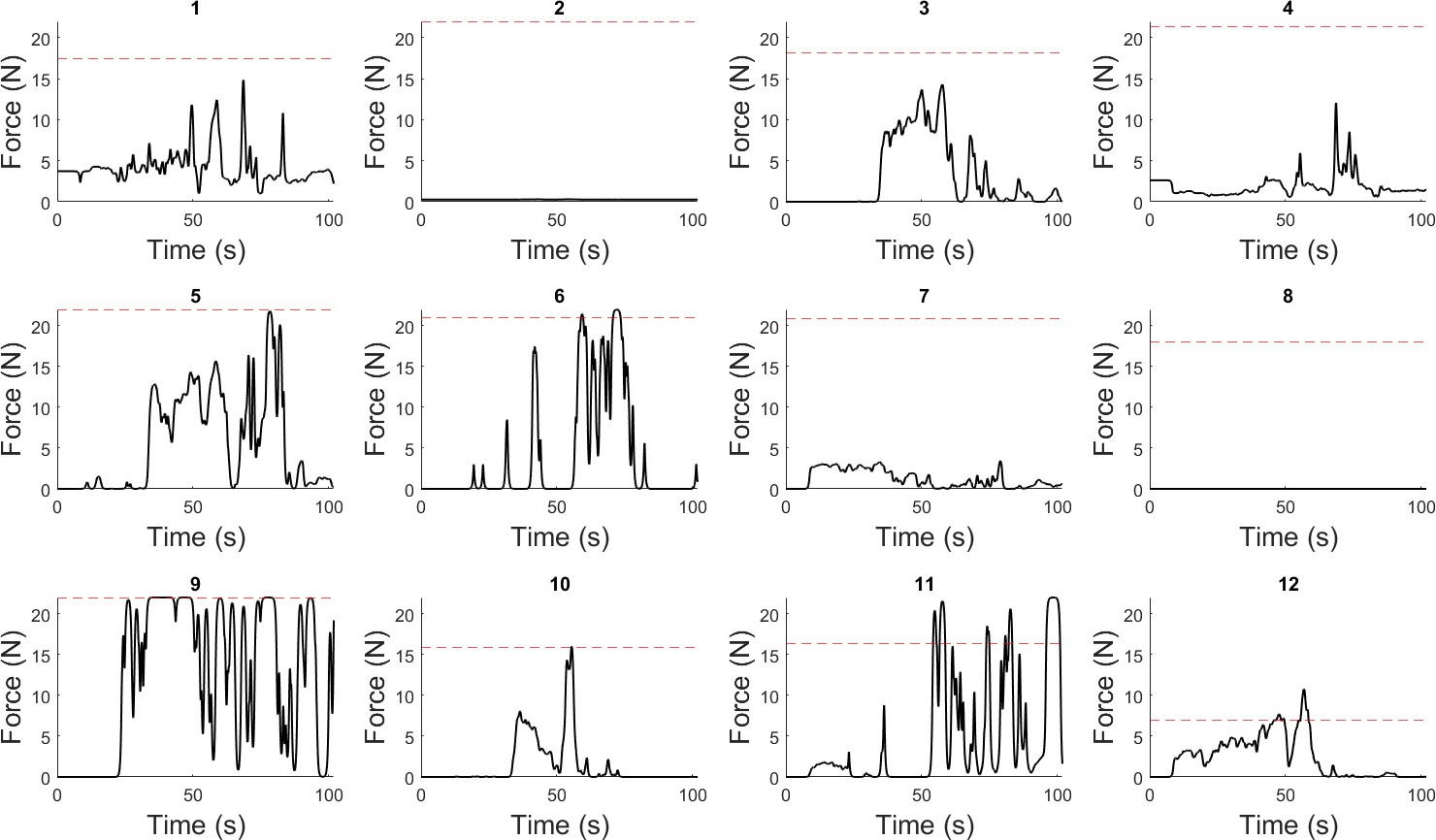


**Figure S18.** Force Sensor Readings for Participant 9 – Right Hand. The red dashed line represents the expert-defined reference benchmark.


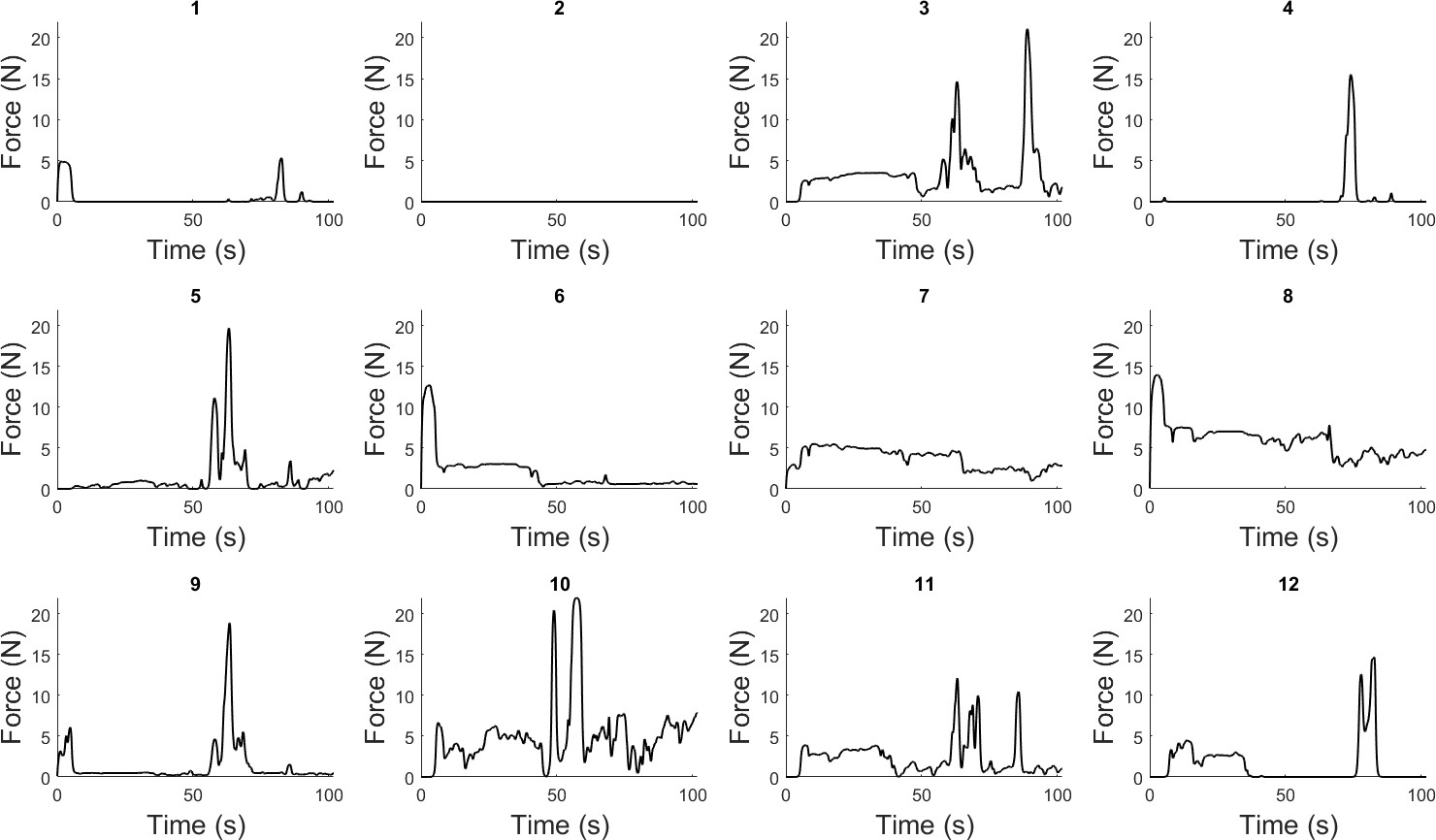


**Figure S19.** Force Sensor Readings for Participant 9 – Left Hand.


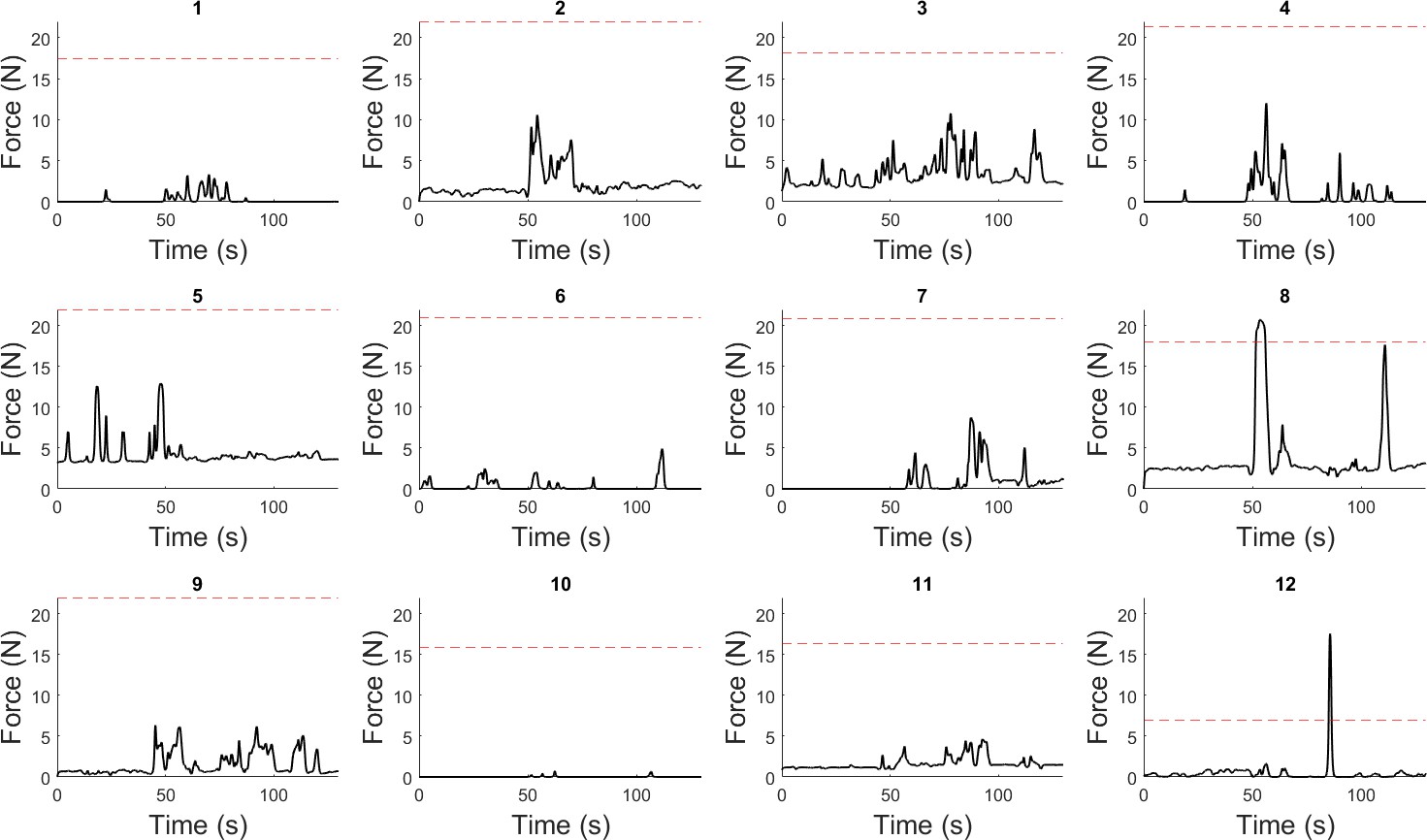


**Figure S20.** Force Sensor Readings for Participant 10– Right Hand. The red dashed line represents the expert-defined reference benchmark.


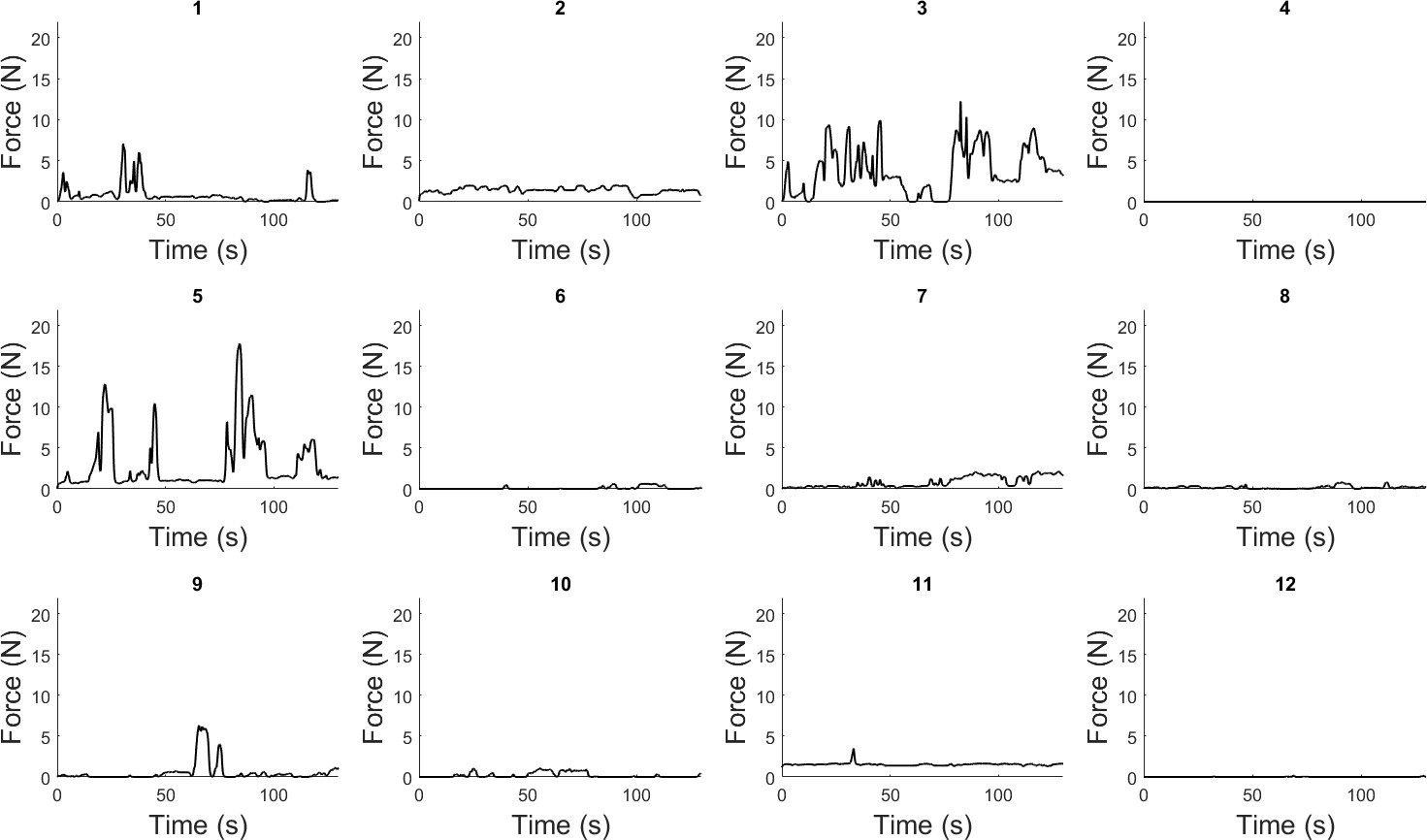


**Figure S21.** Force Sensor Readings for Participant 10– Left Hand.


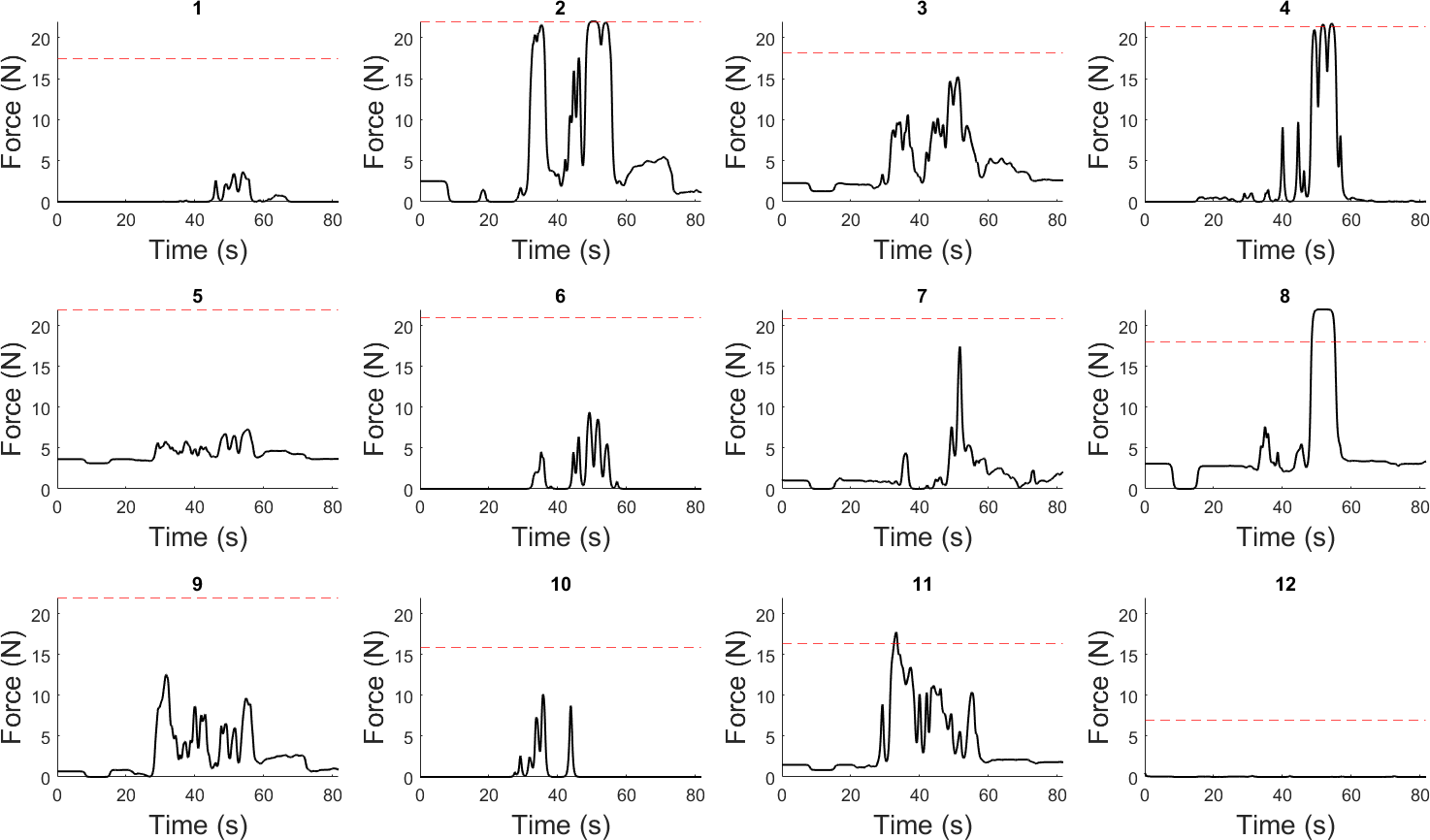


**Figure S22.** Force Sensor Readings for Participant 11– Right Hand. The red dashed line represents the expert-defined reference benchmark.


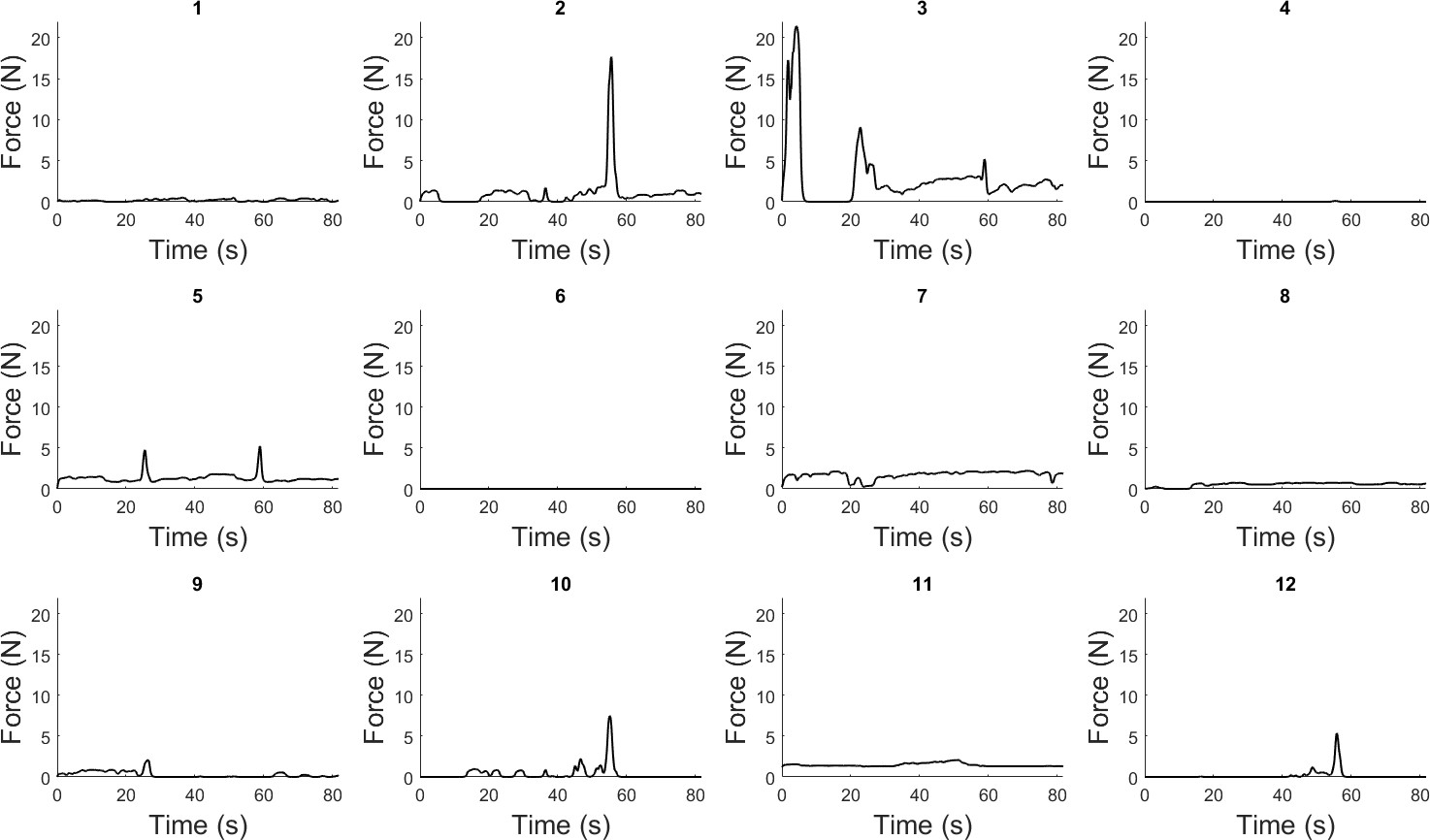


**Figure S23*.*** Force Sensor Readings for Participant 11– Left Hand.


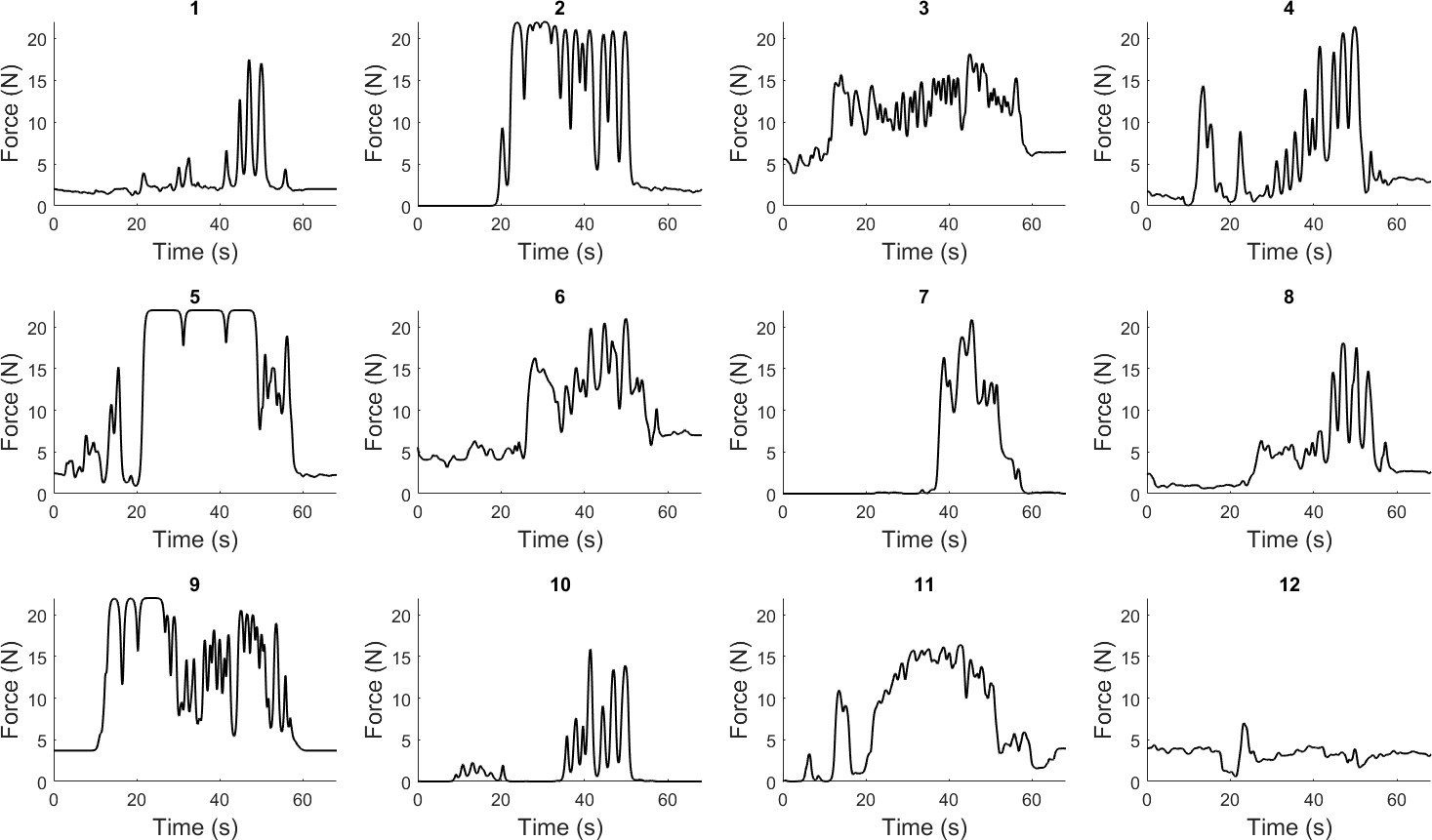


**Figure S24.** Force Sensor Readings for Participant 12– Right Hand.


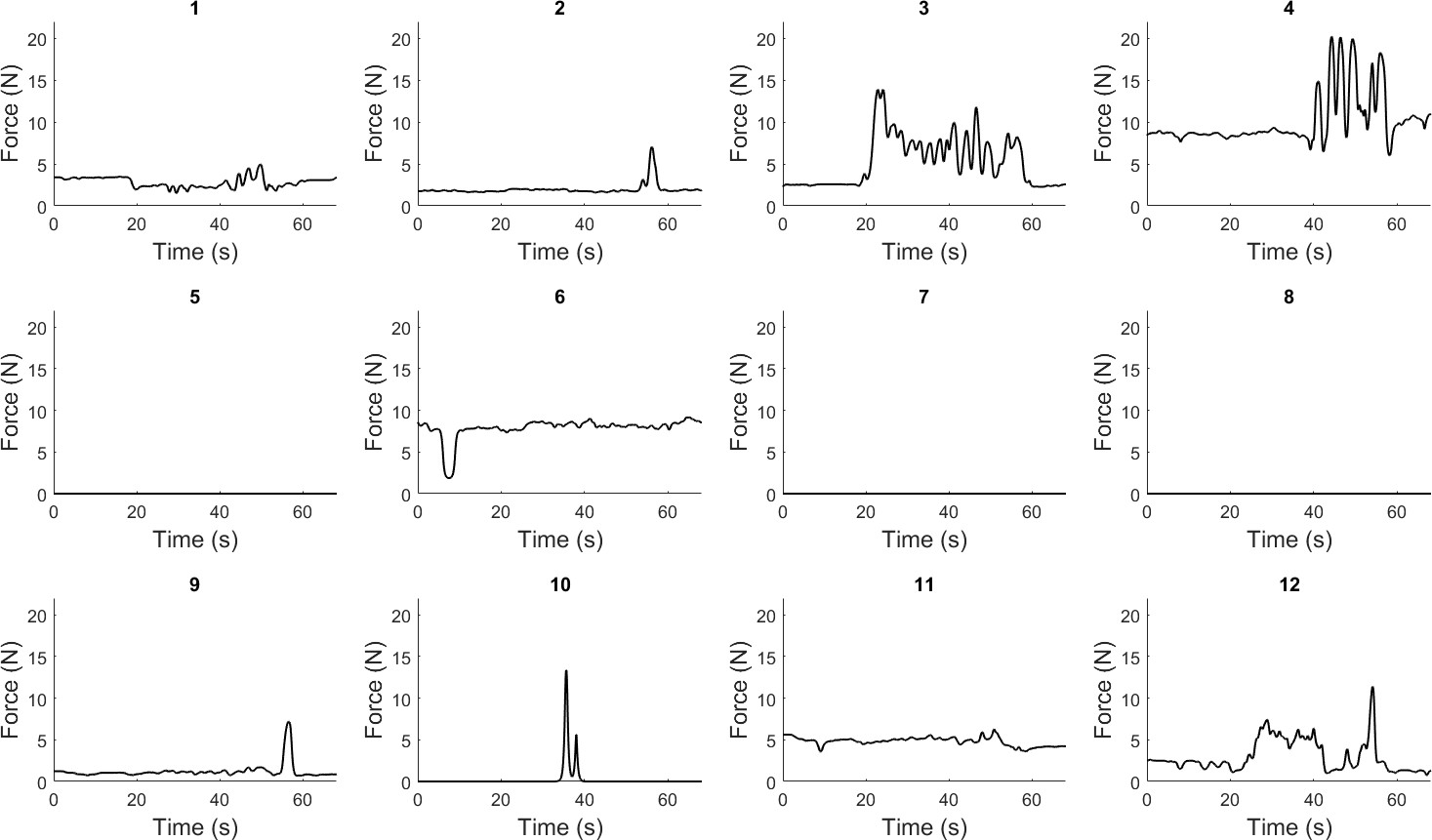


**Figure S25.** Force Sensor Readings for Participant 12– Left Hand.


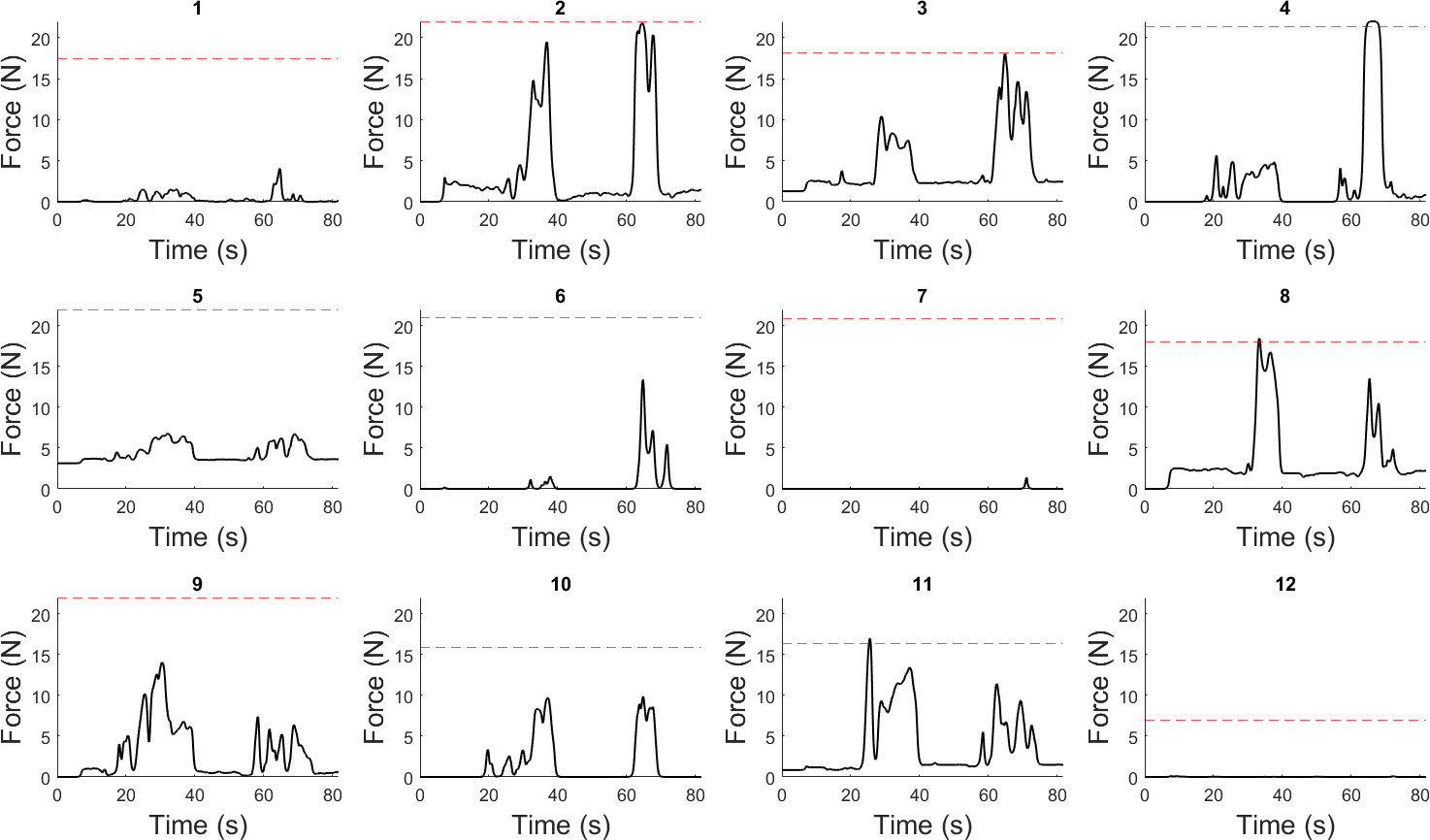


**Figure S26.** Force Sensor Readings for Participant 13– Right Hand. The red dashed line represents the expert-defined reference benchmark.


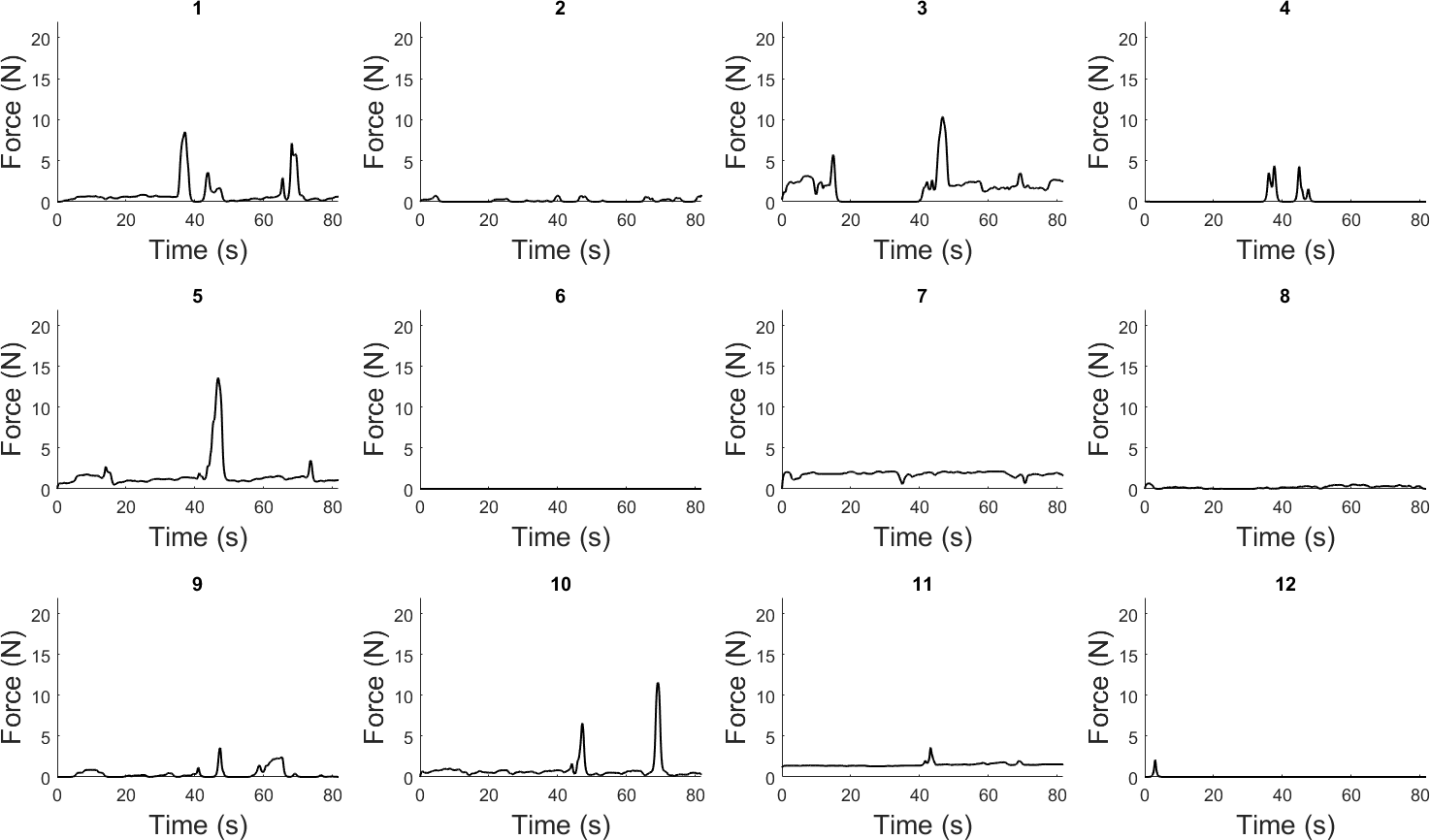


**Figure S27.** Force Sensor Readings for Participant 13– Left Hand.

| **Right Hand** | | | | | | | |
| --- | --- | --- | --- | --- | --- | --- | --- |
| **Sensor number (N)** | **Max (N)** | **Min (N)** | **Range (N)** | **Time-averaged (N)** | **Median (N)** | **Q1 (N)** | **Q3 (N)** |
| **S1** | 7.45 | 0.59 | 6.86 | 4.61 | 5.18 | 2.36 | 6.53 |
| **S2** | 0.27 | 0.26 | 0.01 | 0.26 | 0.26 | 0.26 | 0.26 |
| **S3** | 8.78 | 0 | 8.78 | 2.52 | 2.32 | 0.03 | 3.73 |
| **S4** | 7.67 | 0.27 | 7.4 | 2.42 | 1.97 | 1.71 | 2.63 |
| **S5** | 21.68 | 0 | 21.68 | 5.81 | 1.82 | 0.6 | 10.85 |
| **S6** | 1.98 | 0 | 1.98 | 0.24 | 0 | 0 | 0.13 |
| **S7** | 2.94 | 0 | 2.94 | 1.18 | 1.35 | 0.33 | 1.62 |
| **S8** | 0.1 | 0 | 0.1 | 0 | 0 | 0 | 0 |
| **S9** | 7.96 | 0 | 7.96 | 1.64 | 0.91 | 0.49 | 1.83 |
| **S10** | 0.71 | 0 | 0.71 | 0.02 | 0 | 0 | 0 |
| **S11** | 17.35 | 0 | 17.35 | 1.26 | 0 | 0 | 0.09 |
| **S12** | 13.32 | 0 | 13.32 | 0.77 | 0.37 | 0.09 | 0.56 |
| **Left Hand** | | | | | | | |
| **Sensor number** | **Max (N)** | **Min (N)** | **Range (N)** | **Time-averaged (N)** | **Median (N)** | **Q1 (N)** | **Q3 (N)** |
| **S1** | 1.31 | 0 | 1.31 | 0.05 | 0 | 0 | 0 |
| **S2** | 0 | 0 | 0 | 0 | 0 | 0 | 0 |
| **S3** | 8.92 | 0 | 8.92 | 1 | 0.02 | 0 | 0.99 |
| **S4** | 1.17 | 0 | 1.17 | 0.05 | 0 | 0 | 0 |
| **S5** | 4.38 | 0 | 4.38 | 0.32 | 0 | 0 | 0.12 |
| **S6** | 0 | 0 | 0 | 0 | 0 | 0 | 0 |
| **S7** | 2.82 | 0 | 2.82 | 2.2 | 2.28 | 1.91 | 2.59 |
| **S8** | 7.29 | 0 | 7.29 | 6.27 | 6.59 | 5.72 | 6.97 |
| **S9** | 0.18 | 0 | 0.18 | 0.17 | 0.18 | 0.18 | 0.18 |
| **S10** | 5.01 | 0 | 5.01 | 3.61 | 3.87 | 3.05 | 4.4 |
| **S11** | 0 | 0 | 0 | 0 | 0 | 0 | 0 |
| **S12** | 2.83 | 0 | 2.84 | 0.62 | 0.55 | 0.11 | 0.93 |

**Table S2.** Force data acquired from participant 1.

| **Right Hand** | | | | | | | |
| --- | --- | --- | --- | --- | --- | --- | --- |
| **Sensor number** | **Max (N)** | **Min (N)** | **Range (N)** | **Time-averaged (N)** | **Median (N)** | **Q1 (N)** | **Q3 (N)** |
| **S1** | 9.77 | 0.66 | 9.11 | 7.5 | 7.75 | 7.02 | 8.04 |
| **S2** | 0.27 | 0.26 | 0.01 | 0.26 | 0.26 | 0.26 | 0.26 |
| **S3** | 10.46 | 0 | 10.46 | 2.44 | 0.5 | 0.07 | 3.69 |
| **S4** | 9.17 | 0.27 | 8.9 | 2.02 | 1.55 | 1.41 | 1.79 |
| **S5** | 21.83 | 0 | 21.83 | 5.77 | 4.42 | 0.18 | 7.77 |
| **S6** | 0 | 0 | 0 | 0 | 0 | 0 | 0 |
| **S7** | 4.1 | 0 | 4.1 | 1.7 | 1.78 | 0.98 | 2.46 |
| **S8** | 5.39 | 0 | 5.39 | 0.13 | 0 | 0 | 0 |
| **S9** | 11.49 | 0 | 11.49 | 3.24 | 2.96 | 0.51 | 5.08 |
| **S10** | 3.54 | 0 | 3.54 | 0.12 | 0 | 0 | 0 |
| **S11** | 21.99 | 0 | 21.99 | 6.06 | 3.06 | 0.37 | 7.66 |
| **S12** | 22 | 0 | 22 | 8.11 | 2.03 | 0.47 | 18.25 |
| **Left Hand** | | | | | | | |
| **Sensor number** | **Max (N)** | **Min (N)** | **Range (N)** | **Time-averaged (N)** | **Median (N)** | **Q1 (N)** | **Q3 (N)** |
| **S1** | 1.54 | 0 | 1.54 | 0.54 | 0.47 | 0.12 | 0.91 |
| **S2** | 5.57 | 0 | 5.57 | 0.28 | 0 | 0 | 0 |
| **S3** | 14.8 | 0 | 14.8 | 2.58 | 1.35 | 1.21 | 1.59 |
| **S4** | 5.8 | 0 | 5.8 | 0.37 | 0 | 0 | 0.19 |
| **S5** | 6.86 | 0 | 6.86 | 0.63 | 0.12 | 0 | 0.37 |
| **S6** | 2.74 | 0 | 2.74 | 0.62 | 0.18 | 0 | 1.18 |
| **S7** | 2.61 | 0 | 2.61 | 1.6 | 1.5 | 1.23 | 2.09 |
| **S8** | 6.78 | 0 | 6.78 | 5.94 | 6.08 | 5.65 | 6.29 |
| **S9** | 8.96 | 0 | 8.96 | 0.98 | 0.47 | 0.4 | 0.99 |
| **S10** | 21.9 | 0.08 | 21.82 | 6.06 | 4.87 | 2.31 | 7.09 |
| **S11** | 15.75 | 0 | 15.75 | 1.97 | 0.18 | 0 | 2.77 |
| **S12** | 12.62 | 0 | 12.62 | 1.48 | 0.63 | 0.08 | 0.95 |

**Table S3.** Force data acquired from participant 2.

| **Right Hand** | | | | | | | |
| --- | --- | --- | --- | --- | --- | --- | --- |
| **Sensor number** | **Max (N)** | **Min (N)** | **Range (N)** | **Time-averaged (N)** | **Median (N)** | **Q1 (N)** | **Q3 (N)** |
| **S1** | 12.23 | 0.59 | 11.64 | 2.45 | 0.59 | 0.59 | 2.67 |
| **S2** | 0.3 | 0.26 | 0.04 | 0.27 | 0.27 | 0.26 | 0.28 |
| **S3** | 15.06 | 0 | 15.06 | 2.42 | 0.05 | 0 | 0.67 |
| **S4** | 1.98 | 0.28 | 1.7 | 1.32 | 1.44 | 1.21 | 1.56 |
| **S5** | 20.51 | 0 | 20.51 | 2.21 | 0.74 | 0 | 2.67 |
| **S6** | 2.16 | 0 | 2.16 | 0.17 | 0 | 0 | 0.07 |
| **S7** | 4.71 | 0 | 4.71 | 2.96 | 3.1 | 2.52 | 3.55 |
| **S8** | 0.79 | 0 | 0.79 | 0.03 | 0 | 0 | 0 |
| **S9** | 13.21 | 0 | 13.21 | 2.39 | 0.82 | 0.42 | 2.15 |
| **S10** | 19.87 | 0 | 19.87 | 1.15 | 0.18 | 0 | 0.86 |
| **S11** | 22 | 0 | 22 | 3.99 | 1.48 | 1.17 | 2.54 |
| **S12** | 9.64 | 0 | 9.64 | 0.41 | 0.32 | 0.07 | 0.51 |
| **Left Hand** | | | | | | | |
| **Sensor number** | **Max (N)** | **Min (N)** | **Range (N)** | **Time-averaged (N)** | **Median (N)** | **Q1 (N)** | **Q3 (N)** |
| **S1** | 21.23 | 0.01 | 21.22 | 8.76 | 10.87 | 0.93 | 14.55 |
| **S2** | 4.12 | 0 | 4.12 | 0.5 | 0.08 | 0 | 0.83 |
| **S3** | 21.41 | 0.01 | 21.39 | 9.82 | 10.41 | 2.92 | 14.97 |
| **S4** | 19.52 | 0 | 19.52 | 4.21 | 1.66 | 0.09 | 7.5 |
| **S5** | 20.68 | 0 | 20.68 | 6.7 | 6.11 | 0 | 12.13 |
| **S6** | 0 | 0 | 0 | 0 | 0 | 0 | 0 |
| **S7** | 16.92 | 0 | 16.92 | 2.77 | 2.76 | 1.3 | 3.14 |
| **S8** | 9.21 | 0 | 9.21 | 5.1 | 4.99 | 4.57 | 5.44 |
| **S9** | 22 | 0 | 22 | 12.67 | 16.85 | 0.47 | 20.55 |
| **S10** | 21.98 | 0.02 | 21.96 | 10.88 | 10.33 | 5.63 | 15.03 |
| **S11** | 17.43 | 0 | 17.43 | 5.79 | 4.66 | 1.98 | 9.25 |
| **S12** | 22 | 0 | 22 | 9.47 | 8.97 | 2.23 | 14.92 |

**Table S4.** Force data acquired from participant 3.

| **Participant 3 Right Hand** | | | | | | | |
| --- | --- | --- | --- | --- | --- | --- | --- |
| **Sensor number** | **Max (N)** | **Min (N)** | **Range (N)** | **Time-averaged (N)** | **Median (N)** | **Q1 (N)** | **Q3 (N)** |
| **S1** | 0.59 | 0.59 | 0 | 0.59 | 0.59 | 0.59 | 0.59 |
| **S2** | 0.29 | 0.26 | 0.03 | 0.27 | 0.27 | 0.27 | 0.27 |
| **S3** | 14.48 | 0 | 14.48 | 2.73 | 1.74 | 0.29 | 4.19 |
| **S4** | 15.94 | 0.26 | 15.68 | 2.99 | 2.36 | 1.94 | 3.12 |
| **S5** | 21.99 | 0.15 | 21.84 | 12.5 | 13.25 | 10.46 | 15.42 |
| **S6** | 8.7 | 0 | 8.7 | 1.04 | 0.56 | 0.15 | 1.53 |
| **S7** | 5.38 | 0.01 | 5.38 | 1.81 | 1.84 | 1.01 | 2.51 |
| **S8** | 0 | 0 | 0 | 0 | 0 | 0 | 0 |
| **S9** | 14.45 | 0.01 | 14.44 | 6.96 | 7.17 | 4.74 | 9.4 |
| **S10** | 3.92 | 0 | 3.92 | 0.54 | 0.48 | 0.13 | 0.79 |
| **S11** | 22 | 0 | 22 | 14.29 | 16.83 | 5.68 | 22 |
| **S12** | 21.98 | 0.05 | 21.93 | 3.82 | 2.14 | 1.68 | 3.3 |
| **Participant 4 Right Hand** | | | | | | | |
| **Sensor number** | **Max (N)** | **Min (N)** | **Range (N)** | **Time-averaged (N)** | **Median (N)** | **Q1 (N)** | **Q3 (N)** |
| **S1** | 21.23 | 0.01 | 21.22 | 8.76 | 10.87 | 0.93 | 14.55 |
| **S2** | 4.12 | 0 | 4.12 | 0.5 | 0.08 | 0 | 0.83 |
| **S3** | 21.41 | 0.01 | 21.39 | 9.82 | 10.41 | 2.92 | 14.97 |
| **S4** | 19.52 | 0 | 19.52 | 4.21 | 1.66 | 0.09 | 7.5 |
| **S5** | 20.68 | 0 | 20.68 | 6.7 | 6.11 | 0 | 12.13 |
| **S6** | 0 | 0 | 0 | 0 | 0 | 0 | 0 |
| **S7** | 16.92 | 0 | 16.92 | 2.77 | 2.76 | 1.3 | 3.14 |
| **S8** | 9.21 | 0 | 9.21 | 5.1 | 4.99 | 4.57 | 5.44 |
| **S9** | 22 | 0 | 22 | 12.67 | 16.85 | 0.47 | 20.55 |
| **S10** | 21.98 | 0.02 | 21.96 | 10.88 | 10.33 | 5.63 | 15.03 |
| **S11** | 17.43 | 0 | 17.43 | 5.79 | 4.66 | 1.98 | 9.25 |
| **S12** | 22 | 0 | 22 | 9.47 | 8.97 | 2.23 | 14.92 |

**Table S5.** Force data acquired from participant 3, right hand, assisted by participant 4, right hand.

| **Right Hand** | | | | | | | |
| --- | --- | --- | --- | --- | --- | --- | --- |
| **Sensor number** | **Max (N)** | **Min (N)** | **Range (N)** | **Time-averaged (N)** | **Median (N)** | **Q1 (N)** | **Q3 (N)** |
| **S1** | 4.64 | 0 | 4.64 | 1.77 | 1.72 | 1.54 | 1.87 |
| **S2** | 21.15 | 0 | 21.15 | 2.84 | 2.04 | 0.5 | 2.75 |
| **S3** | 16.45 | 0.34 | 16.12 | 6.98 | 6.07 | 5.65 | 6.37 |
| **S4** | 16.49 | 0 | 16.48 | 1.76 | 0.75 | 0.31 | 1.06 |
| **S5** | 20.54 | 0.01 | 20.53 | 2.85 | 1.34 | 1.2 | 1.5 |
| **S6** | 10.32 | 0.13 | 10.19 | 4.12 | 4.05 | 3.65 | 4.05 |
| **S7** | 0.09 | 0 | 0.09 | 0 | 0 | 0 | 0 |
| **S8** | 2.76 | 0 | 2.76 | 2.18 | 2.25 | 1.76 | 2.6 |
| **S9** | 20.62 | 3.7 | 16.92 | 5.05 | 3.7 | 3.7 | 3.7 |
| **S10** | 21.49 | 0 | 21.49 | 2.47 | 0.26 | 0.01 | 0.76 |
| **S11** | 20.41 | 0.03 | 20.38 | 5.36 | 4.7 | 4.55 | 4.91 |
| **S12** | 7.7 | 0.05 | 7.65 | 3.7 | 3.87 | 2.63 | 4.63 |
| **Left Hand** | | | | | | | |
| **Sensor number** | **Max (N)** | **Min (N)** | **Range (N)** | **Time-averaged (N)** | **Median (N)** | **Q1 (N)** | **Q3 (N)** |
| **S1** | 13.06 | 0.58 | 12.48 | 3.43 | 3.23 | 3.03 | 3.37 |
| **S2** | 21.97 | 0.36 | 21.61 | 4.14 | 2.27 | 1.21 | 2.27 |
| **S3** | 16.3 | 1.73 | 14.57 | 3.65 | 2.8 | 2.51 | 2.88 |
| **S4** | 20.03 | 0 | 20.03 | 3.41 | 1.6 | 1.37 | 2.66 |
| **S5** | 21.9 | 3.69 | 18.21 | 6.68 | 4.82 | 4.3 | 4.9 |
| **S6** | 9.15 | 0.54 | 8.62 | 1.43 | 0.99 | 0.7 | 1.05 |
| **S7** | 3.2 | 2.13 | 1.07 | 3.02 | 3.09 | 2.85 | 3.17 |
| **S8** | 13.23 | 1.66 | 11.56 | 3.03 | 2.71 | 2.52 | 2.78 |
| **S9** | 21.72 | 1.93 | 19.79 | 4.07 | 2.23 | 1.93 | 2.23 |
| **S10** | 16.61 | 5.83 | 10.78 | 8.38 | 6.13 | 6.13 | 6.17 |
| **S11** | 19.26 | 0.01 | 19.26 | 2.78 | 2.5 | 1.92 | 2.7 |
| **S12** | 11.95 | 0.35 | 11.6 | 3.93 | 3.62 | 3.58 | 3.81 |

**Table S6.** Force data acquired from participant 5.

| **Right Hand** | | | | | | | |
| --- | --- | --- | --- | --- | --- | --- | --- |
| **Sensor number** | **Max (N)** | **Min (N)** | **Range (N)** | **Time-averaged (N)** | **Median (N)** | **Q1 (N)** | **Q3 (N)** |
| **S1** | 18.51 | 1.46 | 17.05 | 5.4 | 4.62 | 4.4 | 4.69 |
| **S2** | 19.22 | 0 | 19.22 | 17.66 | 17.89 | 16.76 | 18.7 |
| **S3** | 20.05 | 2.59 | 17.46 | 9.91 | 9.11 | 9.02 | 9.6 |
| **S4** | 22 | 0 | 22 | 21.81 | 22 | 22 | 22 |
| **S5** | 21.92 | 0 | 21.92 | 14.83 | 14.39 | 13.31 | 14.82 |
| **S6** | 0.04 | 0 | 0.04 | 0 | 0 | 0 | 0 |
| **S7** | 11.7 | 0 | 11.7 | 11.53 | 11.62 | 11.61 | 11.62 |
| **S8** | 0.1 | 0 | 0.1 | 0 | 0 | 0 | 0 |
| **S9** | 3.98 | 0.66 | 3.32 | 0.91 | 0.66 | 0.66 | 0.83 |
| **S10** | 4.18 | 0 | 4.18 | 2.38 | 2.58 | 2.25 | 2.91 |
| **S11** | 12.03 | 0.82 | 11.21 | 3.79 | 2.21 | 1.89 | 4.24 |
| **S12** | 0 | 0 | 0 | 0 | 0 | 0 | 0 |
| **Left Hand** | | | | | | | |
| **Sensor number** | **Max (N)** | **Min (N)** | **Range (N)** | **Time-averaged (N)** | **Median (N)** | **Q1 (N)** | **Q3 (N)** |
| **S1** | 14.42 | 0.33 | 14.09 | 3.86 | 3.73 | 2.96 | 3.75 |
| **S2** | 4.34 | 1.45 | 2.89 | 1.92 | 1.74 | 1.74 | 1.99 |
| **S3** | 7.87 | 1.33 | 6.54 | 2.51 | 2.17 | 2.08 | 2.59 |
| **S4** | 20.18 | 1.18 | 19 | 11.17 | 11.24 | 9.83 | 11.94 |
| **S5** | 0.42 | 0 | 0.42 | 0.01 | 0 | 0 | 0 |
| **S6** | 15.15 | 1.06 | 14.09 | 4.38 | 1.83 | 1.81 | 7.78 |
| **S7** | 0 | 0 | 0 | 0 | 0 | 0 | 0 |
| **S8** | 0 | 0 | 0 | 0 | 0 | 0 | 0 |
| **S9** | 3.44 | 0 | 3.44 | 1.25 | 1.13 | 1.03 | 1.23 |
| **S10** | 22 | 0 | 22 | 16.14 | 21.93 | 7.85 | 22 |
| **S11** | 6.62 | 1.57 | 5.05 | 3.85 | 3.9 | 3.63 | 3.97 |
| **S12** | 2.49 | 0 | 2.49 | 1.72 | 1.66 | 1.35 | 2.25 |

**Table S7.** Force data acquired from participant 6.

| **Right Hand** | | | | | | | |
| --- | --- | --- | --- | --- | --- | --- | --- |
| **Sensor number** | **Max (N)** | **Min (N)** | **Range (N)** | **Time-averaged (N)** | **Median (N)** | **Q1 (N)** | **Q3 (N)** |
| **S1** | 10.99 | 0 | 10.99 | 3.28 | 1.99 | 1.74 | 3.23 |
| **S2** | 12.75 | 0 | 12.75 | 1.46 | 0 | 0 | 1.02 |
| **S3** | 15.44 | 0.33 | 15.11 | 7.69 | 6.78 | 5.22 | 10.06 |
| **S4** | 6.73 | 0.01 | 6.72 | 1.32 | 0.98 | 0.73 | 1.33 |
| **S5** | 21.72 | 0 | 21.72 | 4.59 | 1.78 | 1.02 | 6.54 |
| **S6** | 17.11 | 0 | 17.11 | 6.73 | 5.5 | 4.91 | 7.11 |
| **S7** | 6.36 | 0 | 6.36 | 0.21 | 0.02 | 0 | 0.1 |
| **S8** | 3.29 | 0 | 3.29 | 2.16 | 2.04 | 1.83 | 2.59 |
| **S9** | 16.58 | 3.7 | 12.88 | 6.62 | 5.95 | 3.86 | 7.87 |
| **S10** | 17.74 | 0 | 17.74 | 2.32 | 0.3 | 0.2 | 2.23 |
| **S11** | 18.45 | 0.03 | 18.41 | 6.03 | 5.94 | 3.61 | 7.83 |
| **S12** | 5.32 | 0 | 5.32 | 3.64 | 3.88 | 3.39 | 3.99 |
| **Left Hand** | | | | | | | |
| **Sensor number** | **Max (N)** | **Min (N)** | **Range (N)** | **Time-averaged (N)** | **Median (N)** | **Q1 (N)** | **Q3 (N)** |
| **S1** | 3.89 | 0.6 | 3.29 | 3.35 | 3.57 | 3.08 | 3.69 |
| **S2** | 2.27 | 0.35 | 1.91 | 1.5 | 1.64 | 1.02 | 2.02 |
| **S3** | 3.95 | 1.71 | 2.24 | 3.05 | 3.16 | 2.89 | 3.18 |
| **S4** | 7.18 | 0.02 | 7.16 | 6.08 | 6.45 | 5.56 | 6.88 |
| **S5** | 7.6 | 3.63 | 3.97 | 4.36 | 4.28 | 4.02 | 4.29 |
| **S6** | 5.9 | 0.57 | 5.33 | 5.48 | 5.49 | 5.38 | 5.68 |
| **S7** | 3.17 | 2.13 | 1.04 | 3.02 | 3.07 | 2.93 | 3.11 |
| **S8** | 2.79 | 1.59 | 1.2 | 2.51 | 2.69 | 2.3 | 2.78 |
| **S9** | 7.66 | 1.93 | 5.72 | 3.73 | 3.43 | 3.35 | 3.56 |
| **S10** | 6.23 | 5.83 | 0.4 | 6.19 | 6.2 | 6.18 | 6.2 |
| **S11** | 14.17 | 0 | 14.17 | 4.44 | 3.34 | 3.15 | 3.43 |
| **S12** | 4.28 | 0.34 | 3.94 | 3.64 | 3.67 | 3.62 | 3.78 |

**Table S8.** Force data acquired from participant 7.

| **Right Hand** | | | | | | | |
| --- | --- | --- | --- | --- | --- | --- | --- |
| **Sensor number** | **Max (N)** | **Min (N)** | **Range (N)** | **Time-averaged (N)** | **Median (N)** | **Q1 (N)** | **Q3 (N)** |
| **S1** | 16.66 | 0 | 16.66 | 3.21 | 1.96 | 1.49 | 3.89 |
| **S2** | 4.95 | 0 | 4.95 | 0.17 | 0 | 0 | 0 |
| **S3** | 15.29 | 0.33 | 14.96 | 4.77 | 3.42 | 1.64 | 8.43 |
| **S4** | 8.42 | 0 | 8.42 | 0.24 | 0 | 0 | 0 |
| **S5** | 22 | 0 | 22 | 7.34 | 0.5 | 0 | 20.34 |
| **S6** | 0.15 | 0 | 0.15 | 0 | 0 | 0 | 0 |
| **S7** | 3.13 | 0 | 3.13 | 0.4 | 0.12 | 0 | 0.58 |
| **S8** | 19.98 | 0 | 19.98 | 3.14 | 2.27 | 2.14 | 3.01 |
| **S9** | 15.55 | 3.7 | 11.85 | 5.7 | 4.95 | 4.58 | 5.54 |
| **S10** | 5.37 | 0 | 5.37 | 0.19 | 0 | 0 | 0 |
| **S11** | 0 | 0 | 0 | 0 | 0 | 0 | 0 |
| **S12** | 2.07 | 0 | 2.07 | 0.08 | 0 | 0 | 0 |
| **Left Hand** | | | | | | | |
| **Sensor number** | **Max (N)** | **Min (N)** | **Range (N)** | **Time-averaged (N)** | **Median (N)** | **Q1 (N)** | **Q3 (N)** |
| **S1** | 22 | 0.6 | 21.4 | 21.71 | 21.99 | 21.91 | 22 |
| **S2** | 13 | 0.44 | 12.55 | 9.81 | 9.79 | 8.78 | 11.43 |
| **S3** | 9.37 | 1.62 | 7.75 | 4.12 | 3.68 | 3.42 | 4.71 |
| **S4** | 0 | 0 | 0 | 0 | 0 | 0 | 0 |
| **S5** | 21.96 | 3.67 | 18.29 | 18.1 | 18.19 | 16.83 | 19.44 |
| **S6** | 21.69 | 0.54 | 21.16 | 16.07 | 15.66 | 14.8 | 16.91 |
| **S7** | 16.32 | 2.06 | 14.26 | 14.8 | 14.88 | 14.4 | 15.31 |
| **S8** | 22 | 1.59 | 20.41 | 21.84 | 22 | 22 | 22 |
| **S9** | 17.56 | 1.93 | 15.63 | 6.25 | 4.33 | 4.15 | 8.37 |
| **S10** | 7.79 | 5.92 | 1.87 | 6.52 | 6.49 | 6.15 | 6.66 |
| **S11** | 2.27 | 0 | 2.27 | 0.15 | 0.01 | 0 | 0.14 |
| **S12** | 2.28 | 0.34 | 1.94 | 0.4 | 0.34 | 0.34 | 0.36 |

**Table S9.** Force data acquired from participant 8.

| **Right Hand** | | | | | | | |
| --- | --- | --- | --- | --- | --- | --- | --- |
| **Sensor number** | **Max (N)** | **Min (N)** | **Range (N)** | **Time-averaged (N)** | **Median (N)** | **Q1 (N)** | **Q3 (N)** |
| **S1** | 14.86 | 0.96 | 13.89 | 4.2 | 3.72 | 3.03 | 4.48 |
| **S2** | 0.3 | 0.26 | 0.04 | 0.27 | 0.26 | 0.26 | 0.27 |
| **S3** | 14.3 | 0 | 14.3 | 3 | 0.48 | 0 | 6.58 |
| **S4** | 12.06 | 0.58 | 11.48 | 1.89 | 1.41 | 1.1 | 2.34 |
| **S5** | 21.79 | 0 | 21.79 | 5.43 | 1.54 | 0.11 | 10.54 |
| **S6** | 21.98 | 0 | 21.98 | 3.76 | 0.01 | 0 | 3.79 |
| **S7** | 3.43 | 0 | 3.43 | 1.22 | 0.81 | 0.25 | 2.34 |
| **S8** | 0 | 0 | 0 | 0 | 0 | 0 | 0 |
| **S9** | 22 | 0 | 22 | 11.87 | 13.62 | 0.62 | 21.31 |
| **S10** | 16.01 | 0 | 16.01 | 1.39 | 0.01 | 0 | 0.93 |
| **S11** | 22 | 0 | 22 | 4.57 | 1.35 | 0.01 | 6.13 |
| **S12** | 10.78 | 0 | 10.78 | 2.34 | 1.75 | 0.03 | 4.03 |
| **Left Hand** | | | | | | | |
| **Sensor number** | **Max (N)** | **Min (N)** | **Range (N)** | **Time-averaged (N)** | **Median (N)** | **Q1 (N)** | **Q3 (N)** |
| **S1** | 5.33 | 0 | 5.33 | 0.39 | 0 | 0 | 0.06 |
| **S2** | 0 | 0 | 0 | 0 | 0 | 0 | 0 |
| **S3** | 21.09 | 0 | 21.09 | 3.4 | 2.88 | 1.65 | 3.5 |
| **S4** | 15.5 | 0 | 15.5 | 0.5 | 0 | 0 | 0 |
| **S5** | 19.7 | 0 | 19.7 | 1.41 | 0.54 | 0.22 | 1.01 |
| **S6** | 12.72 | 0.01 | 12.72 | 2.02 | 0.87 | 0.62 | 2.83 |
| **S7** | 5.49 | 0.01 | 5.48 | 3.69 | 4.16 | 2.43 | 4.93 |
| **S8** | 13.98 | 0.08 | 13.9 | 5.96 | 6.15 | 4.36 | 7 |
| **S9** | 18.86 | 0.1 | 18.77 | 1.4 | 0.47 | 0.38 | 0.62 |
| **S10** | 21.95 | 0 | 21.95 | 5.05 | 4.61 | 3.08 | 6 |
| **S11** | 12.12 | 0 | 12.12 | 2.34 | 1.71 | 0.89 | 3.3 |
| **S12** | 14.71 | 0 | 14.71 | 1.45 | 0 | 0 | 2.61 |

**Table S10.** Force data acquired from participant 9.

| **Right Hand** | | | | | | | |
| --- | --- | --- | --- | --- | --- | --- | --- |
| **Sensor number** | **Max (N)** | **Min (N)** | **Range (N)** | **Time-averaged (N)** | **Median (N)** | **Q1 (N)** | **Q3 (N)** |
| **S1** | 3.33 | 0 | 3.33 | 0.25 | 0 | 0 | 0.1 |
| **S2** | 10.58 | 0 | 10.58 | 2.16 | 1.64 | 1.29 | 2.16 |
| **S3** | 10.77 | 1.29 | 9.48 | 3.39 | 2.8 | 2.28 | 3.9 |
| **S4** | 12.02 | 0 | 12.02 | 0.7 | 0 | 0 | 0.38 |
| **S5** | 12.92 | 3.14 | 9.78 | 4.22 | 3.79 | 3.51 | 4.14 |
| **S6** | 4.91 | 0 | 4.91 | 0.3 | 0 | 0 | 0.13 |
| **S7** | 8.75 | 0 | 8.75 | 0.82 | 0.05 | 0 | 0.98 |
| **S8** | 20.74 | 0 | 20.74 | 3.67 | 2.56 | 2.37 | 2.81 |
| **S9** | 6.34 | 0 | 6.34 | 1.56 | 0.79 | 0.63 | 2.44 |
| **S10** | 0.76 | 0 | 0.76 | 0.02 | 0 | 0 | 0 |
| **S11** | 4.64 | 0.85 | 3.79 | 1.64 | 1.47 | 1.17 | 1.64 |
| **S12** | 17.89 | 0 | 17.89 | 0.48 | 0.22 | 0.03 | 0.48 |
| **Left Hand** | | | | | | | |
| **Sensor number** | **Max (N)** | **Min (N)** | **Range (N)** | **Time-averaged (N)** | **Median (N)** | **Q1 (N)** | **Q3 (N)** |
| **S1** | 7.08 | 0 | 7.08 | 0.81 | 0.56 | 0.21 | 0.77 |
| **S2** | 2.03 | 0.06 | 1.97 | 1.42 | 1.4 | 1.26 | 1.64 |
| **S3** | 19.04 | 0 | 19.04 | 3.87 | 3.15 | 1.82 | 5.93 |
| **S4** | 0 | 0 | 0 | 0 | 0 | 0 | 0 |
| **S5** | 17.79 | 0.01 | 17.78 | 3.06 | 1.39 | 1.01 | 4.14 |
| **S6** | 0.61 | 0 | 0.61 | 0.08 | 0 | 0 | 0.03 |
| **S7** | 2.11 | 0.01 | 2.1 | 0.84 | 0.44 | 0.32 | 1.54 |
| **S8** | 0.82 | 0 | 0.85 | 0.18 | 0.13 | 0.04 | 0.25 |
| **S9** | 6.3 | 0 | 6.31 | 0.54 | 0.16 | 0 | 0.47 |
| **S10** | 1.06 | 0 | 1.06 | 0.2 | 0 | 0 | 0.33 |
| **S11** | 3.5 | 1.16 | 2.33 | 1.54 | 1.54 | 1.45 | 1.59 |
| **S12** | 0.21 | 0 | 0.21 | 0.01 | 0 | 0 | 0 |

**Table S11.** Force data acquired from participant 10.

| **Right Hand** | | | | | | | |
| --- | --- | --- | --- | --- | --- | --- | --- |
| **Sensor number** | **Max (N)** | **Min (N)** | **Range (N)** | **Time-averaged (N)** | **Median (N)** | **Q1 (N)** | **Q3 (N)** |
| **S1** | 3.64 | 0 | 3.64 | 0.32 | 0 | 0 | 0.09 |
| **S2** | 21.99 | 0 | 21.99 | 5.41 | 2.51 | 0.97 | 5.19 |
| **S3** | 15.22 | 1.28 | 13.94 | 4.4 | 2.8 | 2.23 | 5.55 |
| **S4** | 21.74 | 0 | 21.74 | 2.29 | 0.17 | 0.01 | 0.55 |
| **S5** | 7.3 | 3.14 | 4.16 | 4.29 | 3.98 | 3.64 | 4.67 |
| **S6** | 9.37 | 0 | 9.37 | 0.75 | 0 | 0 | 0.13 |
| **S7** | 17.45 | 0 | 17.45 | 1.65 | 1.02 | 0.67 | 1.8 |
| **S8** | 22 | 0 | 22 | 4.64 | 3.09 | 2.8 | 3.43 |
| **S9** | 12.55 | 0 | 12.55 | 2.64 | 1.7 | 0.68 | 3.52 |
| **S10** | 10.14 | 0 | 10.14 | 0.46 | 0 | 0 | 0 |
| **S11** | 17.76 | 0.85 | 16.91 | 3.96 | 2.02 | 1.49 | 5.38 |
| **S12** | 0.48 | 0 | 0.48 | 0.02 | 0 | 0 | 0.04 |
| **Left Hand** | | | | | | | |
| **Sensor number** | **Max (N)** | **Min (N)** | **Range (N)** | **Time-averaged (N)** | **Median (N)** | **Q1 (N)** | **Q3 (N)** |
| **S1** | 0.51 | 0 | 0.51 | 0.18 | 0.15 | 0.06 | 0.3 |
| **S2** | 17.68 | 0 | 17.68 | 1.09 | 0.84 | 0.2 | 1.22 |
| **S3** | 21.4 | 0 | 21.4 | 2.82 | 1.99 | 1.25 | 2.82 |
| **S4** | 0.11 | 0 | 0.11 | 0 | 0 | 0 | 0 |
| **S5** | 5.24 | 0.02 | 5.22 | 1.32 | 1.22 | 1.03 | 1.4 |
| **S6** | 0 | 0 | 0 | 0 | 0 | 0 | 0 |
| **S7** | 2.22 | 0.21 | 2.01 | 1.72 | 1.85 | 1.58 | 2.04 |
| **S8** | 0.75 | 0 | 0.75 | 0.54 | 0.59 | 0.54 | 0.72 |
| **S9** | 2.1 | 0 | 2.1 | 0.27 | 0.04 | 0 | 0.51 |
| **S10** | 7.48 | 0 | 7.48 | 0.38 | 0 | 0 | 0.51 |
| **S11** | 2.08 | 1.18 | 0.89 | 1.46 | 1.39 | 1.31 | 1.54 |
| **S12** | 5.36 | 0 | 5.36 | 0.16 | 0 | 0 | 0 |

**Table S12.** Force data acquired from participant 11.

| **Right Hand** | | | | | | | |
| --- | --- | --- | --- | --- | --- | --- | --- |
| **Sensor number** | **Max (N)** | **Min (N)** | **Range (N)** | **Time-averaged (N)** | **Median (N)** | **Q1 (N)** | **Q3 (N)** |
| **S1** | 17.46 | 1.24 | 16.22 | 2.97 | 2.03 | 1.85 | 2.51 |
| **S2** | 21.95 | 0 | 21.95 | 8.22 | 2.23 | 0 | 18.8 |
| **S3** | 18.12 | 3.86 | 14.25 | 10.49 | 11.01 | 6.39 | 13.44 |
| **S4** | 21.36 | 0.06 | 21.3 | 5.09 | 3.12 | 1.38 | 6.87 |
| **S5** | 22 | 0.93 | 21.07 | 11.99 | 11.2 | 2.42 | 21.98 |
| **S6** | 21 | 3.16 | 17.85 | 9.12 | 7.24 | 4.96 | 12.96 |
| **S7** | 20.87 | 0 | 20.87 | 3.36 | 0.1 | 0 | 4.19 |
| **S8** | 18.05 | 0.63 | 17.42 | 4.15 | 2.71 | 1.01 | 5.43 |
| **S9** | 22 | 3.7 | 18.3 | 11.57 | 10.87 | 3.71 | 18.27 |
| **S10** | 15.87 | 0 | 15.87 | 1.65 | 0.08 | 0 | 1.6 |
| **S11** | 16.38 | 0 | 16.38 | 7.38 | 5.76 | 1.75 | 13.01 |
| **S12** | 6.99 | 0.6 | 6.39 | 3.32 | 3.38 | 3.02 | 3.85 |
| **Left Hand** | | | | | | | |
| **Sensor number** | **Max (N)** | **Min (N)** | **Range (N)** | **Time-averaged (N)** | **Median (N)** | **Q1 (N)** | **Q3 (N)** |
| **S1** | 4.95 | 1.51 | 3.44 | 2.83 | 2.9 | 2.33 | 3.35 |
| **S2** | 7.05 | 1.58 | 5.47 | 1.96 | 1.81 | 1.74 | 1.94 |
| **S3** | 13.89 | 2.26 | 11.63 | 5.22 | 4.31 | 2.55 | 7.49 |
| **S4** | 20.19 | 6 | 14.19 | 10.08 | 8.72 | 8.49 | 10.42 |
| **S5** | 0 | 0 | 0 | 0 | 0 | 0 | 0 |
| **S6** | 9.16 | 1.87 | 7.3 | 7.87 | 8.05 | 7.79 | 8.43 |
| **S7** | 0 | 0 | 0 | 0 | 0 | 0 | 0 |
| **S8** | 0 | 0 | 0 | 0 | 0 | 0 | 0 |
| **S9** | 7.18 | 0.7 | 6.48 | 1.21 | 1.04 | 0.86 | 1.2 |
| **S10** | 13.35 | 0 | 13.35 | 0.26 | 0 | 0 | 0 |
| **S11** | 6.28 | 3.58 | 2.69 | 4.8 | 4.93 | 4.44 | 5.1 |
| **S12** | 11.4 | 0.73 | 10.66 | 2.99 | 2.37 | 1.46 | 4.35 |

**Table S13.** Force data acquired from participant 12.

| **Right Hand** | | | | | | | |
| --- | --- | --- | --- | --- | --- | --- | --- |
| **Sensor number** | **Max (N)** | **Min (N)** | **Range (N)** | **Time-averaged (N)** | **Median (N)** | **Q1 (N)** | **Q3 (N)** |
| **S1** | 4.05 | 0 | 4.05 | 0.36 | 0.1 | 0.01 | 0.41 |
| **S2** | 21.77 | 0 | 21.77 | 3.7 | 1.36 | 0.85 | 2.44 |
| **S3** | 18.06 | 1.28 | 16.78 | 4.27 | 2.44 | 2.27 | 6.1 |
| **S4** | 22 | 0 | 22 | 2.45 | 0.53 | 0 | 2.76 |
| **S5** | 6.8 | 3.14 | 3.66 | 4.33 | 3.7 | 3.59 | 5.26 |
| **S6** | 13.4 | 0 | 13.4 | 0.58 | 0 | 0 | 0.04 |
| **S7** | 1.38 | 0 | 1.38 | 0.01 | 0 | 0 | 0 |
| **S8** | 18.46 | 0 | 18.46 | 3.49 | 2.18 | 1.87 | 2.51 |
| **S9** | 14.04 | 0 | 14.04 | 2.8 | 0.99 | 0.49 | 4.65 |
| **S10** | 9.85 | 0 | 9.85 | 1.47 | 0 | 0 | 1.53 |
| **S11** | 16.98 | 0.85 | 16.13 | 3.8 | 1.5 | 1.17 | 6.13 |
| **S12** | 0.11 | 0 | 0.11 | 0.01 | 0 | 0 | 0 |
| **Left Hand** | | | | | | | |
| **Sensor number** | **Max (N)** | **Min (N)** | **Range (N)** | **Time-averaged (N)** | **Median (N)** | **Q1 (N)** | **Q3 (N)** |
| **S1** | 8.52 | 0 | 8.52 | 0.92 | 0.56 | 0.3 | 0.67 |
| **S2** | 0.82 | 0 | 0.82 | 0.14 | 0.03 | 0 | 0.26 |
| **S3** | 10.35 | 0 | 10.35 | 1.71 | 1.7 | 0 | 2.37 |
| **S4** | 4.38 | 0 | 4.38 | 0.19 | 0 | 0 | 0 |
| **S5** | 13.63 | 0 | 13.63 | 1.64 | 1.21 | 0.99 | 1.49 |
| **S6** | 0 | 0 | 0 | 0 | 0 | 0 | 0 |
| **S7** | 2.13 | 0 | 2.13 | 1.84 | 1.88 | 1.77 | 2.04 |
| **S8** | 0.68 | 0 | 0.68 | 0.2 | 0.18 | 0.04 | 0.32 |
| **S9** | 3.58 | 0 | 3.58 | 0.36 | 0.1 | 0.01 | 0.44 |
| **S10** | 11.59 | 0 | 11.59 | 0.87 | 0.58 | 0.44 | 0.77 |
| **S11** | 3.61 | 1.15 | 2.46 | 1.5 | 1.46 | 1.38 | 1.55 |
| **S12** | 2.11 | 0 | 2.11 | 0.02 | 0 | 0 | 0 |

**Table S14.** Force data acquired from participant 13.

**Appendix 5.**

# Feature Extraction

To deconstruct the complex phenomenon of motor skill into quantifiable components, a hierarchical feature set was systematically engineered from the time series of force sensor data during disimpaction in simulated childbirth. This high-dimensional feature framework is designed to capture distinct, complementary aspects of motor control, spanning from localized dynamics to the emergent properties of inter-limb coordination. The features are presented across five categories encompassing different aspects of sensor analysis of bimanual control:

## Comprehensive Feature Set for Skill Analysis

1. **Spatiotemporal Force Dynamics at Individual Sensor Locations**

This foundational category comprises metrics calculated from the univariate time- series of each individual sensor 𝐹_𝑖_(𝑡), where *i* is the sensor index and *t* is time. These features provide a granular characterization of the force production at specific sensor- skin interfaces.

## First- and Second-Moment Statistical Descriptors

- - **Tonic Force Component**
    - **Definition:** The arithmetic mean of the force signal 𝐹_𝑖_(𝑡) over the trial duration T.

## Equation: 𝜇

𝐹𝑖

= 1 ∑^𝑀^ 𝐹ᵢ(𝑡ⱼ) (1)

𝑀

𝑗

- - - **Rationale:** This metric quantifies the tonic component of neuromuscular activation. It reflects the baseline, sustained level of force. Skill acquisition often involves transitioning from a novice strategy of high co- contraction (elevated, inefficient 𝜇_𝐹_𝑖 to an expert strategy where 𝜇_𝐹_𝑖 is optimized to the minimum effective level.

## Peak Phasic Force

- - - **Definition:** The global maximum of the force signal 𝐹_𝑖_(𝑡).
    - **Equation:** 𝐹_𝑖,𝑚𝑎𝑥_ = 𝑚𝑎𝑥_𝑡_ _∈_ _[0,𝑇]_𝐹_𝑖_(𝑡) (2)
    - **Rationale:** This isolates the peak phasic component of force generation. Skill refinement is often associated with modulation of 𝐹_𝑖,𝑚𝑎𝑥_ toward a task-optimal, consistent level.

## Force Output Variability

- - - **Definition:** The standard deviation of the force signal 𝐹_𝑖_(𝑡).
    - **Equation:** 𝜎_𝐹_𝑖 = √(

^1^ ) ∑

2

( 𝐹𝑖(𝑡𝑗) − 𝜇𝐹𝑖)

(3)

𝑀−1

- - - **Rationale:** Quantifies the stability and precision of force control. Lower 𝜎_𝐹_𝑖 indicates a higher signal-to-noise ratio and expert-level control.

## Higher-Order Distributional Shape Descriptors

- - **Force Distribution Asymmetry**
    - **Definition:** Third standardized moment of the force signal’s distribution (skew).

## Equation:

1 ∑(𝐹 (𝑡 ) − μ )3

γ(𝐹𝑖)

= 𝑀

𝑖 𝑗

σ 3

𝐹𝑖

(4)

𝐹𝑖

- - - **Rationale:** Positive skew indicates ballistic actions (low baseline punctuated by rapid, high-amplitude pulses). Consistent non-zero skewness reflects a stereotyped, task-specific motor signature.

## Force Distribution Tailedness

- - - **Definition:** Fourth standardized moment of the force signal’s distribution (Kurtosis).

## Equation:

1 ∑(𝐹 (𝑡 ) − μ )4

κ(𝐹𝑖)

= 𝑀

𝑖 𝑗

σ 4

𝐹𝑖

(5)

𝐹𝑖

- - - **Rationale:** Kurtosis measures outlier prevalence. High-kurtosis (leptokurtic) signals are concentrated around the mean with infrequent significant deviations.

## Temporal and Energetic Descriptors

- - **Cumulative Impulse & Normalized Rate**
    - **Definition:** The total impulse is the numerical integration (Area Under the Curve) of 𝐹_𝑖_(𝑡)over the trial duration. The normalized rate is this total impulse divided by the trial duration T.

## Equation: 𝐼

= ∫𝑇 𝐹 (𝑡) 𝑑𝑡 ≈ ∑𝑀

𝐹 (𝑡 ) Δ𝑡; 𝐼

= 𝐼𝐹𝑖

(6)

𝐹𝑖

0 𝑖

𝑗=1 𝑖 𝑗

𝐹𝑖,rate 𝑇

- - - **Rationale:** Total impulse represents the total energetic effort delivered at a sensor location. Normalizing it by time creates an impulse rate (a proxy for average power), which is critical for comparing trials of different lengths. It disentangles overall effort from the intensity of that effort over time.

## Peak Force Latency

- - - **Definition:** The time *t* at which 𝐹_𝑖,𝑚𝑎𝑥_ is achieved.
    - **Equation:** 𝑡𝑖,max = 𝑎𝑟𝑔 𝑚𝑎𝑥𝑡∈[0,𝑇]{𝐹𝑖(𝑡)} (7)
    - **Rationale:** Serves as a temporal anchor in motor execution. Experts show low variability in 𝑡_𝑖,max_.

## Intra-Limb Synergistic Control

Moves from individual sensors to the limb, quantifying the degree of integration across effectors.

## Temporal Synergism

- - - **Definition:** Standard deviation of peak force latencies 𝑡_𝑖,max_ across *k* sensors.

## Equation: σ

= √ ∑

1 𝑘

2

(𝑡 − μ

𝑡max

𝑘−1

𝑖=1

𝑖,max

𝑡max )

(8)

- - - **Rationale:** Quantifies temporal cohesion. Low 𝜎_𝑡_max indicates synchronous activation.

## Suprathreshold Effort Integral (RH exceedance integral)

- - - **Definition:** Force-time integral for 𝐹_𝑖_(𝑡) exceeding a task-relevant threshold 𝐹_thresh_.

## Equation: 𝐼

= ∑𝑘 ∫𝑇 𝐻(𝐹 (𝑡) − 𝐹

) 𝐹 (𝑡) 𝑑𝑡 (9)

exceed

𝑖=1 0 𝑖

thresh 𝑖

where H(x) is the Heaviside step function.

- - - **Rationale:** Isolates task-salient force. More accurate assessment of active, goal-directed energetic cost.

## Aggregated Limb Performance Signatures

Features derived from the aggregated signal of all sensors on one limb: 𝐹̅(𝑡) =

^1^ ∑^𝑘^ 𝐹 (𝑡)

𝑘 𝑖=1 𝑖

## Spectral Smoothness Index

- **Definition:** The ratio of power in a low-frequency band (e.g., 0-2 Hz) to the power in a higher-frequency band (e.g., 3-10 Hz) of the aggregated signal's power spectrum, 𝑃(𝑓).

∫𝑓𝐿2 𝑃(𝑓) 𝑑𝑓

- **Equation:** Smoothness Ratio = ^𝑓𝐿1^ (10)

∫𝑓𝐻2 𝑃(𝑓) 𝑑𝑓+𝝴

𝑓𝐻1

- **Rationale:** A higher ratio indicates that most of the signal's power is concentrated in low frequencies, corresponding to smoother, more controlled, and efficient movements characteristic of experts.

## Kinematic Jerk Analogue

- - **Definition:** The time-averaged magnitude of the third derivative of the force signal, approximated by the second-order finite difference of 𝐹̅(𝑡).
  - **Equation:** Jerk Analogue =

∑𝑀−1 |𝐹̅(𝑡𝑗+1)−2𝐹̅(𝑡𝑗)+𝐹̅(𝑡𝑗−1)| (11)

𝑀−2

1

𝑗=2

(Δ𝑡)2

- - **Rationale:** Lower values signify a smoother force trajectory with fewer abrupt changes in acceleration, a hallmark of expert-like motor control.

## Movement Rhythmicity (Peak Count, Peak Count per sec)

- - **Definition:** The total number of significant force peaks within the aggregated signal 𝐹̅(𝑡). Peaks are defined as local maxima that exceed a height threshold relative to the signal's range and are separated by a minimum time distance. The peak rate is this count divided by the trial duration T.
  - **Equation:** Let P be the set of time points corresponding to identified

peaks 𝑁_peaks_

= |𝑃|; 𝑅_peaks_

= 𝑁peaks

𝑇

(12)

- - **Rationale:** This metric quantifies the discreteness versus continuity of the motor strategy. A high peak count often reflects a novice's hesitant, multi-part movement, whereas a low count (ideally one) reflects a consolidated, efficient, and ballistic action typical of an expert. The rate normalizes this for comparison across trials of different durations.

## Force Trajectory Efficiency (Path Length, Path Length per sec)

- - **Definition:** The cumulative sum of the absolute differences between consecutive points in the aggregated force signal F̄(t). The normalized version divides this total path length by the trial duration T.

## Equation:

𝑀−1

Path Length = ∑|𝐹̅(𝑡_𝑗+1_) − 𝐹̅(𝑡_𝑗_)| ; Path Lengthrate

𝑗=1

Path Length

= (13)

𝑇

- - **Rationale:** A lower total path length indicates a more direct and efficient force trajectory from initiation to completion. Normalizing by time provides a measure of the average speed of force modulation, helping to distinguish between efficient slowness and inefficient, rapid adjustments.

## Signal Complexity (Sample Entropy)

- - **Definition:** A non-linear measure of the predictability of a time-series, calculated as the negative natural logarithm of the conditional probability that two similar sequences of *m* points remain similar at the next point.
  - **Equation:** Entropy(

𝑚, 𝑟, 𝑁)

= − ln ! (𝐴

) (14)

𝐵

- - **Rationale:** Tests the "loss of complexity" hypothesis in motor learning, where expert performance is often associated with a more regular, less complex (lower entropy) signal, indicating a well-practiced and automated motor pattern.

## Pattern Similarity to Archetype (DTW distance)

- - **Definition:** The optimal alignment cost between a participant's aggregated force profile 𝐹̅_𝑝_(𝑡) and an expert's archetypal profile 𝐹̅_𝑒_(𝑡), computed using Dynamic Time Warping (DTW).

## Equation:

𝐷(𝑗, 𝑙) = cost(𝑗, 𝑙) + min(𝐷(𝑗 − 1, 𝑙), 𝐷(𝑗, 𝑙 − 1), 𝐷(𝑗 − 1, 𝑙 − 1)) (15)

- - where cost(𝑗, 𝑙) is the Euclidean distance between 𝐹̅_𝑝_(𝑡_𝑗_) and 𝐹̅_𝑒_(𝑡_𝑙_).
  - **Rationale:** Provides a quantitative measure of how closely a participant's movement pattern matches the "gold standard" expert template, robust to minor variations in timing and speed. A lower distance indicates greater similarity to the expert.

## Inter-Limb Coordination

Compares the aggregated right-hand 𝐹̅̅_𝑅̅𝐻̅̅_̅(𝑡) and left-hand 𝐹̅̅_𝑅̅𝐻̅̅_̅(𝑡) signals.

## Inter-limb Effort Symmetry (Dominance Ratio integral)

- - - **Definition:** The ratio of the total impulse (integral) of the right hand to that of the left hand.
    - **Equation:** Effort Ratio = 𝐼𝐹̅̅𝑅̅̅𝐻̅̅̅

𝐼𝐹̅̅𝐿̅̅𝐻̅̅̅

(16)

- - - **Rationale:** Quantifies the balance of work done between the two limbs. A value near 1.0 indicates symmetric effort, while significant deviation reveals asymmetric control strategies, which may be task-dependent or indicative of skill level.

## Inter-limb Pattern Isomorphism (Maximum Normalized Cross Correlation)

- - - **Definition:** The maximum value of the normalized cross-correlation function between the two aggregated limb signals over all possible time lags (τ).
    - **Equation:** 𝑅 = Max Correlation = max {∫(𝐹𝑅𝐻(𝑡)−𝜇𝑅𝐻)(𝐹𝐿𝐻(𝑡+𝜏)−𝜇𝐿𝐻) 𝑑𝑡} (17)

τ 𝜎𝑅𝐻 𝜎𝐿𝐻

- - - **Rationale:** Measures the degree of linear similarity in the force patterns produced by the two hands, irrespective of timing. A high value indicates strong coupling and coordinated control.

## Inter-limb Temporal Lead-Lag (Lag at maximum correlation)

- - - **Definition:** The time lag (τ) at which the maximum cross-correlation occurs.
    - **Equation:** Lag (τ) = 𝑎𝑟𝑔 𝑚𝑎𝑥_τ_{𝑅(τ)} (18)
    - **Rationale:** Reveals which limb leads the other in the coordinated action. The sign and magnitude of the lag are directly linked to interhemispheric communication and the temporal structure of the bimanual skill.

## Dynamical Systems Analysis of Bimanual Coordination

Inspired by the Haken-Kelso-Bunz (HKB) model, these features describe the coordination dynamics between the two limbs as coupled oscillators by analyzing their relative phase: 𝛟(𝑡) = θ_𝑅𝐻_(𝑡) − θ_𝐿𝐻_(𝑡).

## Coordination Instability (HKB Phase Variability)

- **Definition:** The circular standard deviation of the wrapped relative phase signal 𝜙_𝑤_(𝑡) over the trial.

## Equation: σ

= √ 1 ∑^𝑀^

(𝛟

2

(𝑡 ) − μ

𝛟 𝑀−1

𝑗=1

𝑤 𝑗

𝛟𝑤)

(19)

- **Rationale:** Quantifies the stability of the bimanual coordination pattern. Lower variability (low 𝜎_𝜙_) indicates a more stable and consistent phase relationship, characteristic of expert performance. Higher variability signals instability or transitions between coordination modes.

## In-Phase Attractor Strength (HKB In Phase Attraction)

- - **Definition:** The proportion of the trial duration during which the relative phase resides within a narrow window around 0 radians (e.g., ±π/4).
  - **Equation:** In-Phase Attraction = 1 ∫𝑇 𝐻 (π − |𝛟 (𝑡)|) 𝑑𝑡 (20)

𝑇 0 4 𝑤

- - **Rationale:** Measures the strength and prevalence of the in-phase (symmetric) coordination mode. A high value indicates a dominant and stable in-phase pattern.

## Anti-Phase Attractor Strength (HKB Anti Phase Attraction)

- - **Definition:** The proportion of the trial duration during which the relative phase resides within a narrow window around ±π radians.
  - **Equation:** Anti-Phase Attraction = 1 ∫𝑇 𝐻 (π − ||𝛟 (𝑡)| − 𝜋|) 𝑑𝑡 (21)

𝑇 0 4 𝑤

- - **Rationale:** Measures the strength and prevalence of the anti-phase (asymmetric) coordination mode.

## Coordination Modality (HKB Phase Modality Index)

- - **Definition:** An index that quantifies the relative dominance of the in- phase versus anti-phase coordination modes.
  - **Equation:** Modality Index = max(𝑃in,𝑃anti) (22)

𝑃in+𝑃anti+𝝴

- - **Rationale:** A value near 0.5 indicates bistability (both modes are equally present), while a value near 1.0 indicates monostability (one mode strongly dominates the other), revealing the underlying dynamical landscape of the coordination task.

## Data analysis: PCA and ML

To elucidate the underlying structure within the high-dimensional feature set, Principal Component Analysis (PCA) was first employed to reduce the data into its most salient dimensions of variance. The problem was framed as a binary classification task. Participants (N=11) were categorized into two classes based on their career level: a) Non-senior (Class 0): Trainees at levels ST1-2 and ST3-5; and b) Senior (Class 1): Trainees at level ST6-7 and Consultants. The equivalent for perceived difficulty was Easy, Normal (Class 0) and Normal/Hard, Hard (Class 1). Notably, the participants who performed the disimpaction with the aid of another clinician were not included and there was one participant who performed the task twice.

When the resulting principal components were stratified by clinical seniority (Figure S28a), no discernible clustering or gradient was observed. Senior and junior participants were intermingled across the principal component space, indicating that seniority alone did not account for the primary sources of variance in performance. This lack of separation was quantitatively confirmed by a chi-squared test, which showed no significant association between the data-driven performance clusters and the seniority groups (𝜒^2^ = 0.88, P-value = 0.35).

In contrast, a more distinct pattern emerged when the data was stratified by the participants' self-reported perceived difficulty (Figure S28b). A clear trend was observed where participants who perceived the task as 'Hard' predominantly clustered on the right side of the plot, suggesting a common underlying neuromechanical signature associated with higher subjective task demand. This visual grouping was also statistically significant, as a chi-squared test confirmed a strong association between performance clusters and the perceived difficulty groups (𝜒^2^ = 2.75, P-value

= 0.097).

Furthermore, the only participant who did a second trial seemed to be nearly identical in the 2D principal component (PC) space (using the two first PCs), which shows the value of the feature matrix constructed. This is also supported by the fact that relying purely on the averages and peaks of the forces applied were not sufficient parameters to discern this.


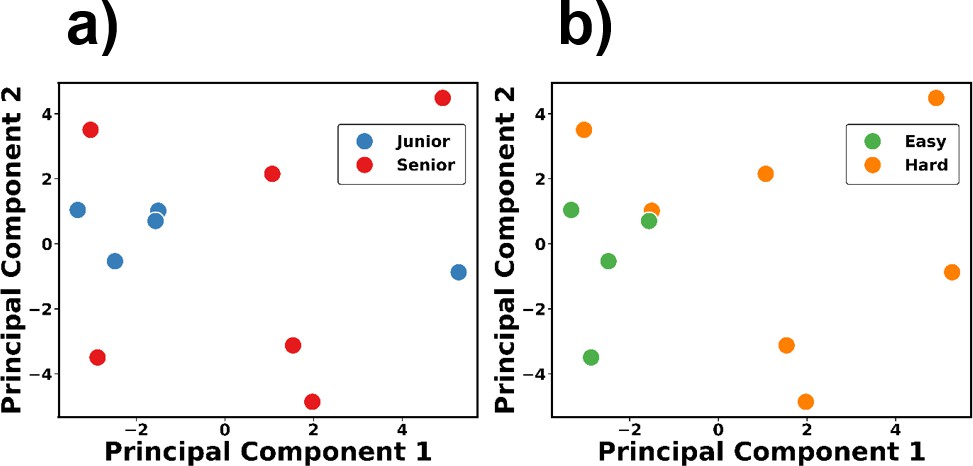


**Figure S28.** Principal Component Analysis (PCA) of all performance features stratified by a) Clinician Seniority, where the participants where split into two groups Junior and Senior from the original 4 (ST1-2, ST3-5, ST6-7, Consultant); and b) Perceived Difficulty, where the participants were split into two groups of Easy and Hard from the original 4 ( Less Force / Easy, Normal, Normal hard, Hard). The two principal components (PCs) were derived from all standardized performance features. Each point represents an individual participant, positioned according to their composite performance score. Participants are colored by their seniority level and self-reported perceived difficulty of the disimpaction task.

To further investigate the patterns suggested by the PCA, we moved from descriptive visualization to quantitative predictive modelling. Supervised machine learning classifiers were trained on the comprehensive feature set to formally test whether a predictive signal existed for either clinical seniority or perceived difficulty. We evaluated two distinct models to ensure robustness: a linear Logistic Regression and a non- linear ensemble model, the Random Forest classifier. To ensure an unbiased evaluation, we employed a rigorous Leave-One-Out Cross-Validation (LOOCV) strategy and bootstrapping due to the small sample size of participants (N=11).

Finally, we used the Receiver Operating Characteristic (ROC) curve a graphical plot that illustrates the diagnostic ability of a binary classifier as its discrimination threshold is varied. The curve plots two parameters: i) True Positive Rate (TPR) on the y-axis: The proportion of actual positives that are correctly identified as such (TPR = TP / (TP

+ FN)). Also known as sensitivity or recall ; and ii) False Positive Rate (FPR) on the x- axis: The proportion of actual negatives that are incorrectly identified as positive (FPR

= FP / (FP + TN)). The Area Under the Curve (AUC) provides a single scalar value that summarizes the overall performance of the classifier across all possible thresholds. The AUC value ranges from 0 to 1 and represents the probability that the model will rank a randomly chosen positive instance higher than a randomly chosen negative instance. For an AUC = 0.5 the model has no discriminative ability, equivalent to random guessing. A diagonal line on the ROC plot represents this. The AUC is a particularly robust metric for evaluating classifiers, especially on datasets with a class imbalance.

Consistent with the PCA findings, all models failed to classify clinician seniority with performance better than random chance (AUC ≈ 0.5). However, a higher predictive signal was identified for the Perceived Difficulty classification; both Logistic Regression and the Random Forest model achieved the much higher Area Under the Curves (AUCs) of 0.82 and 0.91 (with 1 being the perfect predictor), respectively (Figure S29). It should be underlined that due to the limited number of participants we could not establish definitive conclusions.


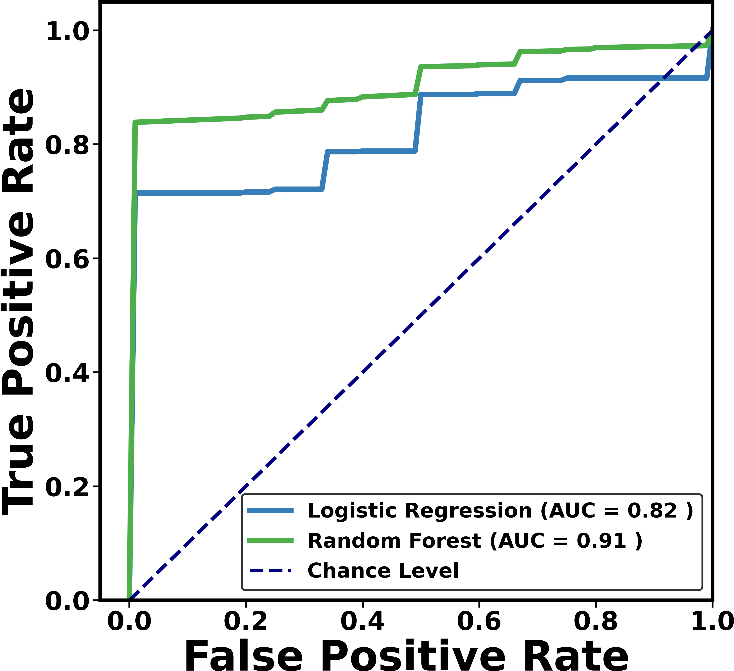


**Figure S29.** Comparative Machine Learning Performance for Predicting Perceived Difficulty. Receiver Operating Characteristic (ROC) curves for Logistic Regression and Random Forest models evaluated using a Leave-One- Out Cross-Validation (LOOCV) and Bootstrapping strategies. The y-axis represents the True Positive Rate (Sensitivity), and the x-axis represents the False Positive Rate (1 - Specificity). The Area Under the Curve (AUC) quantifies the model's overall ability to distinguish between the 'Hard' and 'Easy' perceived difficulty classes. The classifiers yielded an AUC ROC of 0.82 and 0.91 for the Logistic Regression and Random Forest models, respectively. The dashed line represents the performance of a random-chance classifier (AUC = 0.5).

Taken together, these findings strongly suggest that for this complex bimanual task, the subjective experience of difficulty was a more powerful determinant of the measured performance profile than the objective measure of clinical experience. Consequently, these results highlight the critical importance of accounting for perceived task difficulty as a key confounding variable or stratification factor in future studies of motor skill assessment.

# Timeseries analysis

Building upon the descriptive statistical analysis presented in the main manuscript, a computational study was undertaken to investigate whether dynamic temporal patterns within the force-time series data could objectively classify clinician seniority. The primary hypothesis was that expertise is encoded not merely in static force metrics

(e.g., maximum or average force) but in the sequential patterns of force application over time. The objective was to develop and validate a machine learning model to automatically distinguish between ‘Senior’ and ‘Non-senior’ clinicians and ‘Hard’ and ‘Easy’ perceived level of difficulty, based solely on the raw time series data captured by the sensorised glove.

The raw time-series data from the 12 sensors on the left and right hands were combined for each participant by concatenating the sensor channels into a single multivariate time series. To ensure uniform input length for the model, all sequences were padded to the length of the longest recorded procedure. The participants who performed the disimpaction with the aid of another clinician were once more not included. To account for inter-participant variations in baseline sensor readings, the data for each participant was independently standardized using a StandardScaler. A Recurrent Neural Network (RNN) was selected for this task. Unlike models that process data points independently, RNNs are specifically designed to operate on sequential data. They possess an internal memory (or hidden state) which allows them to retain information from previous time steps while processing the current one. This architecture makes them highly suitable for learning the temporal dependencies and dynamic patterns within the nuanced, time-dependent maneuvers involved in a complex procedure like fetal head disimpaction.

Given the limited sample size (N=11), a rigorous Leave-One-Out Cross-Validation (LOOCV) strategy was employed to ensure a robust and unbiased evaluation of model performance. Furthermore, a systematic Hyperparameter Optimization (HPO) process was conducted using the Optuna framework. This process automatically searched for the optimal model architecture (e.g., number of RNN layers, hidden unit size) and training parameters (e.g., learning rate, dropout rate) by repeatedly evaluating different configurations against the mean accuracy achieved across the full LOOCV procedure. This ensured that the final reported performance is from the best-performing model configuration.

The performance of the final, optimized RNN model was evaluated using standard classification metrics, including accuracy, precision, recall, F1-score, and the AUC. The model yielded an AUC≤0.5, with overall performance metrics indicating that it was unable to reliably distinguish between the two seniority classes. This result suggests that, within the constraints of the available dataset and the chosen model architecture, a consistent and generalizable temporal signal for clinical seniority was not identified. The performance, being slightly below the random-chance baseline of 0.5 AUC, indicates that the model could not find a valid, generalizable pattern among participants.

Similar results were shown for perceived difficulty classification as the AUC ROC was once more found to be >0.5. This outcome highlights the significant challenges of applying deep learning models to small biomedical datasets, where the risk of

overfitting to noise is high. Hence, this underlines the importance of extracting and building higher level featured-based models as in the previous section.

This foundational work underscores the necessity for a larger participant cohort to provide the statistical power required for deep learning models and thus future work should include a larger cohort of participants for statistically meaningful conclusions.
